# Discovery of Thiophene Derivatives as Potent, Orally Bioavailable, and Blood–Brain Barrier-Permeable Ebola Virus Entry Inhibitors

**DOI:** 10.1021/acs.jmedchem.4c01267

**Published:** 2024-09-09

**Authors:** Marcos Morales-Tenorio, Fátima Lasala, Alfonso Garcia-Rubia, Elnaz Aledavood, Michelle Heung, Catherine Olal, Beatriz Escudero-Pérez, Covadonga Alonso, Ana Martínez, César Muñoz-Fontela, Rafael Delgado, Carmen Gil

**Affiliations:** † Centro de Investigaciones Biológicas Margarita Salas (CIB-CSIC), Madrid 28040, Spain; ‡ Instituto de Investigación Hospital 12 de Octubre,, Madrid 28041, Spain; § Bernhard Nocht Institute for Tropical Medicine, Hamburg 20359, Germany; ∥ Dpt. Biotechnology, Instituto Nacional de Investigación y Tecnología Agraria y Alimentaria (INIA-CSIC), Madrid 28040, Spain; ⊥ CIBERNED, Instituto Salud Carlos III, Madrid 28029, Spain; # CIBERINFEC, Instituto Salud Carlos III, Madrid 28029, Spain; ∇ School of Medicine, Universidad Complutense de Madrid, Madrid 28040, Spain

## Abstract

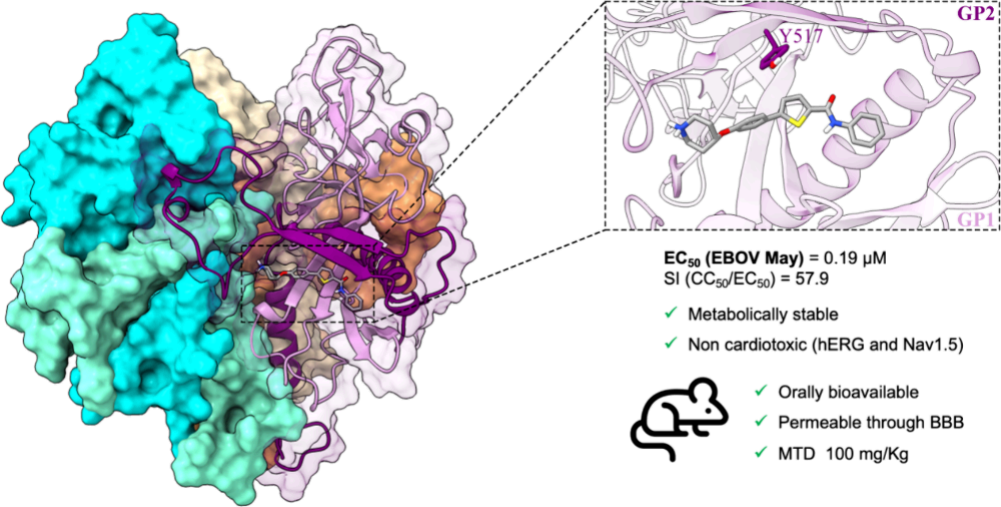

The endemic nature of the Ebola virus disease in Africa underscores the need for prophylactic and therapeutic drugs that are affordable and easy to administer. Through a phenotypic screening employing viral pseudotypes and our in-house chemical library, we identified a promising hit featuring a thiophene scaffold, exhibiting antiviral activity in the micromolar range. Following up on this thiophene hit, a new series of compounds that retain the five-membered heterocyclic scaffold while modifying several substituents was synthesized. Initial screening using a pseudotype viral system and validation assays employing authentic Ebola virus demonstrated the potential of this new chemical class as viral entry inhibitors. Subsequent investigations elucidated the mechanism of action through site-directed mutagenesis. Furthermore, we conducted studies to assess the pharmacokinetic profile of selected compounds to confirm its pharmacological and therapeutic potential.

## Introduction

Ebola virus disease (EVD) was first reported in Sudan and Zaire in 1976 as outbreaks of a hemorrhagic fever with mortality rates of 53 and 90%, respectively.^1^ These severe diseases were caused by Sudan virus (SUDV) and Ebola virus (EBOV), which belong to the *Filoviridae* family of RNA viruses. Within the *Ebolavirus* genus, six different species can be distinguished, *Orthoebolavirus zairense* (EBOV), *Orthoebolavirus sudanense* (SUDV), *Orthoebolavirus taieense* (TAFV), *Orthoebolavirus bombaliense* (BOMV), *Orthoebolavirus bundibugyense* (BUDV), and *Orthoebolavirus restoniense* (RESTV) where EBOV and SUDV are the most pathogenic strains.^2,3^ Major international concern about EBOV emerged as a result of the large epidemic in Sierra Leone, Guinea, and Liberia in 2013–2016 with more than 11,000 deaths and extends to the present due to the constant resurgence of new outbreaks in central and western Africa,^4,5^ including the last outbreak caused by SUDV in Uganda declared over on 11 January 2023.^6^

The endemic nature of the EVD in Africa underscores the need for prophylactic and therapeutic drugs that are affordable and easy to administer.^7,8^

EBOV is composed of different viral proteins: a nucleoprotein (NP), which is essential for viral replication, the same as viral protein 35 (VP35), viral protein 24 (VP24), and L protein (RNA-dependent polymerase), which are involved in the formation of the ribonucleoprotein complex with viral RNA; VP40, essential for viral assembly and budding; VP30 involved in RNA transcription and the EBOV glycoprotein (GP_1,2_), formed by subunits GP1 and GP2, located around the viral surface and responsible for host cell attachment and viral genome release into the cytoplasm. Additionally, there are two soluble glycoproteins (sGP and ssGP) that are released from infected cells.^9^ Ebola virus enters the cell via endocytosis, mainly via macropinocytosis and clathrin-mediated endocytosis, and has a high preference for macrophages and dendritic cells at early stages postinfection.^10^ EBOV travels through the endolysosomal pathway where the cysteine proteases cathepsins B and L cleave GP into its fusogenic form (GPcl), removing its glycan cap and exposing the receptor binding domain. This cleaved form of GP is then ready for interaction with Niemann-Pick C1 receptor (NPC1), a cholesterol transporter located in late endosomes, which is crucial for viral genome releasing to the cytoplasm and therefore for transcription and replication to occur.^11,12^

**Figure 1:**
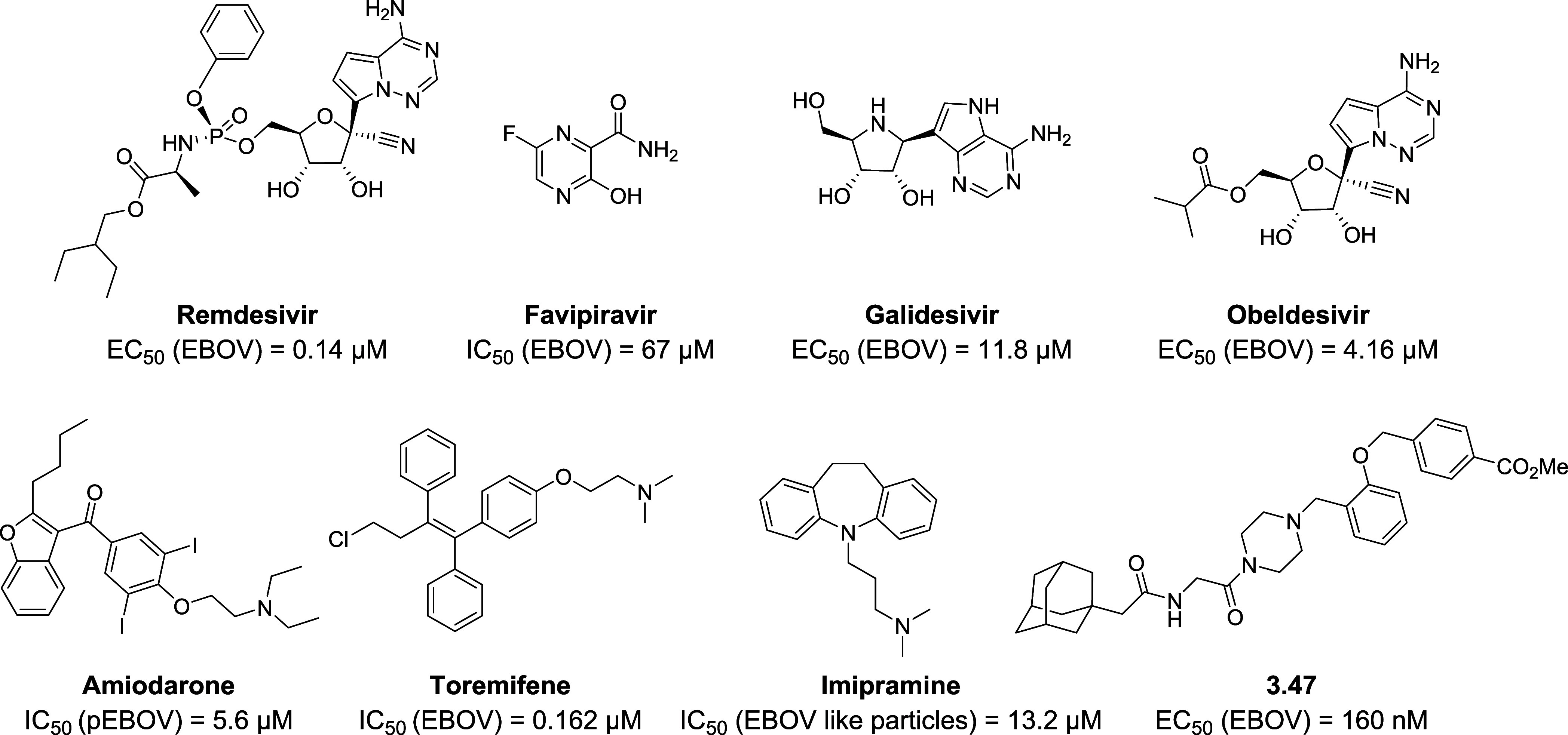
Chemical structure and antiviral activity of some reported anti-EBOV compounds.^8,20,23,25,27−30^

Currently, there are three approved preventive vaccines and two intravenous treatments for EVD based on monoclonal antibodies targeting different epitopes of EBOV-GP.^13−15^ Regarding small molecules, two RNA polymerase inhibitors, remdesivir and favipiravir, reached clinical trials but showed no signs of efficacy in EVD patients.^16,17^ Two other nucleoside analogs acting at the same level, galidesivir and obeldesivir, have successfully completed Phase I clinical trials in healthy volunteers but are yet to be evaluated in patients with EVD.^18,19^ Notably, obeldesivir can be orally administered and has demonstrated activity in nonhuman primates infected with SUDV^8^ (Figure 1).

In terms of drug repurposing, many efforts have been done to reuse approved drugs to combat EBOV.^20^ For example, amiodarone, an ion channel inhibitor with *in vitro* activity against EBOV, was used as compassionate therapy during the Ebola outbreak in Sierra Leone, although its therapeutic effect was inconclusive.^21^ Other approved drugs with anti-EBOV activity include toremifene and imipramine (Figure 1). These two compounds block infection by binding to EBOV-GP, acting as antiviral entry inhibitors.^22,23^ Viral entry inhibition by disrupting the NPC1/EBOV-GP interaction has been observed with several adamantane derivatives, including compound 3.47 and its variants, which act in an NPC1-dependent manner^12,24−26^ (Figure 1). Despite these efforts, no small molecule-based antiviral has yet been approved for the treatment of EVD.

In this context, and in view of the need of effective and affordable drugs to treat EVD, our main objective in this work is to develop a medicinal chemistry program around this challenge in order to propose some drug candidates. We focus on searching viral entry inhibitors, as these compounds could be combined with RNA polymerase inhibitors in a future therapy.

**Figure 2:**
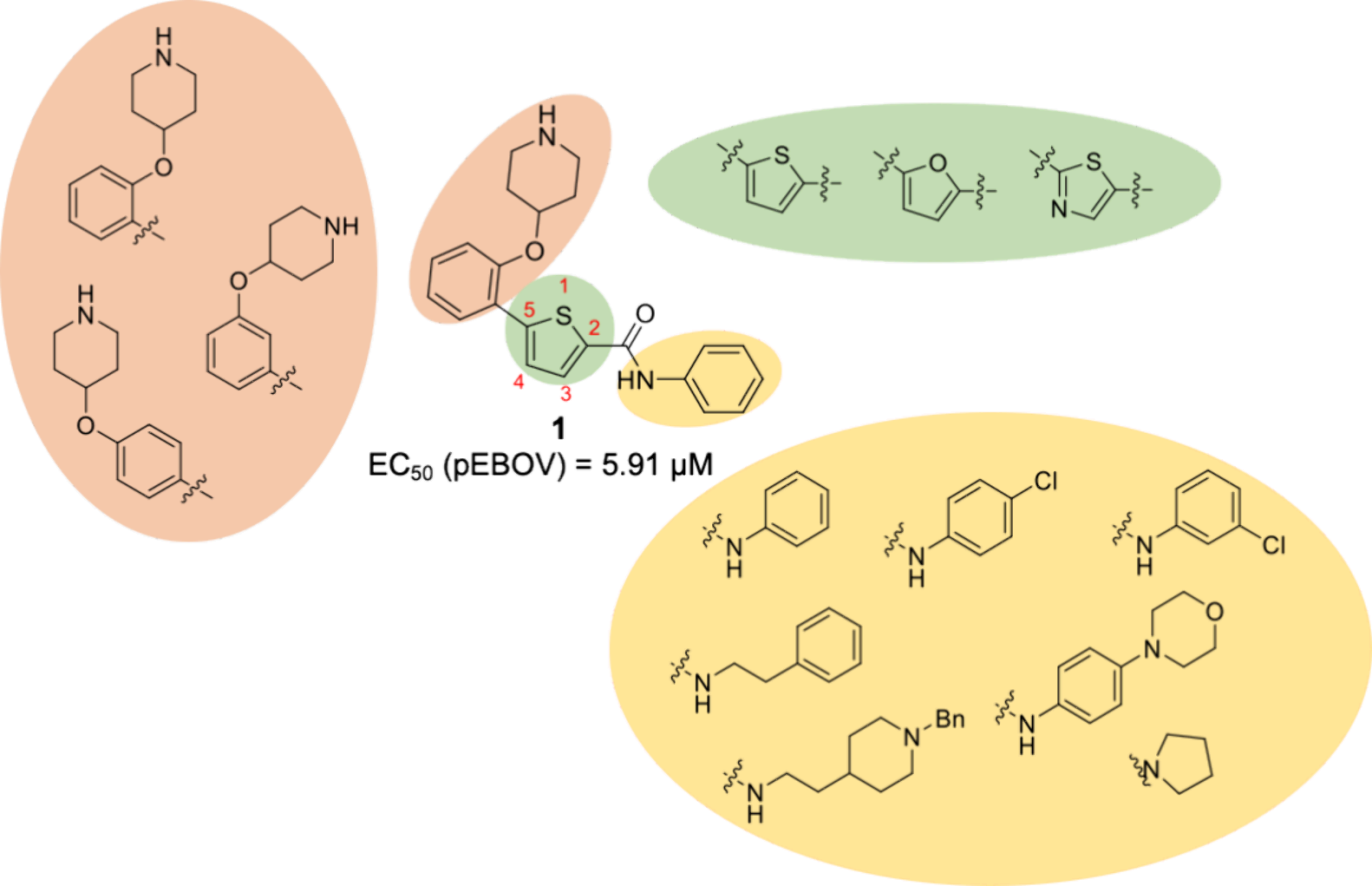
Chemical structure of hit **1** and structural modifications conducted in this study focused on varying the substituents at positions 2 and 5 of the thiophene ring, as well as replacing the thiophene ring with other five-membered rings.

Following a phenotypic screening using viral pseudotypes and our in-house chemical library,^31^ we identified a promising small molecule hit **1** with activity in the micromolar range (EC_50_ = 5.91 μM) against EBOV-GP-pseudotypes (pEBOV) (Figure 2). Hit heterocyclic scaffold **1** consist of a 2,5-disubstituted thiophene ring with an anilide group at position 2 and at position 5, a phenyl ring bearing an oxy-piperidine substituent at *ortho* position.

Herein, we report the development of a novel series of compounds derived from this initial hit, preserving the five-membered heterocyclic scaffold while modifying different substituents in order to optimize biological potency and drug-like properties. For the initial screening, a pseudotype viral system was employed, followed by validation assays using replicative EBOV. Subsequent investigations to unravel the mechanism of action of the identified viral entry inhibitors were carried out through site-directed mutagenesis. Furthermore, the pharmacokinetic profile of selected compounds was studied both *in vitro* and *in vivo*.

## Results and Discussion

### Chemistry

**Scheme 1:**
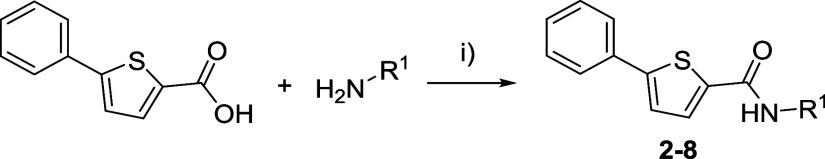
Synthesis of Amides **2**–**8** Reagents and conditions: (i) EDCl, HOBt, Et_3_N, CH_2_Cl_2_, 0 °C to rt, 24 h.

In order to improve the antiviral activity of hit **1** (Figure 2), several modifications were performed around the original scaffold. The hit consists of a thiophene ring with two substituents at positions 2 and 5, an anilide group at position 2 and a phenyl ring at position 5 bearing a piperidine linked by an oxygen atom at *ortho* position. First modifications consisted of maintaining the thiophene heterocycle substituted with a phenyl ring in 5 and changing the substituents of the amide group in order to check if biological activity was preserved by removing the oxy-piperidine ring. The length and nature of the *N*-substituents of the amide are variable, including phenyl, chlorophenyl, phenethyl, morpholinophenyl, 2-(1-benzylpiperidin-4-yl)ethyl, and pyrrolidin-1-yl. These modifications resulted in compounds **2–8**, which were synthetized through an amidation reaction of the commercial 5-phenylthiophene-2-carboxilic acid and the corresponding amine employing 1-ethyl-3-(3-dimethylaminopropyl)carbodiimide hydrochloride (EDCl) as carboxyl activating agent and 1-hydroxybenzotriazole (HOBt) to improve the efficiency of amine coupling and avoid side reactions. Triethylamine was used as a base and dichloromethane as solvent following a described reaction with modifications^32^ (Scheme 1 and Table 1).

**Table 1:**
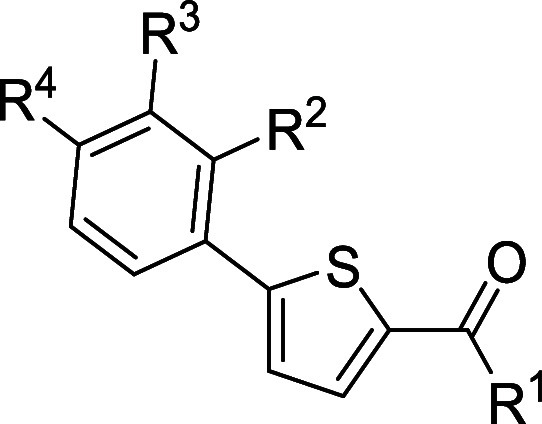

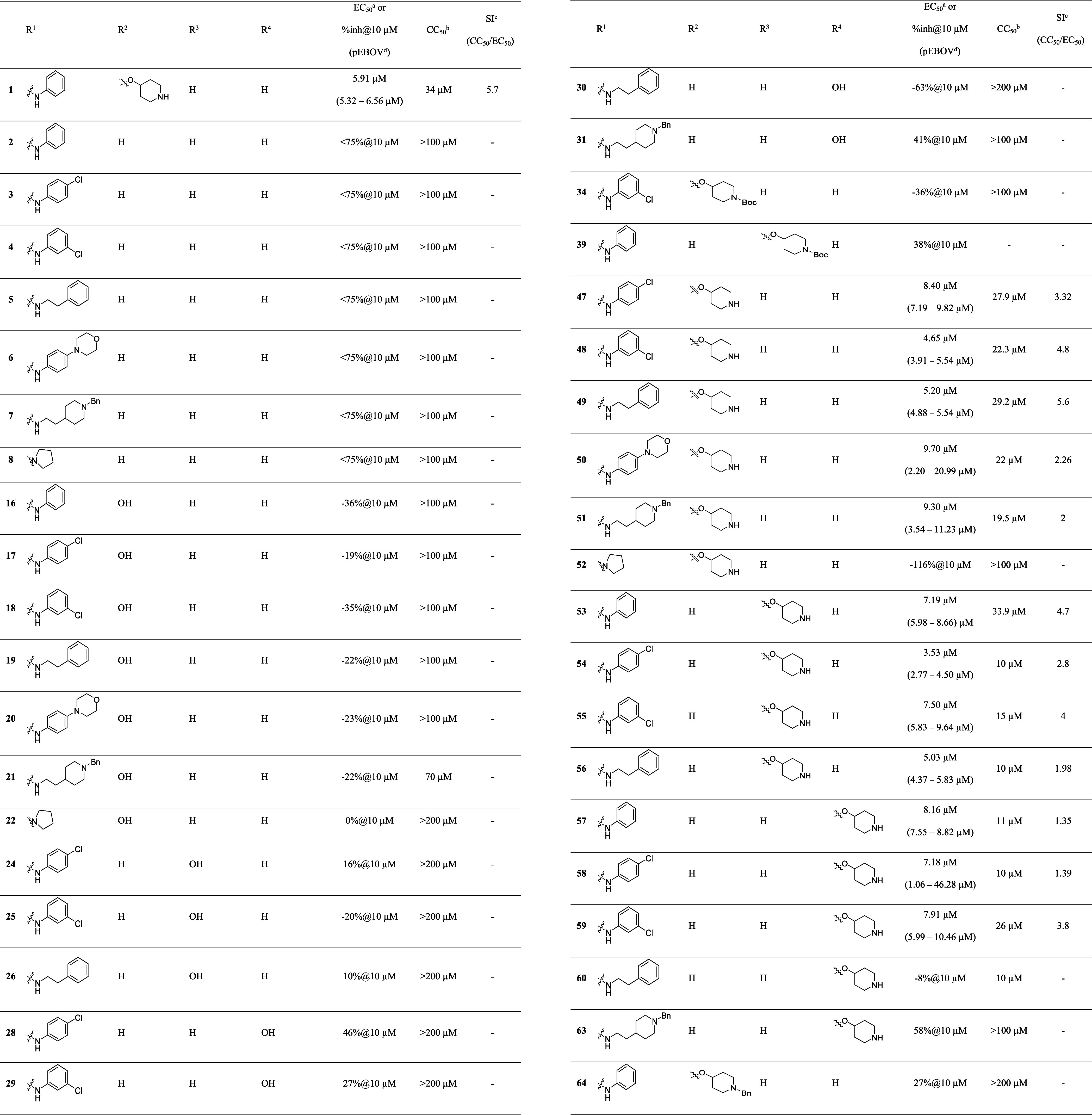
Antiviral Activity of the Thiophene Derivatives against EBOV-GP-Pseudotyped Virus (pEBOV)

a EC_50_: 50% effective concentration (with 95% confidence intervals in parentheses).

b CC_50_: 50% cytotoxic concentration.

c SI (CC_50_/EC_50_): selectivity index.

d Toremifene was used as reference of the assay (EC_50_ = 0.07 ± 0.05 μM, CC_50_ = 16 μM, SI = 229).^36^

**Scheme 2:**
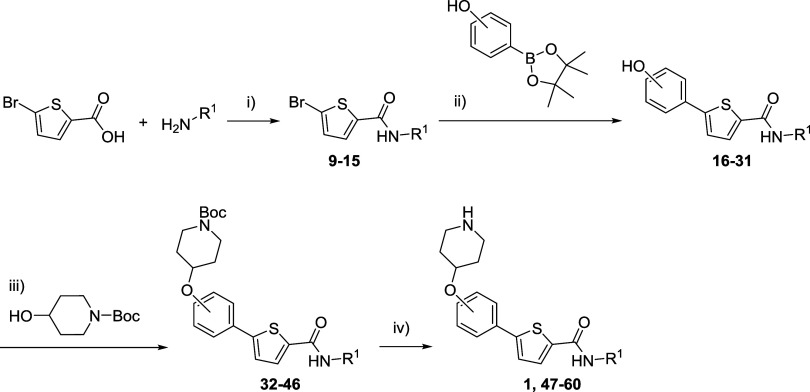
Synthesis of Thiophene Derivatives **1** and **47–60** Reagents and conditions: (i) EDCl, HOBt, Et_3_N, CH_2_Cl_2_, 0 °C to rt, 24 h; (ii) Pd(PPh_3_)_4_, Na_2_CO_3_, toluene/H_2_O/EtOH (2:1.5:1), 120 °C (MW), 20 min; (iii) DIAD, PPh_3_, THF, 0 °C to rt, 24 h; (iv) TFA, CH_2_Cl_2_, rt, 3 h.

The next chemical change was the addition of a piperidine linked by oxygen at *ortho*, *meta*, and *para* positions to the phenyl ring attached at thiophene position 5. Thus, the synthesis of the precursors with a hydroxyl group in the phenyl ring was needed. Starting with the 5-bromothiophene-2-carboxamides **9–15** prepared using the amide coupling procedure previously described, a Suzuki reaction was performed employing the corresponding hydroxyphenylboronic acid pinacol ester, palladium tetrakis(triphenylphosphine) as catalyst, and sodium carbonate as base following a modified methodology^33^ (Scheme 2). In order to obtain a biphasic medium that could solve all the reaction components, a mixture of toluene, water, and ethanol was selected. After several attempts at room temperature, it was decided to perform the reaction under microwave irradiation due to the kinetic improvement and easiness of handling,^34^ resulting in thiophene derivatives **16–31** with a hydroxy group at *ortho*, *meta*, and *para* positions of the phenyl ring (Scheme 2 and Table 1).

Final compounds with the piperidine moiety at *ortho*, *meta*, and *para* positions of the phenyl ring in 5 were obtained through a Mitsunobu reaction starting from compounds **16–31** previously obtained and the *N*-Boc-protected hydroxypiperidine. Diisopropyl azodicarboxylate (DIAD) was selected instead of diethyl azodicarboxylate (DEAD) due to the improvement in yields when the first was used. The Boc-protected derivatives **32–46** were deprotected using TFA to yield final thiophene derivatives **47–60**, including hit **1** (Scheme 2 and Table 1).

**Scheme 3:**
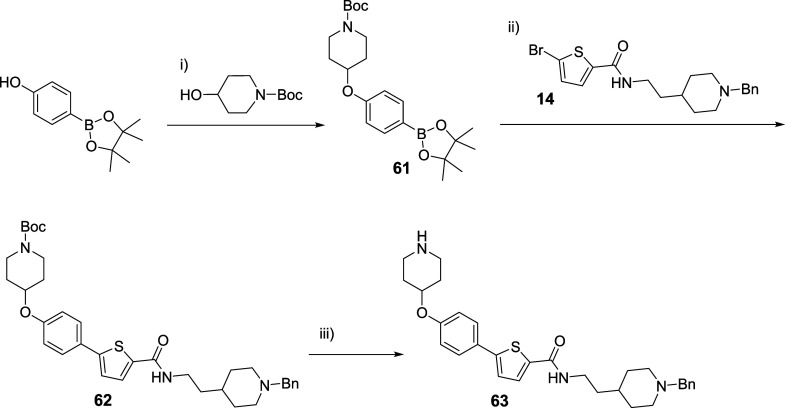
Synthesis of Thiophene Derivative **63** Reagents and conditions: (i) DIAD, PPh_3_, THF, 0 °C to rt, 24 h; (ii) Pd(PPh_3_)_4_, Na_2_CO_3_, toluene/H_2_O/EtOH (2:1.5:1), 120 °C (MW), 20 min; (iii) TFA, CH_2_Cl_2_, rt, 3 h.

To obtain thiophene **63** having a 2-(1-benzylpiperidin-4-yl)ethyl as substituent of the amide group and the piperidine moiety at *para* position of the phenyl ring at position 5, an alternative protocol was developed because the initial procedure described in Scheme 2 was unsuccessful. In that case, the phenylboronic acid pinacol ester with the Boc-protected piperidine at *para* position (**61**) was obtained employing a Mitsunobu reaction. Thus, starting from the brominated derivative **14** and employing the Suzuki coupling with the boronic pinacol ester **61** previously synthesized, it was possible to obtain the final *para*-substituted derivative **63** (Scheme 3).

**Scheme 4:**
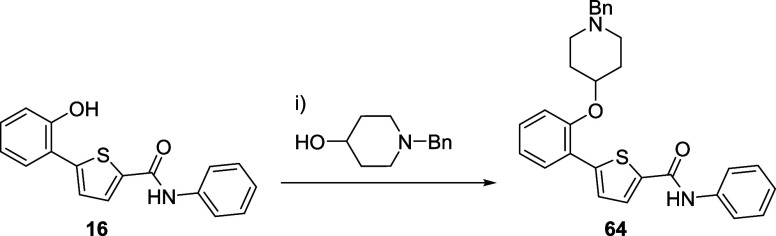
Synthesis of *N*-Benzyl-Protected Derivative **64** Reagents and conditions: (i) DIAD, PPh_3_, THF, 0 °C to rt, 24 h.

The influence of free amino group in the piperidine moiety was explored to determine its importance for biological activity. In addition to the evaluation of some of the Boc-protected precursors, a benzyl group was used to protect the nitrogen of piperidine employing again a Mitsunobu reaction. The *N*-benzyl-protected compound **64** was obtained following this procedure (Scheme 4).

**Scheme 5:**
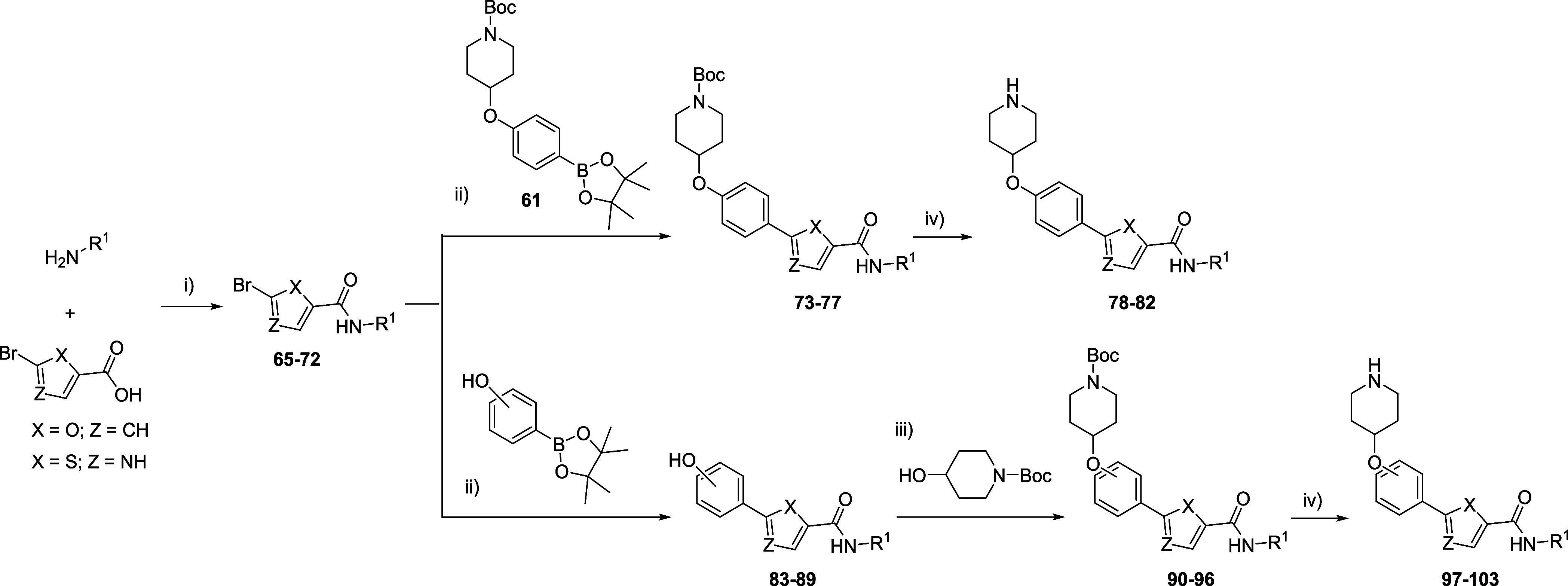
Synthesis of Furan and Thiazole Derivatives **78**–**82** and **97**–**103** Reagents and conditions: (i) EDCl, HOBt, Et_3_N, CH_2_Cl_2_, 0 °C to rt, 24 h; (ii) Pd(PPh_3_)_4_, Na_2_CO_3_, toluene/H_2_O/EtOH (2:1.5:1), 120 °C (MW), 20 min; (iii) DIAD, PPh_3_, THF, 0 °C to rt, 24 h; (iv) TFA, CH_2_Cl_2_, rt, 3 h.

Finally, to explore the impact of the thiophene ring in biological activity, some furan and thiazole derivatives were obtained as analogues of the most promising compounds following procedures described previously in Schemes 2 and 3. The *para*-substituted compounds (**78–82**) were achieved in shorter times and with good yields using the optimized procedure developed to synthesize thiophene **63**. In this case, the use of boronic pinacol ester **61** as a common intermediate enables their synthesis in larger quantities, facilitating the Suzuki coupling and reducing in one step the synthetic procedure. For *ortho* and *meta* derivatives, the standard protocol was employed to obtain **97–103** (Scheme 5 and Table 2).

**Table 2:**
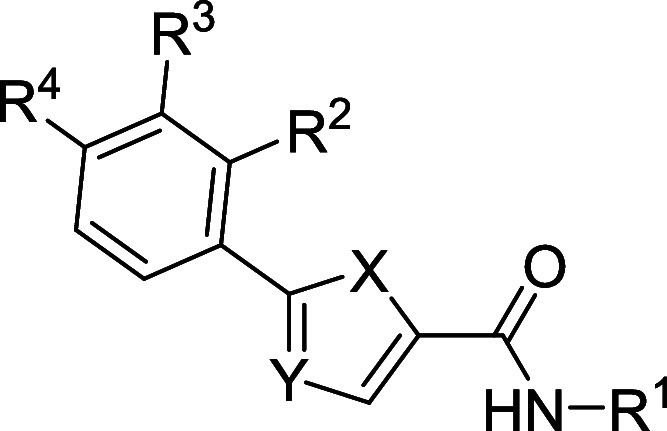

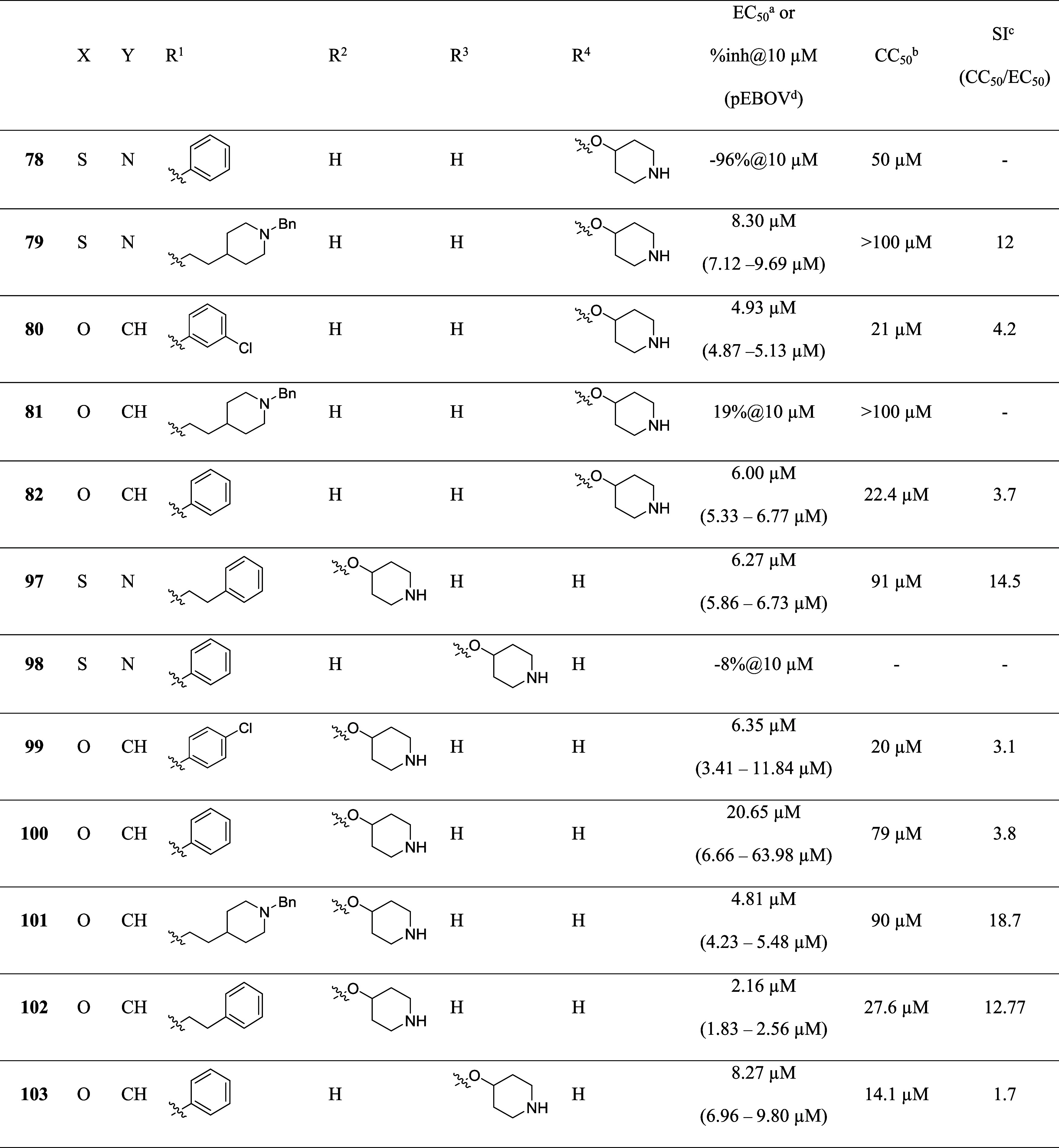
Antiviral Activity of the Thiazole and Furan Derivatives against EBOV-GP-Pseudotyped Virus (pEBOV)

a EC_50_: 50% effective concentration (with 95% confidence intervals in parentheses).

b CC_50_: 50% cytotoxic concentration.

c SI (CC_50_/EC_50_): selectivity index.

d Toremifene was used as reference of the assay (EC_50_ = 0.07 ± 0.05 μM, CC_50_ = 16 μM, SI = 229).^36^

### Primary Screening against Pseudotyped Viruses

After the synthesis of a number of derivatives based on hit **1**, they were evaluated as antivirals in VeroE6 cells infected with pEBOV. In particular, a model employing viral pseudotypes of human vesicular stomatitis virus (VSV) expressing EBOV-GP on their surface was used to initially assess the antiviral activity. Compounds that inhibited virus infection by more than 75% at 10 μM were further analyzed for potency, selectivity, and cytotoxicity. EC_50_ (50% effective concentration) and CC_50_ (50% cytotoxic concentration) were calculated subsequently to determine the corresponding selectivity index (SI) (Table 1). Employing pseudotyped viruses enabled us to conduct our experiments within in BSL-2 facilities instead of the BSL-4 required when working with native EBOV. This conferred a significant advantage in handling highly pathogenic viruses throughout the process.^35^

Initially, our focus was on probing the impact of the oxy-piperidine group on antiviral activity. To this end, compounds with only a phenyl ring attached to the 5 position of the thiophene were synthesized. Furthermore, modifications were made to the amide residue while maintaining the original heterocycle ring, aiming to assess the influence of substituents with different chemical properties on biological activity. Evaluation of the corresponding thiophenes, **2–8**, showed that removal of the oxy-piperidine group resulted in the loss of antiviral activity against pEBOV, regardless of the amide substituent. This underscores the essential nature of the piperidine residue for sustaining activity.

Further modifications were undertaken to explore the impact of the position of the oxy-piperidine group, whether at the *ortho*, *meta*, or *para* position of the phenyl ring attached to the 5 position. It is noteworthy that hydroxy intermediates isolated and characterized during the synthetic procedures, **16–26** and **28–31**, were also evaluated. However, none of these intermediates exhibited antiviral activity, thus affirming the critical role of the piperidine residue in maintaining the compound’s efficacy.

Among compounds with an oxy-piperidine group in *ortho*, *meta,* and *para* positions of the phenyl ring attached to the 5 position (**1**, **47–60**, and **63**), along with different substituents of the amide group (phenyl, chlorophenyl, phenethyl, morpholinophenyl, 2-(1-benzylpiperidin-4-yl)ethyl, and pyrrolidin-1-yl), almost all of them were active against pEBOV within the same range as hit **1** (EC_50_ range: 3.53–9.70 μM). Notably, only thiophenes **52**, **60**, and **63** were completely inactive.

In order to study the impact of the free amine in the piperidine moiety, a couple of protected Boc precursors (**34** and **39**) were evaluated, resulting in a complete loss of activity. Additionally, when this amine was substituted with a benzyl group in thiophene **64**, the compound again lacked activity, underscoring the significance of the free amine group in the piperidine for antiviral activity.

Furthermore, several thiazole (**78–79** and **97–98**) and furan (**80–82** and **99–103**) analogs were synthesized and evaluated, mimicking the most promising thiophene derivatives tested (Table 2). Biological activity seems to be independent of the aromatic heterocycle and was maintained in compounds with thiazole or furan.

In broad terms, compounds with a free amine on the piperidine ring, regardless of its position on the phenyl ring and the nature of the five-membered heterocycle, maintain antiviral activity. In contrast, those with just a phenyl ring at position 5, an hydroxyphenyl, or an *N*-protected oxy-piperidine were inactive.

Regarding selectivity, viral pseudotypes with the vesicular stomatitis virus envelope GP (VSV-G) were used as control. None of the final compounds showed antiviral activity in this system, highlighting their specificity for targeting EBOV-GP (Table S1 of the Supporting Information).

### Confirmation Screening against Infectious EBOV

Due to the promising activities found for the thiophene derivatives as viral entry inhibitors, a selection of compounds evaluated in pseudotypes were further tested using VeroE6 cells infected with the wild-type Zaire EBOV Mayinga 1976 strain (EBOV May). Alongside with active derivatives, one inactive compound was included in the assay for comparative purposes (Table 3). Noteworthily, we found a strong correlation between antiviral activity in the surrogate model and infectious EBOV combined with good selectivity indexes. This outcome validates our approach in rapidly selecting EBOV entry inhibitors.

**Table 3:** Antiviral Activity of Selected Compounds against Replicative EBOV

	EC_50_^a^ (EBOV May^d^)	CC_50_^b^	SI^c^ (CC_50_/EC_50_)
**1**	1.50 μM	34 μM	22.6
	(1.18–1.82 μM)		
**5**	>100 μM	100 μM	
**47**	1.55 μM	27.9 μM	18
	(1.22–1.91 μM)		
**48**	1.30 μM	22.3 μM	17.1
	(0.99–1.71 μM)		
**49**	0.79 μM	29.2 μM	36.9
	(0.58–1.00 μM)		
**50**	7.74 μM	22 μM	2.8
	(4.58–13.06 μM)		
**51**	2.96 μM	19.5 μM	6.6
	(2.29–3.81 μM)		
**53**	0.30 μM	33.9 μM	113
	(0.08–0.84 μM)		
**54**	1.12 μM	10 μM	8.9
	(1.05–1.20 μM)		
**55**	1.86 μM	15 μM	8.0
	(2.99–1.15 μM)		
**56**	4.93 μM	10 μM	2.0
	(3.92–6.19 μM)		
**57**	0.19 μM	11 μM	57.9
	(0.09–0.31 μM)		
**58**	2.42 μM	10 μM	4.13
	(1.97–2.96 μM)		
**59**	1.19 μM	26 μM	21.8
	(1.02–1.38 μM)		
**79**	7.83 μM	>100 μM	12.8
	(6.28–9.78 μM)		
**80**	2.91 μM	21 μM	7.2
	(2.55–3.31 μM)		
**82**	9.58 μM	22.4 μM	2.3
	(8.27–11.10 μM)		
**97**	15.68 μM	91 μM	5.8
	(14.31–17.17 μM)		
**99**	2.53 μM	20 μM	7.9
	(1.94–3.29 μM)		
**101**	9.50 μM	90 μM	9.5
	(8.52–10.61 μM)		
**102**	6.19 μM	27.6 μM	4.5
	(5.44–7.05 μM)		
**103**	9.26 μM	14.1 μM	1.5
	(8.68–9.87 μM)		

a EC_50_: 50% effective concentration (with 95% confidence intervals in parentheses).

b CC_50_: 50% cytotoxic concentration.

c SI (CC_50_/EC_50_): selectivity index.

d Favipiravir was used as reference of the assay (EC_50_ = 67 μM (95% CI = 56–75 μM), CC_50_ > 1000 μM, SI = 14.9).^28^

Similar to the results obtained from the pseudovirus assay, we observed that thiophenes with the oxy-piperidine ring at the *ortho*, *meta*, and *para* positions exhibit similar activity levels against infectious EBOV in the micromolar range, being the same behavior present in thiazole and furan derivatives. However, it is noteworthy that there was a slight enhancement in activity against wild-type EBOV compared to pseudotypes that led to better selectivity indexes. This is not surprising because both infectious models are different. Nevertheless, it looks like thiophenes have better selectivity indexes than thiazole and furan derivatives (e.g., thiophene **49** vs thiazole **97** and furan **102**, thiophene **53***vs* furan **103**, thiophene **57***vs* furan **82**, and thiophene **59***vs* furan **80**). Additionally, among thiophenes, those lacking substituents in the phenyl ring attached to the amide showed a generally improved therapeutic window (e.g., **1**, **49**, **53**, and **57**).

Taking these findings into account, the two best compounds in terms of antiviral activity and selectivity index, thiophene derivatives **53** and **57**, together with hit **1** were selected for further investigation to explore the potential of this new class of EBOV entry inhibitors for subsequent development as antiviral drugs.

Additionally, to check the usefulness of these inhibitors to treat infections by other ebolaviruses, these three compounds were tested in pseudotypes with the SUDV envelope, showing activities slightly better than those in pEBOV: EC_50_ = 2.64 μM (95% CI = 1.66–4.19 μM), 3.05 μM (95% CI = 2.52–3.69 μM), and 1.68 μM (95% CI = 1.32–2.14 μM) for thiophenes **1**, **53**, and **57**, respectively.

### Deciphering the Mechanism of Action of Thiophene Derivatives

**Figure 3:**
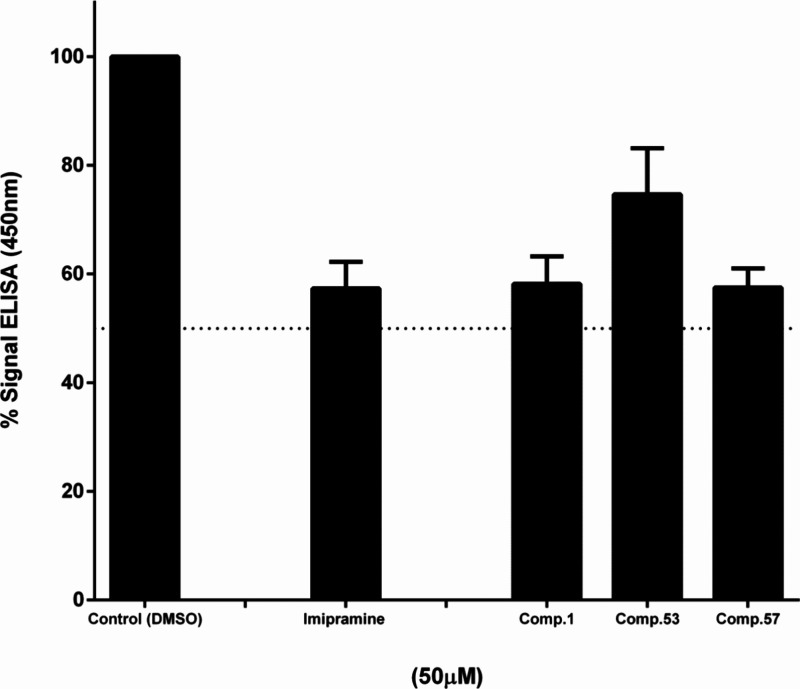
Assessment of the inhibitory effect of selected thiophenes on the interaction between EBOV-GPcl/NPC1-domain C by ELISA. ELISA plates, coated with cleaved EBOV particles, were subjected to incubation with hNPC1-domain C-flag in the presence or absence (control) of compounds **1**, **53**, and **57**, as well as positive control (imipramine). The bound domain C was subsequently detected utilizing an antiflag antibody conjugated to horseradish peroxidase, followed by Ultra-TMB substrate. Error bars represent the standard deviation from three independent experiments.

Considering the antiviral activity showed for this class of compounds in viral pseudotypes of VSV expressing on their surface EBOV-GP, this family acts at the viral entry level. In order to go deeper into the mechanism of action, a potential inhibition of the NPC1/EBOV-GP interaction was investigated due to its key role in viral entry.^37^ With this aim, we carried out an enzyme-linked immunosorbent assay (ELISA)-based assay previously used in our laboratory^38^ to check if thiophene derivatives have any effect in the binding of EBOV-GP to NPC1 (Figure 3). Imipramine was used as positive control of the assay.^39^ As shown in Figure 3, thiophenes **1** and **57** exhibit a comparable effect than the control imipramine, whereas derivative **53** does it to a lesser extent.

**Figure 4:**
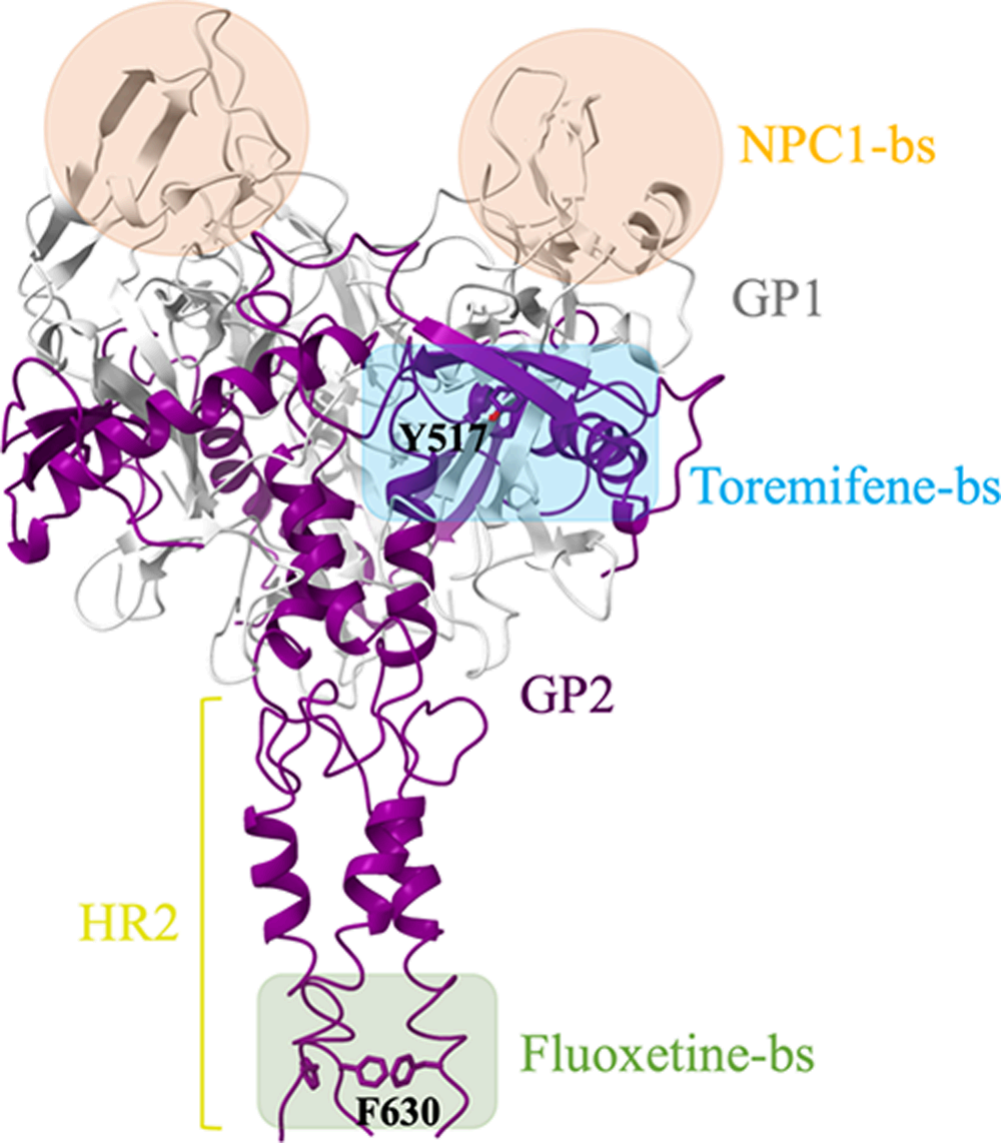
Side view of the homotrimeric EBOV-GPcl (PDB ID: 5JQ7). NPC1, toremifene, and fluoxetine-binding sites (bs) are indicated by highlighted squares in orange, cyan, and green, respectively.

Imipramine is reported to bind in a cavity between the two subunits of the EBOV-GP, GP1 and GP2.^23^ Acting at this level disrupts the prefusion conformation, leading to an allosteric inhibition of the NPC1/EBOV-GP interaction. This binding pocket at the GP1/GP2 interface was first described after the cocrystallization of EBOV-GP with toremifene and Y517 was identified to be a critical residue for the drug interaction.^22^ In fact, mutation of this residue to a serine (Y517S) results in a drastic loss of potency of toremifene.^40^ Apart from the fusion loop of GP, another drug binding site is located in the HR (heptad repeat region 2) region of EBOV-GP at the base of the trimer. This site was identified by mutational analysis. Using F630H and F630W, the binding site of fluoxetine at the HR domain was identified^41^ (Figure 4).

**Figure 5:**
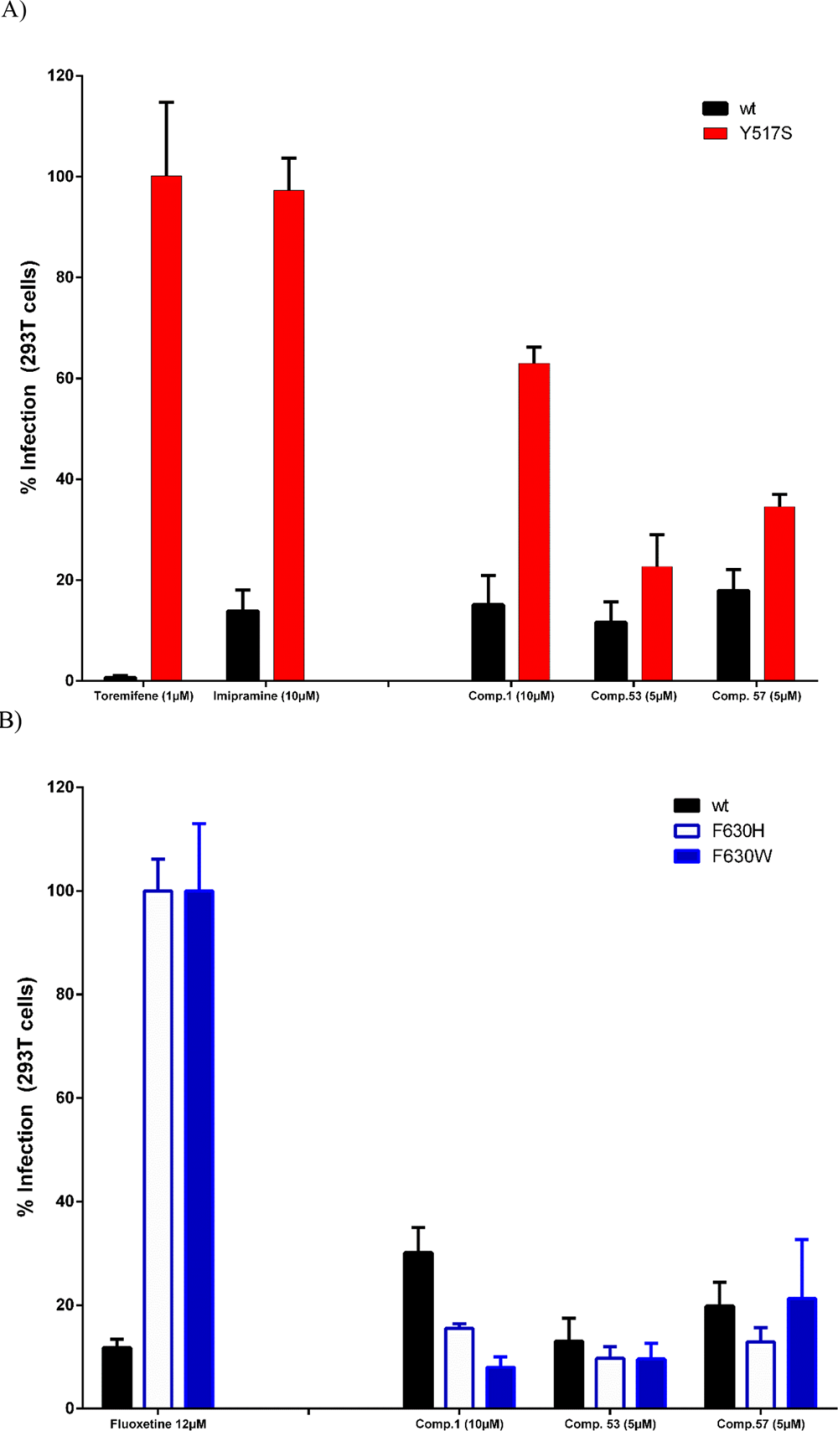
Antiviral evaluation of thiophenes **1** (10 μM), **53** (5 μM), and **57** (5 μM) against EBOV-GP Y517S (A) and EBOV-GP F630H and EBOV-GP F630W (B) mutant-pseudotyped virus in 293T cells infected with either EBOV wt or mutant-pseudotyped lentiviral particles. Following a 48 h incubation period, cells were lysed and examined for luciferase expression. The percentage of infection is depicted as 100% with toremifene for Y517S mutant (A) or with fluoxetine for F630H/W mutants (B). The concentration of tested compounds was selected based on the corresponding CC_50_. Error bars represent the standard deviation from three independent experiments.

Giving this, to unravel how thiophene derivatives interfere with the NPC1/EBOV-GP interaction, we tested them using EBOV pseudotypes carrying mutation in different residues of EBOV-GP, such as Y517S, F630H, and F630W. These mutants were proposed as standard screening tools for classifying small molecule hits against EBOV viral entry.^41^ As control of the assays, toremifene and imipramine were used to verify the lack of activity in pEBOV GP Y517S with respect to pEBOV wt GP (Figure 5A). Meanwhile, for pEBOV GP F630H and F630W, fluoxetine was used as reference (Figure 5B).

After testing thiophenes **1**, **53**, and **57** in pEBOV GP Y517S, F630H, and F630W, we observed that while the three compounds showed a loss of antiviral activity in Y517S mutants, remaining antiviral activity was observed in both F630 mutants (Figure 5A and 5B, respectively). These results pointed to the fact that antiviral thiophenes act at the level of Y517 within the fusion loop region between GP1 and GP2 at the same site targeted by toremifene.

### Computational Studies

The binding of inhibitors to the internal fusion loop region of the EBOV-GP is identified as one of the mechanisms that inhibits EBOV’s entry into the host cell.^22^ The GP is composed of a homotrimer consisting of heterodimers, comprised of two subunits: GP1 and GP2. GP1 facilitates the initial attachment to the cell surface, while GP2 mediated viral membrane fusion and the release of RNA into the cytoplasm.^42^ Therefore, small molecules capable of binding to the hydrophobic groove located at the interface between GP1 and GP2 could allosterically influence the binding of the GP complex to the NPC1 protein.

**Figure 6:**
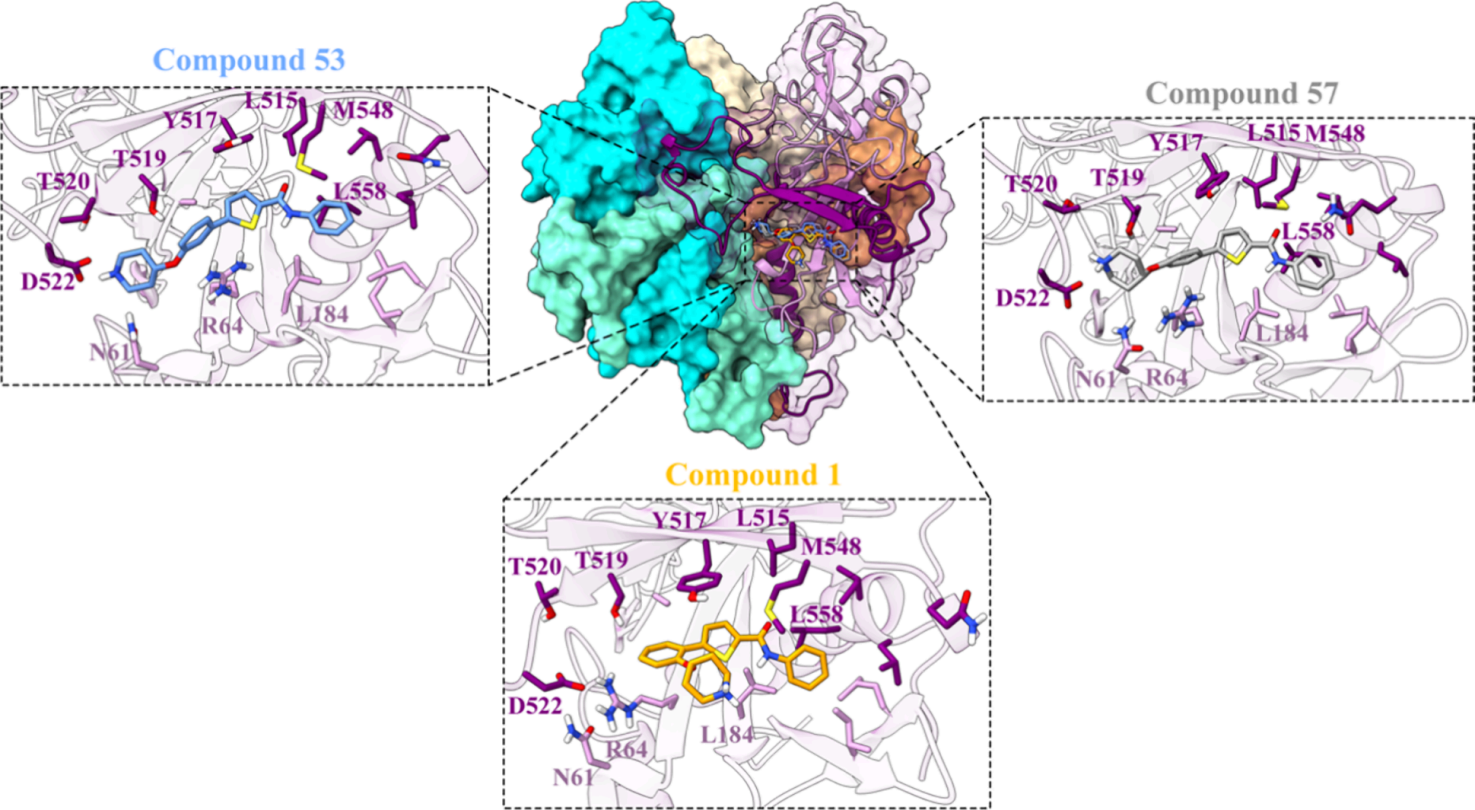
Representative binding mode of thiophenes **57**, **53**, and **1** (rendered in gray, blue, and orange sticks, respectively) within the cavity, formed between GP1 (illustrated as a plum-colored cartoon) and GP2 (depicted as a purple cartoon), as identified from 1.5 μs MD simulations. Detailed close-up of the ligand binding pocket, highlighting essential residues within a 5 Å proximity of the ligand. Other heterodimers of GP1-GP2 are depicted with a protein surface rendered in cyan, aquamarine, wheat, and sandy brown colors. The X-ray structure of the EBOV-GP in complex with toremifene (PDB ID: 5JQ7) was used as a starting point for molecular modeling studies.

To gain a comprehensive understanding of the binding mode and inhibition mechanism of thiophene derivatives and following the results of the mutagenesis studies performed, docking and molecular dynamics (MD) simulations have been employed. In this study, we utilized the crystal structure of the EBOV-GP in complex with toremifene (PDB ID: 5JQ7)^22^ for performing the docking of the three selected thiophenes, **1**, **53**, and **57**. The binding modes with the most favorable docking scores^43^ were further utilized for MD simulations. The docking study’s findings demonstrate that irrespective of the positioning of the oxy-piperidine substituent at the *ortho*, *meta*, or *para* position of the phenyl ring (compounds **1**, **53**, and **57**, respectively), the three thiophene derivatives could bind within the pocket situated at the interface of GP1 and GP2 (Figure 6). However, it is noteworthy that the docking scores for **53** and **57** are better than that of **1** (with scores of −7.2 *vs* −6.1).

The anilide group in the three cases is situated within the hydrophobic pocket in close proximity to the positions of M548 and L558 residues. Additionally, the thiophene ring forms a π–π stacking interaction with the residue Y517, a key interaction between inhibitors and the GP protein. This is supported by experimental data (Figure 5A), which show that the three compounds lose antiviral activity when tested against pseudotypes with the EBOV-GP containing the Y517S mutation. Moreover, the oxy-piperidine ring, independently of its position, protrudes into an area of the pocket containing polar or charged residues such as R64, N61, and D522 (Figure 6).

**Figure 7:**
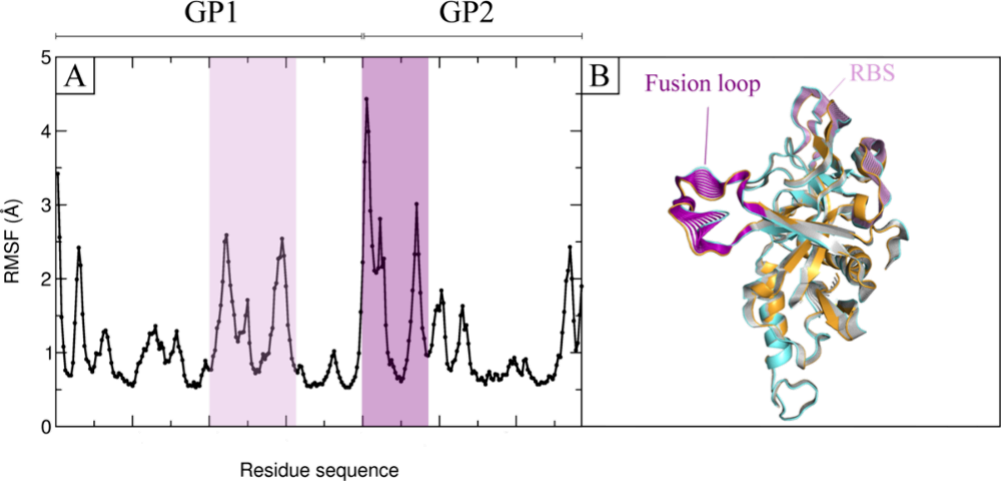
(A) RMSF (Å) for the average of the three independent replicas of **57** simulated systems. The highlighted regions correspond to the fusion loop (in purple) and receptor (NPC1) binding sites (in plum). (B) Essential dynamics (ED) analysis of the 500 ns MD simulations run for GP-**57** complex. Only the first essential motion of the C_α_ atoms is shown.

The stability of binding and the effect of the three selected thiophene derivatives binding on GP’s structural features were assessed by analyzing the positional root-mean-square deviation (RMSD) and root-mean-square fluctuation (RMSF) of the protein backbone during simulations. Evaluation of the RMSD for the protein backbone atoms (depicted by the black profile in Figure S1 from the Supporting Information) and the ligand confirmed the strong stability of **57** (violet profile in Figure S1A) and **53** (blue profile in Figure S1B) throughout the 500 ns MD simulations across three replicas. Conversely, ligand **1** (orange profile in Figure S1C) exhibited higher RMSD values, indicating comparatively lower stability within the binding site. These results align with the free energy of ligand binding to the protein as determined using the MM/GBSA method^44^ (Table S2 of the Supporting Information). The RMSF profiles for the three compounds show similar fluctuation patterns, with GP1 displaying the highest fluctuations in residues 110–120 near the receptor binding site, while GP2 exhibits significant fluctuations in residues 520–540 within the fusion loop^45^ (Figure 7A).

The impact of inhibitors on the dynamics of GP was further investigated through essential dynamics (ED)^46^ analysis. ED was employed to delineate how ligand binding influences the principal motions of the protein backbone. Based on ED results (Figure 7B), binding of the inhibitor **57** induces conformational changes in the GP complexes, promoting movements in the RBS region and fusion loop. These changes lead to increased conformational flexibility in these critical regions for the NPC1 binding, which play a significant role in the infectivity of the virus.

Analysis of hydrogen bonding and van der Waals interactions shows that both play crucial roles in the inhibition of thiophene derivatives. In the case of compound **57**, its N2 atom forms hydrogen bonds with residues T520 and D520 from GP2, as well as N61 and E100 from GP1 (Figure S2 from the Supporting Information). Compound **53** exhibits a similar pattern, although to a lesser extent compared to compound **57**, while compound **1** shows even weaker interactions (Figures S3 and S4 from the Supporting Information). The π–π stacking interaction with Y517, occurring at an average distance of 4.9 ± 0.5 Å for **57** and **53**, indicates stable interactions, whereas a weaker stacking interaction is observed for **1** at a mean distance of 6.1 ± 1.1 Å. These findings are consistent with experimental data and may explain the lower antiviral activity of compound **1** with respect to **53** and **57**.

Based on these findings, thiophene **57** was selected to characterize its drug-like properties due to the promising antiviral data (EC_50_ = 0.19 μM) (Table 3) and the inhibitory effect of the interaction between NPC1-domain C and EBOV-GPcl (Figure 3). Moreover, thiophene **1** was also characterized for comparative purposes.

### *In Vitro* Drug-like Profile

Two drug-like properties were selected to be studied *in vitro*, metabolic stability and cardiotoxicity, due to their crucial role in the future development of drug candidates.^47^

On the one hand, the metabolic stability of thiophene **57** was characterized according to half-life and intrinsic clearance parameters employing an *in vitro* incubation at 37 °C with mouse and human liver microsomes. Analysis of the results (Table 4) reveals significantly prolonged half-life values in both species compared to the reference drug, verapamil. On the other hand, the ionic channel inhibition induced by compounds **1** and **57** was measured in hERG and Nav1.5 channels in order to identify a potential cardiotoxic risk alert.^48^ Compounds having an IC_50_ < 10 μM are considered as cardiotoxic. As shown in Table 5, none of them have this risk in contrast to the positive controls astemizole for hERG and tetrodotoxin for Nav1.5, although thiophene **57** showed a higher IC_50_, around 50 μM (Table 5).

**Table 4:** *In Vitro* Microsomal Stability of Thiophene **57** in Liver Microsomes of Different Species

	Metabolic stability in human liver microsomes		Metabolic stability in mouse liver microsomes	
	*t*_1/2_ (min)	CL_int_^a^ (mL/min/mg protein)	*t*_1/2_ (min)	CL_int_^a^ (mL/min/mg protein)
**57**	130 ± 9	4.4 ± 0.3	29 ± 5	90 ± 10
Verapamil	22 ± 2	26 ± 3	10 ± 1	230 ± 30

a CL_int_, intrinsic clearance.

**Table 5:** Ionic Channel Inhibition Induced by the Tested Compounds, Thiophenes **1** and **57**, and Positive Controls

	IC_50_^a^ (hERG)	IC_50_^a^ (Nav1.5)
**1**	10.60 μM	20.4 μM
**57**	48.9 μM	>50 μM
Astemizole	0.23 μM	
Tetrodotoxin		4.11 μM

a IC_50_: 50% inhibitory concentration.

### *In Vivo* Pharmacokinetic Study

In order to investigate the plasma pharmacokinetic and brain distribution of selected thiophenes **1** and **57**, a study in male BALB/c mice was carried out. These animals received a single dose of 10 mg/kg intraperitoneally (i.p.) and 50 mg/kg orally (p.o.). Peak plasma concentrations were observed at 0.25 and 1.00 h for thiophene **1** and at 0.50 and 2.00 h for thiophene **57**, respectively. Both compounds displayed rapid absorption with thiophene **1**, exhibiting a faster absorption rate. In terms of brain penetration, both compounds showed greater brain exposures compared to plasma exposure, as evidenced by brain/plasma ratio (*K*_p_) values > 1 (Tables 6 and 7).

**Table 6:** Pharmacokinetic Parameters of Thiophene **1** after Single Intraperitoneal (i.p.) Administration at 10 mg/kg and Oral Administration (p.o.) at 50 mg/kg in Male BALB/c Mice

**Matrix**	**Route**	**Dose (mg/kg)**	***T***_**max**_^a^**(h)**	***C***_**max**_^b^**(ng/mL)**	**AUC**_**last**_^c^(h*ng/mL)	*t*_1/2_ (h)	**Brain-*K*_p_**^d^**(*C***_**max**_**)**	**Brain-*K*_p_**^d^**(AUC**_**last**_**)**
Plasma	i.p.	10	0.25	121.75	271.74	1.96		
	p.o.	50	1.00	112.31	541.78	5.60		
Brain	i.p.	10	2.00	124.80	600.09		1.03	2.21
	p.o.	50	6.00	316.70	3341.42		2.82	6.17

a *T*_max_: time to reach *C*_max_.

b *C*_max_: peak serum concentration.

c AUC_last_: area under the plasma concentration–time curve from time zero to the time of the last quantifiable concentration.

d *K*_p_: brain/plasma ratio.

**Table 7:** Pharmacokinetic Parameters of Thiophene **57** after Single Intraperitoneal (i.p.) Administration at 10 mg/kg and Oral (p.o.) Administration at 50 mg/kg in Male BALB/c Mice

**Matrix**	**Route**	**Dose (mg/kg)**	***T***_**max**_^a^**(h)**	***C***_**max**_^b^**(ng/mL)**	**AUC**_**last**_^c^(h*ng/mL)	*t*_1/2_ (h)	**Brain-*K*_p_**^d^**(*C***_**max**_**)**	**Brain-*K*_p_**^d^**(AUC**_**last**_**)**
Plasma	i.p.	10	0.50	1035.97	2973.11	5.18		
	p.o.	50	2.00	772.86	8610.24	8.76		
Brain	i.p.	10	4.00	1678.30	25429.34		1.62	8.55
	p.o.	50	4.00	5595.95	97086.13		7.24	11.28

a *T*_max_: time to reach *C*_max_.

b *C*_max_: peak serum concentration.

c AUC_last_: area under the plasma concentration–time curve from time zero to the time of the last quantifiable concentration.

d *K*_p_: brain/plasma ratio.

In terms of plasma levels, according to data from Tables 6 and 7, estimated concentrations of thiophenes **1** and **57** after i.p. (10 mg/kg) administration are 0.32 and 2.74 μM, respectively. Conversely, after p.o. (50 mg/kg) administration, plasma levels were 0.29 μM for **1** and 2.04 μM for **57**. Considering that the EC_50_ values for both compounds are 1.50 and 0.19 μM, respectively, in a forthcoming study to assess efficacy in an animal model of the disease, the dose for thiophene **1** should be increased, while the dose for thiophene **57** could be reduced.

In terms of brain levels, concentrations are 0.33 or 0.81 μM after i.p. or p.o. administration of thiophene **1**. For thiophene **57**, concentrations are significantly higher, being 4.43 or 14.80 μM after i.p. or p.o. administration, respectively.

Considering plasma and brain levels, thiophene **57** showed a clear advantage because effective dose could be reduced in a future efficacy *in vivo* study, reducing the possibility of adverse effects.

Moreover, both thiophenes demonstrate the remarkable ability to penetrate the blood–brain barrier. This is particularly notable given the neurological complications experienced by survivors and the limitations observed with therapeutic antibodies in addressing this issue.^49,50^

### Single-Dose Acute Tolerability Study

The objective of this study was to evaluate the major toxic effects and determine the maximum tolerated dose of thiophene **57** after single oral administration to male and female C57BL/6 mice, followed by 4 days postdose observations.

Administration of a single oral dose of **57** at 50 mg/kg to C57BL/6 mice was well tolerated. At 100 mg/kg dose, transient clinical signs were noted along with a slight decrease in percent body weight gain and feed consumption. However, test item revealed severe clinical signs, including mortality, in both male and female mice at 250 mg/kg.

Based on these findings, it is concluded that the maximum tolerated dose for single-dose oral administration of thiophene **57** to mice is 100 mg/kg.

## Conclusions

Here, we report the chemical optimization of our initial thiophene hit **1**. For this purpose, we synthesized several derivatives by changing the amide substituents and the position of the oxy-piperidine moiety in the phenyl ring and exploring different five-membered heterocyclic rings such as furan or thiazole. After an initial screening using pseudotyped viruses, the activity of selected compounds was confirmed in authentic EBOV. Our findings indicate that the oxy-piperidine attached to the phenyl ring is crucial for maintaining the activity of the new synthesized derivatives, regardless of whether the substituent is in the *ortho*, *para*, or *meta* position. The amide substituent and the nature of the aromatic heterocycle appear to play a minor role in the activity of the compounds compared to the influence of substitutions at position 2.

With respect to the mechanism of action of this new family of antiviral compounds, considering that these are viral entry inhibitors, which are able to disrupt the interaction between EBOV-GPcl and the virus entry receptor, NPC1, we focused on the hypothesis that this disruption may be due to the binding of the derivatives to a hydrophobic pocket between the two subunits of EBOV-GP, GP1 and GP2. This fact was experimentally confirmed by the loss of activity of selected thiophenes when tested against pseudotypes carrying the EBOV-GP envelope with the Y517S mutation, a key residue in this binding site. Additionally, computational studies confirmed the strongest stability of derivative **57** in this pocket and the influence of the binding on critical regions of EBOV-GP essential for effective binding to the NPC1 receptor.

Based on these findings, thiophene **57** was selected for a series of pharmacokinetic studies revealing good metabolic stability and the absence of cardiotoxic effects. Subsequently, an *in vivo* pharmacokinetic study conducted in mice demonstrated that **57** can be orally administered at doses of 50 mg/kg or lower, effectively crossing the blood–brain barrier, a notable advantage compared to existing treatments. Furthermore, with a maximum tolerated dose of 100 mg/kg, there exists a significant therapeutic window to mitigate potential side effects. Consequently, this derivative emerges as an ideal candidate for future *in vivo* efficacy trials, holding promise as a potential anti-Ebola therapy.

## Experimental Section

### Chemistry

Analytical grade solvents purchased from Sigma-Aldrich were used for all reactions. Argon was used to carry out the reactions in an inert atmosphere. Microwave reactions were carried out with an Initiator device (Biotage). Precoated aluminum foils (ALUGRAM Xtra SIL G/UV254, Merk) were used for thin layer chromatography to follow reactions. Melting points were recorded with a Büchi Melting point M-560 apparatus. ^1^H and ^13^C NMR spectra were recorded on a Bruker AV 300 MHz instrument (^1^H NMR, 300 MHz; ^13^C NMR, 75 MHz) or on a 500 MHz instrument (^1^H NMR, 500 MHz; ^13^C NMR, 125 MHz) located at the NMR unit of Research Assistance Centres from Complutense University of Madrid. The abbreviations used are s (singlet), d (doublet), t (triplet), q (quartet), and m (multiplet). Coupling constants (*J*) are expressed in Hz. Mass spectra were acquired on a Thermo Mod. Finnigan LXQ spectrometer coupled to a high-performance liquid instrument equipped with a ZORBAX SB-C18 column (50 mm × 4.6 mm, 3.5 μm packing diameter), using scan positive electrospray ionization (ESI). Column chromatography was performed on silica gel 60 (Merk) manually or automatically using the IsoleraOne instrument (Biotage). High-resolution mass spectra (HRMS-ESI) were recorded on an Agilent 6500 mass spectrometer with an ESI/APCI ionization source and quadrupole/time-of-flight (QTOF) coupled to an Agilent 1200 liquid chromatograph equipped with a Phenomenex Luna C18(2) reversed phase column (100 mm × 2.1 mm, 3 μm packing diameter) located at the Mass Spectrometry Service of the Institute of General Organic Chemistry (IQOG-CSIC). The HPLC conditions for purity assessment were as follows: HPLC Surveyor equipped with a PDA Surveyor plus UV–vis detector; ZORBAX SB-C18 column (3.5 μm, 4.6 mm × 50 mm); H_2_O/CH_3_CN gradient elution from 100/0 to 0/100 for 5, 7, or 10 min; flow rate, 500 or 800 μL/min; wavelength, UV 254 nm. Three different gradient conditions were used.:

- Gradient I: 23 °C, 0.5 mL/min flow rate. Gradient elution with the mobile phases as (A) H_2_O containing 0.1% volume/volume (v/v) formic acid and (B) CH_3_CN containing 0.1% (v/v) formic acid. Gradient conditions were initially 5% B, increasing linearly to 95% B over 5 min, remaining at 95% B for 1.45 min, and then decreasing to 10% B over 0.55 min.
- Gradient II: 23 °C, 0.8 mL/min flow rate. Gradient elution with the mobile phases as (A) H_2_O containing 0.1% volume/volume (v/v) formic acid and (B) CH_3_CN containing 0.1% (v/v) formic acid. Gradient conditions were initially 5% B, increasing linearly to 100% B over 3 min, remaining at 100% B for 1.45 min, and then decreasing to 5% B over 0.55 min.
- Gradient III: 23 °C, 0.8 mL/min flow rate. Gradient elution with the mobile phases as (A) H_2_O containing 0.1% volume/volume (v/v) formic acid and (B) CH_3_CN containing 0.1% (v/v) formic acid. Gradient conditions were initially 10% B, increasing linearly to 95% B over 5 min, remaining at 95% B for 4 min, and then decreasing to 0% B over 1 min.

All the final compounds are >95% pure by HPLC. Confirmation HPLC traces are included in the Supporting Information.

#### General Procedure A for the Synthesis of 5-Phenylthiophene-2-carboxamide Derivatives **2–8** and Bromo-heterocycle Derivatives **9–15** and **65–72**

A solution of the corresponding amine (1.02 equiv.) in CH_2_Cl_2_ was slowly added to a solution of 5-phenylthiophen-2-carboxylic acid or the corresponding bromocarboxylic acid (1 equiv.), 1-ethyl-3-(3-dimethylaminopropyl)carbodiimide (EDCl) (2 equiv.), and Et_3_N (3 equiv.) in CH_2_Cl_2_ at 0 °C. For some compounds, 1-hydroxybenzotriazole (HOBt) (2 equiv.) was added to the mixture. The mixture was stirred 24 h at room temperature for all compounds except for **70** (5 days) and for **71**(4 days). The crude was washed with H_2_O, saturated NH_4_Cl solution, saturated NaCl solution and then dried over Mg_2_SO_4_ anh. The desiccant was filtered off and the volatiles were evaporated to dryness under vacuum. The resulting residues were purified by flash column chromatography using mixtures of solvents as eluents as indicated in each case.

#### General Procedure B for the Synthesis of Hidroxyphenyl-heterocycle Derivatives **16–31** and **83–89**

The corresponding borane derivative (1.2 equiv.) was added to a previously degassed solution of the corresponding carboxamide (1 equiv.), Na_2_CO_3_ (2.2 equiv.), and Pd(PPh_3_)_4_ (0.05 equiv.) in a mixture of toluene/H_2_O/EtOH 2:1.5:1. The mixture was bubbled with argon for 15 min and then was heated under microwave irradiation (MW) for 20 min at 120 °C. The crude was washed with a H_2_O/EtOAc 1:1 solution and was extracted with EtOAc. The organic layer was washed with a 1:1 solution of saturated NaCl solution/H_2_O and then dried over anhydrous Mg_2_SO_4_. The resulting residues were purified by flash column chromatography using mixtures of solvents as eluents as indicated in each case.

#### General Procedure C for the Synthesis of Protected-heterocycle Derivatives **32–46**, **61**, **64**, and **90–96**

To a solution of PPh_3_ (1.3 equiv.) in THF at 0 °C was added diisopropyl azodicarboxylate (DIAD) (1.3 equiv.), except for the synthesis of **90** that used di-(4-chlorobenzyl)azodicarboxylate (DCAD). The mixture was stirred at room temperature until a white turbidity appeared. After cooling again to 0 °C, a solution of the hydroxyphenyl precursors (1 equiv.) and the corresponding protected piperidine indicated in each case (1.3 equiv.) in THF was added. The mixture was stirred at the time indicated in each case. The volatiles were evaporated to dryness under vacuum and the resulting residues were purified by flash column chromatography using mixtures of solvents as eluents indicated in each case.

#### General Procedure D for the Synthesis of Protected-heterocycle Derivatives **62** and **73–77**

For the synthesis of one thiophene and all the *para*-substituted thiazole and furan precursors, an alternative procedure was employed. 4-(4-(4,4,5,5-Tetramethyl-1,3,2-dioxaborolan-2-yl)phenoxy)piperidine-1-carboxylate (**61**) (1 equiv.) was added to a previously degassed solution of the corresponding carboxamide (1 equiv.), Na_2_CO_3_ (2.2 equiv.), and Pd(PPh_3_)_4_ (0.05 equiv.) in a mixture of toluene/H_2_O/EtOH 2:1.5:1. The mixture was bubbled with argon for 15 min and then was heated under microwave irradiation (MW) for 20 min at 120 °C. The crude was washed with a H_2_O/EtOAc 1:1 solution and was extracted with EtOAc. The organic layer was washed with a 1:1 solution of saturated NaCl solution/H_2_O and then dried over anhydrous Mg_2_SO_4_. The resulting residues were purified by flash column chromatography using mixtures of solvents as eluents as indicated in each case.

#### General Procedure E for the Synthesis of (Piperidin-4-yloxy)phenyl-heterocycle Derivatives **1**, **47–60**, **63**, **78–82**, and **97–103**

After obtaining the protected products, deprotection was carried out, stirring products in a solution of CH_2_Cl_2_/TFA 3:2 or CH_2_Cl_2_/TFA 4:1 at room temperature for 3 h except for **81** (4 days), **82** (3 days), **100** (24 h), and **101** (4 days). After completion of the deprotection, the solvent was evaporated under vacuum and the crude solved in CH_2_Cl_2_ was basified with NaHCO_3_ to give the final product in its neutral form. The volatiles were evaporated to dryness under vacuum and the resulting residues were purified by flash column chromatography using mixtures of solvents as eluents as indicated in each case.

##### *N*-Phenyl-5-(2-(piperidin-4-yloxy)phenyl)thiophene-2-carboxamide (**1**)

The title compound was prepared by reaction of *tert*-butyl 4-(2-(5-(phenylcarbamoyl)thiophen-2-yl)phenoxy)piperidine-1-carboxylate (**32**) (0.5 mmol, 302 mg) and a solution of CH_2_Cl_2_ (5 mL) and TFA (3.4 mL) following general procedure E. Purification: CH_2_Cl_2_/ MeOH (9:1). Yield: 90 mg (35%) as a white solid. Mp 122–124 °C. ^1^H NMR (300 MHz, DMSO-*d*_6_) δ 10.28 (s, 1H), 8.07 (d, *J* = 4.1 Hz, 1H), 7.87 (dd, *J* = 7.9, 1.6 Hz, 1H), 7.81–7.73 (m, 2H), 7.70 (d, *J* = 4.1 Hz, 1H), 7.41–7.31 (m, 3H), 7.29 (d, *J* = 7.7 Hz), 7.12 (d, *J* = 7.5 Hz, 1H), 7.07 (dd, *J* = 7.8, 1.2 Hz, 1H), 4.91 (dt, *J* = 7.9, 4.0 Hz), 3.30 (m, 2H), 3.13 (m, 2H), 2.21 (m, 2H), 1.98 (m, 2H). ^13^C NMR (75 MHz, DMSO-*d*_6_) δ 160.5, 152.8, 143.7, 139.5, 139.2, 130.1, 129.0, 128.9, 128.7, 126.1, 124.0, 121.8, 120.6, 114.5, 70.7, 41.3, 27.7. HRMS (ESI) calc. for C_22_H_22_N_2_O_2_S [M + H]^+^ 379.1475; found 379.1473. HPLC-MS (gradient II) (M + H)^+^ = 379, *R*_t_ = 2.86 min (99%).

##### *N*-(4-Chlorophenyl)-5-(2-(piperidin-4-yloxy)phenyl)thiophene-2-carboxamide (**47**)

The title compound was obtained by reaction of *tert*-butyl 4-(2-(5-((4-chlorophenyl)carbamoyl)thiophen-2-yl)phenoxy)piperidine-1-carboxylate (**33**) (0.46 mmol, 102 mg) and a solution of CH_2_Cl_2_ (5 mL) and TFA (3.3 mL) following general procedure E. Compound **47** was obtained pure after acid extraction. Yield: 30 mg (16%) as a white solid. Mp 143–145 °C. ^1^H NMR (300 MHz, DMSO-*d*_6_) δ 10.29(s, 1H) 7.93 (d, *J* = 4.1 Hz, 1H), 7.82 (dd, *J* = 7.9, 1.7 Hz, 1H), 7.78–7.75 (m, 2H), 7.66 (d, *J* = 4.2 Hz, 1H), 7.40–7.35 (m, 2H), 7.31 (ddd, *J* = 8.6, 7.2, 1.7 Hz, 1H), 7.21 (d, *J* = 8.5 Hz, 1H), 7.01 (t, *J* = 7.4 Hz, 1H), 4.69–4.62 (m, 1H), 3.02–2.93 (m, 2H), 2.62–2.53 (m, 2H), 2.01–1.96 (m, 2H), 1.66–1.57 (m, 2H). ^13^C NMR (75 MHz, DMSO-*d*_6_) δ 160.6, 152.9, 143.5, 138.7 (2C), 129.4 (2C), 128.4 (2C), 128.0, 126.9, 125.3, 122.5, 122.0 (2C), 120.8, 114.3, 74.7, 43.6 (2C), 32.2 (2C). HRMS (ESI) calc. for C_22_H_22_ClN_2_O_2_S [M + H]^+^ 413.1085; found 413.1077. HPLC-MS (gradient II) (M + H)^+^ = 413, *R*_t_ = 2.69 min (99%).

##### *N*-(3-Chlorophenyl)-5-(2-(piperidin-4-yloxy)phenyl)thiophene-2-carboxamide (**48**)

The title compound was obtained by reaction of *tert*-butyl 4-(2-(5-((3-chlorophenyl)carbamoyl)thiophen-2-yl)phenoxy)piperidine-1-carboxylate (**34**) (0.33 mmol, 169 mg) and a solution of CH_2_Cl_2_ (3 mL) and TFA (2 mL) following general procedure E. Purification: CH_2_Cl_2_/MeOH (9:1). Yield: 53 mg (39%) as a white solid. Mp 195–197 °C. ^1^H NMR (500 MHz, DMSO-*d*_6_) δ 10.42 (s, 1H), 8.06 (d, *J* = 4.1 Hz, 1H), 7.96 (t, *J* = 2.1 Hz, 1H), 7.86 (dd, *J* = 7.9, 1.7 Hz, 1H), 7.73–7.68 (m, 2H), 7.38 (t, *J* = 8.1 Hz, 1H), 7.33 (ddd, *J* = 8.7, 7.2, 1.7 Hz, 1H), 7.23 (d, *J* = 8.6 Hz, 1H), 7.15 (ddd, *J* = 8.0, 2.1, 0.9 Hz, 1H), 7.05–7.01 (m, 1H), 4.72–4.66 (m, 1H), 3.05–3.00 (m, 2H), 2.67–2.59 (m, 2H), 2.05–1.97 (m, 2H), 1.69–1.61 (m, 2H). ^13^C NMR (125 MHz, DMSO-*d*_6_) δ 160.5, 152.9, 144.2, 140.4, 138.3, 132.9, 130.3, 129.6, 128.9, 128.1, 125.4, 123.2, 122.3, 120.9, 119.6, 118.5, 114.3, 74.4, 43.4 (2C), 31.9 (2C). HRMS (ESI) *m*/*z*: [M + H]^+^ calc. for C_22_H_22_ClN_2_O_2_S 413.1085; found 413.1083. HPLC-MS (gradient II) (M + H)^+^ = 413, *R*_t_ = 2.72 (99%).

##### *N*-Phenethyl-5-(2-(piperidin-4-yloxy)phenyl)thiophene-2-carboxamide (**49**)

The title compound was obtained by reaction of *tert*-butyl 4-(2-(5-(phenethylcarbamoyl)thiophen-2-yl)phenoxy)piperidine-1-carboxylate (**35**) (0.47 mmol, 237 mg) and a solution of CH_2_Cl_2_ (5 mL) and TFA (3.3 mL) following general procedure E. Purification: CH_2_Cl_2_/MeOH (9:1). Yield: 30.5 mg (16%) as a yellow solid. Mp decomposition. ^1^H NMR (300 MHz, DMSO-*d*_6_) δ 8.56 (t, *J* = 5.7 Hz, 1H), 7.78 (dd, *J* = 7.9, 1.7 Hz, 1H), 7.67 (d, *J* = 4.0 Hz, 1H), 7.59 (d, *J* = 4.0 Hz, 1H), 7.33–7.18 (m, 7H), 7.02–6.97 (m, 1H), 4.66–4.56 (m, 1H), 3.46 (q, *J* = 6.9, 6.4 Hz, 2H), 3.02–2.94 (m, 2H), 2.84 (t, *J* = 7.5 Hz, 2H), 2.62–2.53 (m, 2H), 2.00–1.94 (m, 2H), 1.65–1.54 (m, 2H). ^13^C NMR (75 MHz, DMSO-*d*_6_) δ 161.5, 152.9, 142.7, 139.5, 139.0, 129.3, 128.7 (2C), 128.4 (2C), 127.9, 127.1, 126.1, 125.2, 122.6, 120.8, 114.3, 74.6, 43.7 (2C), 40.8, 35.3, 32.3 (2C). HRMS (ESI) *m*/*z*: [M + H]^+^ calc. for C_24_H_27_N_2_O_2_S 407.1788; found 407.1782. HPLC-MS (gradient II) (M + H)^+^ = 407, *R*_t_ = 2.62 (99%).

##### *N*-(4-Morpholinophenyl)-5-(2-(piperidin-4-yloxy)phenyl)thiophene-2-carboxamide (**50**)

The title compound was obtained by reaction of *tert*-butyl 4-(2-(5-((4-morpholinophenyl)carbamoyl)thiophen-2-yl)phenoxy)piperidine-1-carboxylate (**36**) (0.53 mmol, 300 mg) and a solution of CH_2_Cl_2_ (10 mL) and TFA (6.6 mL) following general procedure E. Compound **50** was obtained pure after workup. Yield: 58 mg (24%) as a yellow solid. Mp 184–186 °C. ^1^H NMR (300 MHz, DMSO-*d*_6_) δ 10.05 (s, 1H), 7.96 (d, *J* = 4.1 Hz, 1H), 7.84 (dd, *J* = 7.9, 1.6 Hz, 1H,), 7.67 (d, *J* = 4.1 Hz, 1H), 7.63–7.56 (m, 2H), 7.34 (ddd, *J* = 8.7, 7.1, 1.6 Hz, 1H), 7.28–7.22 (m, 1H), 7.04 (ddd, *J* = 8.0, 7.2, 1.2 Hz, 1H), 6.99–6.90 (m, 2H), 4.84–4.74 (m, 1H), 3.79–3.69 (m, 4H), 3.22–3.11 (m, 2H), 3.11–3.04 (m, 4H), 2.95–2.83 (m, 2H), 2.17–2.03 (m, 2H), 1.90–1.74 (m, 2H). ^13^C NMR (75 MHz, DMSO-*d*_6_) δ 159.7, 152.7, 147.5, 143.1, 139.4, 131.6, 130.9, 129.5, 128.7, 127.9, 125.5, 122.5, 121.4 (2C), 115.3 (2C), 114.2, 72.3, 66.1 (2C), 48.8 (2C), 42.2 (2C), 29.6 (2C). HRMS (ESI) *m*/*z*: [M + H]^+^ calc. for C_26_H_30_N_3_O_3_S 464.2002; found 464.2000. HPLC-MS (gradient II) (M + H)^+^ = 464, *R*_t_ = 2.63 (99%).

##### *N*-(2-(1-Benzylpiperidin-4-yl)ethyl)-5-(2-(piperidin-4-yloxy)phenyl)thiophene-2-carboxamide (**51**)

The title compound was obtained by reaction of *tert*-butyl 4-(2-(5-((2-(1-benzylpiperidin-4-yl)ethyl)carbamoyl)thiophen-2-yl)phenoxy)piperidine-1-carboxylate (**37**) (0.25 mmol, 150 mg) and a solution of CH_2_Cl_2_ (3 mL) and TFA (2 mL) following general procedure E. Purification: EtOAc/MeOH (8:2). Yield: 30 mg (24%) as a yellow solid. Mp 118–120 °C. ^1^H NMR (300 MHz, DMSO-*d*_6_) δ 8.39 (t, *J* = 5.7 Hz, 1H), 7.77 (dd, *J* = 7.9, 1.7 Hz, 1H), 7.68 (d, *J* = 4.0 Hz, 1H), 7.58 (d, *J* = 4.0 Hz, 1H), 7.33–7.26 (m, 5H), 7.26–7.18 (m, 2H), 7.02–6.97 (m, 1H), 4.67–4.58 (m, 1H), 3.42 (s, 2H), 3.28–3.23 (m, 2H), 3.01–2.95 (m, 2H), 2.81–2.74 (m, 2H), 2.58 (ddt, 2H), 2.01–1.94 (m, 2H), 1.92–1.86 (m, 2H), 1.70–1.64 (m, 2H), 1.64–1.55 (m, 2H), 1.46 (q, *J* = 7.0 Hz, 2H), 1.35–1.26 (m, 1H), 1.20–1.12 (m, 2H). ^13^C NMR (75 MHz, DMSO-*d*_6_) δ 161.3, 152.9, 142.6, 139.2, 138.7, 129.2, 128.7 (2C), 128.1 (2C), 128.0, 127.0, 126.7, 125.2, 122.6, 120.8, 114.3, 74.5, 62.5, 53.3 (2C), 43.6 (2C), 36.7, 36.0, 32.9, 32.2 (2C), 31.9 (2C). HRMS (ESI) *m*/*z*: [M + H]^+^ calc. for C_30_H_38_N_3_O_2_S 504.2679; found 504.2680. HPLC-MS (gradient II) (M + H)^+^ = 504, *R*_t_ = 1.84 min (99%).

##### (5-(2-(Piperidin-4-yloxy)phenyl)thiophen-2-yl)(pyrrolidin-1-yl)methanone (**52**)

The title compound was obtained by reaction of *tert*-butyl 4-(2-(5-(pyrrolidine-1-carbonyl)thiophen-2-yl)phenoxy)piperidine-1-carboxylate (**38**) (0.85 mmol, 386 mg) and a solution of CH_2_Cl_2_ (15 mL) and TFA (10 mL) following general procedure E. Purification: CH_2_Cl_2_/MeOH (9:1). Yield: 213 mg (71%) as a white solid. Mp 124.5–126.5 °C. ^1^H NMR (300 MHz, DMSO-*d*_6_) δ 7.80 (dd, *J* = 7.9, 1.7 Hz, 1H), 7.61 (d, *J* = 4.1 Hz, 1H), 7.55 (d, *J* = 4.1 Hz, 1H), 7.30 (ddd, *J* = 8.7, 7.1, 1.7 Hz, 1H), 7.23–7.17 (m, 1H), 7.04–6.96 (m, 1H), 4.70–4.58 (m, 1H), 3.83–3.67 (m, 2H), 3.55–3.42 (m, 2H), 3.04–2.93 (m, 2H), 2.68–2.54 (m, 2H), 2.05–1.78 (m, 6H), 1.67–1.52 (m, 2H). ^13^C NMR (75 MHz, DMSO-*d*_6_) δ 160.8, 152.9, 142.3, 138.8, 129.3, 128.9, 128.0, 125.0, 122.4, 120.8, 114.2, 74.4 48.3, 47.1, 43.5 (2C), 32.1 (2C), 26.3, 23.5. HRMS (ESI) *m*/*z*: [M + H]^+^ calc. for C_20_H_25_N_2_O_2_S 357.1631; found 357.1616. HPLC-MS (gradient II) (M + H)^+^ = 357, *R*_t_ = 2.25 min (99%).

##### *N*-Phenyl-5-(3-(piperidin-4-yloxy)phenyl)thiophene-2-carboxamide (**53**)

The title compound was obtained by reaction of *tert*-butyl 4-(3-(5-(phenylcarbamoyl)thiophen-2-yl)phenoxy)piperidine-1-carboxylate (**39**) (1 mmol, 478 mg) and a solution of CH_2_Cl_2_ (8 mL) and TFA (2 mL) following general procedure E. Purification: CH_2_Cl_2_/MeOH (9:1). Yield: 121 mg (32%) as a white solid. Mp 151–153 °C. ^1^H NMR (300 MHz, DMSO-*d*_6_) δ 10.27 (s, 1H), 8.05 (d, *J* = 4.0 Hz, 1H), 7.81–7.70 (m, 2H), 7.65 (d, *J* = 4.0 Hz, 1H), 7.36 (m, 3H), 7.28 (m, 2H), 7.21–7.05 (m, 1H), 6.99 (d, *J* = 8.1 Hz, 1H), 4.56 (dt, *J* = 8.8, 4.7 Hz, 1H), 3.08–2.90 (m, 2H), 2.67 (m, 2H), 1.96 (dd, *J* = 12.9, 4.0 Hz, 2H), 1.53 (qd, *J* = 9.2, 4.7 Hz, 2H). ^13^C NMR (75 MHz, DMSO-*d*_6_) δ 160.0, 157.9, 148.5, 139.2, 139.0, 134.8, 130.9, 130.5, 129.0, 125.1, 124.1, 120.7, 118.6, 116.4, 113.4, 73.2, 43.5, 31.9. HRMS (ESI) *m*/*z*: [M + H]^+^ calc. for C_22_H_23_N_2_O_2_S 379.1475; found 379.1475. HPLC-MS (gradient II) (M + H)^+^ = 379, *R*_t_ = 2.91 min (99%).

##### *N*-(4-Chlorophenyl)-5-(3-(piperidin-4-yloxy)phenyl)thiophene-2-carboxamide (**54**)

The title compound was obtained by reaction of *tert*-butyl 4-(3-(5-((4-chlorophenyl)carbamoyl)thiophen-2-yl)phenoxy)piperidine-1-carboxylate (**40**) (1 mmol, 508 mg) and a solution of CH_2_Cl_2_ (8 mL) and TFA (2 mL) following general procedure E. Purification: CH_2_Cl_2_/MeOH (9:1). Yield: 137 mg (34%) as a white solid. Mp 169–171 °C. ^1^H NMR (300 MHz, DMSO-*d*_6_) δ 10.36 (s, 1H), 8.02 (d, *J* = 4.0 Hz, 1H), 7.82–7.74 (m, 2H), 7.65 (d, *J* = 3.9 Hz, 1H), 7.47–7.39 (m, 2H), 7.35 (t, *J* = 8.1 Hz, 1H), 7.30–7.23 (m, 2H), 7.02–6.94 (m, 1H), 4.64–4.37 (m, 1H), 3.02–2.88 (m, 2H), 2.67–2.54 (m, 2H), 2.02–1.86 (m, 2H), 1.58–1.38 (m, 2H). ^13^C NMR (75 MHz, DMSO-*d*_6_) δ 159.7, 157.7, 148.5, 138.4, 137.7, 134.3, 130.5, 130.4, 128.6 (2C), 127.4, 124.8, 121.8 (2C), 118.1, 116.1, 113.0, 73.5, 43.7 (2C), 32.3 (2C). HRMS (ESI) *m*/*z*: [M + H]^+^ calc. for C_22_H_22_ClN_2_O_2_S 413.1085; found 413.1088. HPLC-MS (gradient II) (M + H)^+^ = 413, *R*_t_ = 2.90 min (99%).

##### *N*-(3-Chlorophenyl)-5-(3-(piperidin-4-yloxy)phenyl)thiophene-2-carboxamide (**55**)

The title compound was obtained by reaction of *tert*-butyl 4-(3-(5-((3-chlorophenyl)carbamoyl)thiophen-2-yl)phenoxy)piperidine-1-carboxylate (**41**) (0.27 mmol, 139 mg) and a solution of CH_2_Cl_2_ (3 mL) and TFA (2 mL) for 3 h following general procedure E. Purification: CH_2_Cl_2_/MeOH (9:1). Yield: 17 mg (16%) as a yellow solid. Mp 150–152 °C. ^1^H NMR (300 MHz, DMSO-*d*_6_) δ 10.40 (s, 1H), 8.03 (d, *J* = 4.0 Hz, 1H), 7.92 (t, *J* = 2.1 Hz, 1H), 7.72–7.63 (m, 2H), 7.43–7.25 (m, 4H), 7.20–7.14 (m, 1H), 7.03–6.95 (m, 1H), 4.62–4.45 (m, 1H), 3.04–2.91 (m, 2H), 2.69–2.57 (m, 2H), 2.02–1.90 (m, 2H), 1.58–1.41 (m, 2H), 1.27–1.10 (m, 1H). ^13^C NMR (75 MHz, DMSO-*d*_6_) δ 159.9, 157.6, 148.7, 140.2, 138.3, 134.3, 133.0, 130.6, 130.5, 130.4, 124.8, 123.4, 119.7, 118.6, 118.2, 116.1, 113.1, 73.3, 43.5 (2C), 31.9 (2C). HRMS (ESI) *m*/*z*: [M + H]^+^ calc. for C_22_H_22_ClN_2_O_2_S 413.1085; found 413.1080. HPLC-MS (gradient II) (M + H)^+^ = 413, *R*_t_ = 2.75 (99%).

##### *N*-Phenethyl-5-(3-(piperidin-4-yloxy)phenyl)thiophene-2-carboxamide (**56**)

The title compound was obtained by reaction of *tert*-butyl 4-(3-(5-(phenethylcarbamoyl)thiophen-2-yl)phenoxy)piperidine-1-carboxylate (**42**) (0.45 mmol, 230 mg) and a solution of CH_2_Cl_2_ (5 mL) and TFA (2.25 mL) following general procedure E. Compound **56** was obtained pure after workup. Yield: 145 mg (79%) as a white solid. Mp 152–154 °C. ^1^H NMR (300 MHz, DMSO-*d*_6_) δ 8.62 (t, *J* = 5.6 Hz, 1H), 7.71 (d, *J* = 4.0 Hz, 1H), 7.54 (d, *J* = 3.9 Hz, 1H), 7.37–7.16 (m, 8H), 6.96 (ddd, *J* = 8.2, 2.4, 1.1 Hz, 1H), 4.52 (tt, *J* = 8.7, 3.9 Hz, 1H), 3.46 (dt, *J* = 8.1, 6.2 Hz, 2H), 3.05–2.92 (m, 2H), 2.84 (t, *J* = 8.3 Hz, 2H), 2.67–2.59 (m, 2H), 2.02–1.89 (m, 2H), 1.58–1.39 (m, 2H). ^13^C NMR (75 MHz, DMSO-*d*_6_) δ 160.8, 157.6, 147.0, 139.4, 139.0, 134.5, 130.4, 128.8, 128.7 (2C), 128.4 (2C), 126.1, 124.5, 118.1, 115.8, 112.9, 73.1, 43.3 (2C), 40.8, 35.2, 31.8 (2C). HRMS (ESI) *m*/*z*: [M + H]^+^ calc. for C_24_H_27_N_2_O_2_S 407.1788; found 407.1781. HPLC-MS (gradient II) (M + H)^+^ = 407, *R*_t_ = 2.63 (97%).

##### *N*-Phenyl-5-(4-(piperidin-4-yloxy)phenyl)thiophene-2-carboxamide (**57**)

The title compound was obtained by reaction of *tert*-butyl 4-(4-(5-(phenylcarbamoyl)thiophen-2-yl)phenoxy)piperidine-1-carboxylate (**43**) (0.20 mmol, 96 mg) and a solution of CH_2_Cl_2_ (3.2 mL) and TFA (0.8 mL) following general procedure E. Purification: CH_2_Cl_2_/MeOH (9:1). Yield: 29 mg (38%) as a white solid. Mp 192–194 °C. ^1^H NMR (300 MHz, DMSO-*d*_6_) δ 10.22 (s, 1H), 8.01 (d, *J* = 4.0 Hz, 1H), 7.79–7.68 (m, 2H), 7.68–7.57 (m, 2H), 7.49 (d, *J* = 3.9 Hz, 1H), 7.36 (t, *J* = 7.9 Hz, 2H), 7.15–7.07 (m, 1H), 7.07–6.94 (m, 2H), 4.69–4.38 (m, 1H), 2.98 (dd, *J* = 12.6, 4.8 Hz, 2H), 2.65 (ddd, *J* = 12.8, 9.9, 2.9 Hz, 2H), 2.04–1.86 (m, 2H), 1.51 (m, 2H). ^13^C NMR (75 MHz, DMSO-*d*_6_) δ 160.1, 157.9, 148.9, 139.1, 137.9, 130.7, 129.0, 127.6, 125.9, 124.0, 123.5, 120.7, 116.7, 73.3, 43.6, 31.9. HRMS (ESI) *m*/*z*: [M + H]^+^ calc. for C_22_H_23_N_2_O_2_S 379.1475; found 379.1469. HPLC-MS (gradient II) (M + H)^+^ = 379, *R*_t_ = 2.82 min (99%).

##### *N*-(4-Chlorophenyl)-5-(4-(piperidin-4-yloxy)phenyl)thiophene-2-carboxamide (**58**)

The title compound was obtained by reaction of *tert*-butyl 4-(4-(5-((4-chlorophenyl)carbamoyl)thiophen-2-yl)phenoxy)piperidine-1-carboxylate (**44**) (0.20 mmol, 102 mg) and a solution of CH_2_Cl_2_ (3.2 mL) and TFA (0.8 mL) following general procedure E. Purification: CH_2_Cl_2_/MeOH (9:1). Yield: 45 mg (55%) as a white solid. Mp 240–242 °C. ^1^H NMR (300 MHz, DMSO-*d*_6_) δ 10.40 (s, 1H), 8.04 (d, *J* = 4.0 Hz, 1H), 7.84–7.76 (m, 2H), 7.70–7.64 (m, 2H), 7.50 (d, *J* = 4.0 Hz, 1H), 7.44–7.37 (m, 2H), 7.11–7.04 (m, 2H), 4.70–4.59 (m, 1H), 3.19–3.08 (m, 2H), 2.99–2.85 (m, 2H), 2.07–1.98 (m, 2H), 1.81–1.64 (m, 2H). ^13^C NMR (75 MHz, DMSO-*d*_6_) δ 159.8, 157.2, 148.7, 137.8, 137.4, 130.6, 128.6 (2C), 127.3 (3C), 125.9, 123.3, 121.8 (2C), 116.4 (2C), 70.6, 41.5 (2C), 28.8 (2C). HRMS (ESI) *m*/*z*: [M + H]^+^ calc. for C_22_H_22_ClN_2_O_2_S 413.1085; found 413.1080. HPLC-MS (gradient II) (M + H)^+^ = 413, *R*_t_ = 2.77 (95%).

##### *N*-(3-Chlorophenyl)-5-(4-(piperidin-4-yloxy)phenyl)thiophene-2-carboxamide (**59**)

The title compound was obtained by reaction of *tert*-butyl 4-(4-(5-((3-chlorophenyl)carbamoyl)thiophen-2-yl)phenoxy)piperidine-1-carboxylate (**45**) (0.42 mmol, 213 mg) and a solution of CH_2_Cl_2_ (4 mL) and TFA (1 mL) following general procedure E. Purification: CH_2_Cl_2_/MeOH (9:1). Yield: 38 mg (22%) as a white solid. Mp 224–226 °C. ^1^H NMR (300 MHz, DMSO-*d*_6_) δ 10.37 (s, 1H), 8.02 (d, *J* = 4.0 Hz, 1H), 7.92 (t, *J* = 2.0 Hz, 1H), 7.73–7.61 (m, 3H), 7.50 (d, *J* = 3.9 Hz, 1H), 7.39 (t, *J* = 8.1 Hz, 1H), 7.20–7.13 (m, 1H), 7.09–6.99 (m, 2H), 4.58–4.40 (m, 1H), 3.05–2.91 (m, 2H), 2.71–2.58 (m, 2H), 2.09–1.87 (m, 2H), 1.61–1.40 (m, 2H). ^13^C NMR (75 MHz, DMSO) δ 159.9, 157.6, 149.1, 140.3, 137.0, 132.9, 130.8, 130.4, 127.3 (2C), 125.5, 123.3, 123.2, 119.6, 118.5, 116.4 (2C), 72.9, 43.2 (2C), 31.5 (2C). HRMS (ESI) *m*/*z*: [M + H]^+^ calc. for C_22_H_22_ClN_2_O_2_S 413.1085; found 413.1083. HPLC-MS (gradient I) (M + H)^+^ = 413, *R*_t_ = 4.46 (99%).

##### *N*-Phenethyl-5-(4-(piperidin-4-yloxy)phenyl)thiophene-2-carboxamide (**60**)

The title compound was obtained by reaction of *tert*-butyl 4-(4-(5-(phenethylcarbamoyl)thiophen-2-yl)phenoxy)piperidine-1-carboxylate (**46**) (1.15 mmol, 585 mg) and a solution of CH_2_Cl_2_ (8 mL) and TFA (2 mL) following general procedure E. Compound **60** was obtained pure after workup. Yield: 298 mg (64%) as a white solid. Mp 174–176 °C. ^1^H NMR (300 MHz, DMSO-*d*_6_) δ 8.56 (t, *J* = 5.6 Hz, 1H), 7.67 (d, *J* = 3.9 Hz, 1H), 7.64–7.55 (m, 2H), 7.37 (d, *J* = 3.9 Hz, 1H), 7.35–7.17 (m, 5H), 7.00 (d, *J* = 8.8 Hz, 2H), 4.44 (dt, *J* = 9.7, 5.4 Hz, 1H), 3.53–3.40 (m, 2H), 3.01–2.89 (m, 2H), 2.89–2.78 (m, 2H), 2.67–2.53 (m, 2H), 1.99–1.86 (m, 2H), 1.54–1.36 (m, 2H). ^13^C NMR (75 MHz, DMSO-*d*_6_) δ 160.9, 157.4, 147.4, 139.4, 137.8, 128.9, 128.6 (2C), 128.3 (2C), 127.0 (2C), 126.1, 125.6, 122.9, 116.3 (2C), 73.5, 43.6 (2C), 40.7, 35.2, 32.2 (2C). HRMS (ESI) *m*/*z*: [M + H]^+^ calc. for C_24_H_27_N_2_O_2_S 407.1788; found 407.1797. HPLC-MS (gradient II) (M + H)^+^ = 407, *R*_t_ = 2.61 (99%).

##### *N*-(2-(1-Benzylpiperidin-4-yl)ethyl)-5-(4-(piperidin-4-yloxy)phenyl)thiophene-2-carboxamide (**63**)

The title compound was obtained by reaction of *tert*-butyl 4-(4-(5-((2-benzylpiperidin-4-yl)ethyl)carbamoyl)thiophen-2-yl)phenoxy)piperidine-1 carboxylate (**62**) (0.69 mmol, 420 mg) and a solution of CH_2_Cl_2_ (8 mL) and TFA (2 mL) for 3 h following general procedure E. Purification: CH_2_Cl_2_/MeOH (9:1). Yield: 10 mg (3%) as an orange solid. Mp decomposition. ^1^H NMR (300 MHz, DMSO-*d*_6_) δ 8.42 (t, *J* = 5.6 Hz, 1H), 7.69 (d, *J* = 3.9 Hz, 1H), 7.62 (d, *J* = 8.8 Hz, 2H), 7.39 (d, *J* = 3.9 Hz, 1H), 7.33–7.24 (m, 5H), 7.05 (d, *J* = 8.8 Hz, 2H), 4.69–4.61 (m, 1H), 3.43 (s, 2H), 3.25 (m, 2H), 3.21–3.15 (m, 2H), 3.03–2.94 (m, 2H), 2.78 (d, *J* = 11.5 Hz, 2H), 2.11–2.04 (m, 2H), 1.93–1.86 (m, 2H), 1.82–1.73 (m, 2H), 1.66 (d, *J* = 11.4 Hz, 2H), 1.45 (q, *J* = 7.0 Hz, 2H), 1.20–1.13 (m, 3H). ^13^C NMR (75 MHz, DMSO-*d*_6_) δ 160.8, 156.9, 147.1, 138.2, 128.8, 128.7, 128.1 (4C), 127.1, 126.8 (2C), 126.2, 123.1, 116.4 (2C), 69.9, 62.5, 53.2 (2C), 41.1 (2C), 36.7, 35.9, 31.8 (2C), 28.9, 28.1 (2C). HRMS (ESI) *m*/*z*: [M + H]^+^ calc. for C_30_H_38_N_3_O_2_S 504.2679; found 504.2653. HPLC-MS (gradient II) (M + H)^+^ = 504, *R*_t_ = 2.95 min (97%).

##### *N*-Phenyl-2-(4-(piperidin-4-yloxy)phenyl)thiazole-5-carboxamide (**78**)

The title compound was obtained by reaction of *tert*-butyl 4-(4-(5-(phenylcarbamoyl)thiazol-2-yl)phenoxy)piperidine-1-carboxylate (**73**) (0.10 mmol, 50 mg) and a solution of CH_2_Cl_2_ (4 mL) and TFA (1 mL) following general procedure E. Compound **78** was obtained pure after workup. Yield: 38 mg (97%) as a yellow solid. Mp 159–161 °C. ^1^H NMR (300 MHz, DMSO-*d*_6_) δ 10.50 (s, 1H), 8.66 (s, 1H), 7.98–7.89 (m, 2H), 7.77–7.69 (m, 2H), 7.41–7.33 (m, 2H), 7.17–7.05 (m, 3H), 4.62–4.45 (m, 1H), 3.07–2.88 (m, 2H), 2.65–2.52 (m, 2H), 2.01–1.88 (m, 2H), 1.56–1.41 (m, 2H). ^13^C NMR (75 MHz, DMSO-*d*_6_) δ 170.8, 159.6, 158.6, 144.8, 138.5, 134.5, 128.8 (2C), 128.3 (2C), 125.1, 124.0, 120.4 (2C), 116.2 (2C), 73.8, 43.6 (2C), 32.2 (2C). HRMS (ESI) *m*/*z*: [M + H]^+^ calc. for C_21_H_22_N_3_O_2_S 380.1427; found 380.1429. HPLC-MS (gradient II) (M + H)^+^ = 380, *R*_t_ = 2.49 (96%).

##### *N*-(2-(1-Benzylpiperidin-4-yl)ethyl)-2-(4-(piperidin-4-yloxy)phenyl)thiazole-5-carboxamide (**79**)

The title compound was obtained by reaction of *tert*-butyl 4-(4-(5-((2-(1-benzylpiperidin-4-yl)ethyl)carbamoyl)thiazol-2-yl)phenoxy)piperidine-1-carboxylate (**74**) (0.32 mmol, 195 mg) and a solution of CH_2_Cl_2_ (4 mL) and TFA (1 mL) following general procedure E. Compound **79** was obtained pure after workup. Yield: 91 mg (56%) as a yellow solid. Mp 162–164 °C. ^1^H NMR (300 MHz, DMSO-*d*_6_) δ 8.61 (s, 1H), 8.34 (s, 1H), 7.93–7.83 (m, 2H), 7.35–7.18 (m, 5H), 7.11–7.02 (m, 2H), 4.60–4.44 (m, 1H), 3.42 (s, 2H), 3.27–3.22 (m, 2H), 3.04–2.90 (m, 2H), 2.84–2.70 (m, 2H), 2.68–2.54 (m, 2H), 1.99–1.83 (m, 4H), 1.72–1.61 (m, 2H), 1.53–1.41 (m, 4H), 1.36–1.25 (m, 1H), 1.22–1.09 (m, 2H). ^13^C NMR (75 MHz, DMSO-*d*_6_) δ 169.8, 159.6, 159.4, 143.5, 138.7, 134.5, 128.7 (2C), 128.1 (2C), 128.1 (2C), 126.7, 125.2, 116.2 (2C), 73.4, 62.5, 53.2 (2C), 43.4 (2C), 36.9, 35.9, 32.9 (2C), 31.8 (2C). HRMS (ESI) *m*/*z*: [M + H]^+^ calc. for C_29_H_37_N_4_O_2_S 505.2632; found 505.2654. HPLC-MS (gradient II) (M + H)^+^ = 505, *R*_t_ = 2.01 (98%).

##### *N*-Phenyl-5-(4-(piperidin-4-yloxy)phenyl)furan-2-carboxamide (**80**)

The title compound was obtained by reaction of *tert*-butyl 4-(4-(5-(phenylcarbamoyl)furan-2-yl)phenoxy)piperidine-1-carboxylate (**75**) (1.25 mmol, 580 mg) and a solution of CH_2_Cl_2_ (4 mL) and TFA (1 mL) following general procedure E. Compound **80** was obtained pure after workup. Yield: 246 mg (50%) as a yellow solid. Mp 169–171 °C. ^1^H NMR (300 MHz, DMSO-*d*_6_) δ 10.12 (s, 1H), 7.96–7.84 (m, 2H), 7.81–7.71 (m, 2H), 7.42–7.32 (m, 3H), 7.15–7.04 (m, 3H), 7.01 (d, *J* = 3.6 Hz, 1H), 4.63–4.49 (m, 1H), 3.12–2.96 (m, 2H), 2.77–2.62 (m, 2H), 2.07–1.90 (m, 2H), 1.68–1.47 (m, 2H). ^13^C NMR (75 MHz, DMSO-*d*_6_) δ 157.5, 156.1, 155.5, 145.9, 138.5, 128.6 (2C), 126.2, 123.8, 122.1, 122.0, 120.6 (2C), 117.1, 116.0 (2C), 106.2, 72.5, 42.9 (2C), 31.1 (2C). HRMS (ESI) *m*/*z*: [M + H]^+^ calc. for C_22_H_23_N_2_O_3_ 363.1703; found 363.1703. HPLC-MS (gradient II) (M + H)^+^ = 363, *R*_t_ = 2.61 (96%).

##### *N*-(3-Chlorophenyl)-5-(4-(piperidin-4-yloxy)phenyl)furan-2-carboxamide (**81**)

The title compound was obtained by reaction of *tert*-butyl 4-(4-(5-((3-chlorophenyl)carbamoyl)furan-2-yl)phenoxy)piperidine-1-carboxylate (**76**) (0.47 mmol, 240 mg) and a solution of CH_2_Cl_2_ (4 mL) and TFA (1 mL) following general procedure E for 4 days. Purification: CH_2_Cl_2_/MeOH (9:1). Yield: 20 mg (12%) as a yellow solid. Mp 127.5–129.5 °C. ^1^H NMR (300 MHz, DMSO-*d*_6_) δ 10.26 (s, 1H), 7.95–7.93 (m, 1H), 7.90–7.86 (m, 2H), 7.74–7.71 (m, 1H), 7.43–7.38 (m, 2H), 7.19–7.15 (m, 1H), 7.11–7.06 (m, 2H), 7.03 (d, *J* = 3.7 Hz, 1H), 4.61–4.53 (m, 1H), 3.09–2.99 (m, 2H), 2.80–2.68 (m, 2H), 2.04–1.94 (m, 2H), 1.63–1.53 (m, 2H). ^13^C NMR (75 MHz, DMSO-*d*_6_) δ 157.5, 156.2, 155.8, 145.5, 140.1, 132.9, 130.4, 126.3 (2C), 123.4, 122.0, 119.9, 118.8, 117.7, 116.1 (2C), 106.3, 72.1, 42.7 (2C), 30.7 (2C). HRMS (ESI): calc. for C_22_H_22_ClN_2_O_3_ [M + H]^+^ 397.1313; found 397.1312. HPLC-MS (gradient II) (M + H)^+^ = 397, *R*_t_ = 4.09 (99%).

##### *N*-(2-(1-Benzylpiperidin-4-yl)ethyl)-5-(4-(piperidin-4-yloxy)phenyl)furan-2-carboxamide (**82**)

The title compound was obtained by reaction of *tert*-butyl 4-(4-(5-((2-(1-benzylpiperidin-4-yl)ethyl)carbamoyl)furan-2-yl)phenoxy)piperidine-1-carboxylate (**77**) (0.085 mmol, 50 mg) and a solution of CH_2_Cl_2_ (2.25 mL) and TFA (0.75 mL) following general procedure E for 3 days. Purification: CH_2_Cl_2_/MeOH (9:1). Yield: 10 mg (24%) as an orange solid. Mp decomposition. ^1^H NMR (300 MHz, DMSO-*d*_6_) δ 8.40–8.37 (m, 1H), 7.83–7.81 (m, 2H), 7.34–7.26 (m, 4H), 7.25–7.21 (m, 1H), 7.10 (d, *J* = 3.5 Hz, 1H), 7.09–7.06 (m, 2H), 6.92 (d, *J* = 3.5 Hz, 1H), 4.69–4.60 (m, 1H), 3.43 (s, 2H), 3.26–3.21 (m, 2H), 3.20–3.14 (m, 2H), 3.00–2.91 (m, 2H), 2.80–2.74 (m, 2H), 2.08–2.01 (m, 2H), 1.93–1.86 (m, 2H), 1.75–1.65 (m, 4H), 1.48–1.44 (m, 2H), 1.20–1.14 (m, 3H). ^13^C NMR (75 MHz, DMSO-*d*_*6*_) δ 157.6, 156.9, 154.4, 146.6, 138.6, 128.7 (2C), 128.1 (2C), 126.8, 126.0 (2C), 122.6, 116.1 (2C), 115.3, 105.9, 70.3, 62.5, 53.2 (2C), 41.4 (2C), 36.1, 32.9, 31.8 (2C), 29.1 (2C) 22.1. HRMS (ESI): calc. for C_30_H_38_N_3_O_3_ [M + H]^+^ 488.2908; found 488.2921. HPLC-MS (gradient II) (M + H)^+^ = 488, *R*_t_ = 2.88 (97%).

##### *N*-Phenethyl-2-(2-(piperidin-4-yloxy)phenyl)thiazole-5-carboxamide (**97**)

The title compound was obtained by reaction of *tert*-butyl 4-(2-(5-(phenethylcarbamoyl)thiazol-2-yl)phenoxy)piperidine-1-carboxylate (**90**) (0.052 mmol, 26 mg) and a solution of CH_2_Cl_2_ (4 mL) and TFA (1 mL) following general procedure E. Compound **97** was obtained pure after workup. Yield: 16.5 mg (77%) as a white solid. Mp 157–159 °C. ^1^H NMR (300 MHz, DMSO-*d*_6_) δ 8.77 (t, *J* = 5.7 Hz, 1H), 8.42 (s, 1H), 8.32 (dd, *J* = 7.9, 1.8 Hz, 1H), 7.46 (ddd, *J* = 8.7, 7.2, 1.8 Hz, 1H), 7.35–7.28 (m, 3H), 7.29–7.24 (m, 2H), 7.24–7.19 (m, 1H), 7.11–7.05 (m, 1H), 4.78 (dt, *J* = 9.3, 4.9 Hz, 1H), 3.53–3.44 (m, 2H), 3.08–3.00 (m, 2H), 2.85 (t, *J* = 7.5 Hz, 2H), 2.70–2.60 (m, 2H), 2.07–2.01 (m, 2H), 1.74–1.61 (m, 2H). ^13^C NMR (75 MHz, DMSO-*d*_6_) δ 164.0, 160.4, 154.1, 142.0, 139.3, 134.9, 131.7, 128.7 (2C), 128.4 (2C), 127.9, 126.2, 121.8, 120.7, 114.0, 75.2, 43.7 (2C), 40.8, 35.1, 32.2 (2C). HRMS (ESI) *m*/*z*: [M + H]^+^ calc. for C_23_H_26_N_3_O_2_S 408.1740; found 408.1741. HPLC-MS (gradient II) (M + H)^+^ = 408, *R*_t_ = 2.54 (99%).

##### *N*-Phenyl-2-(3-(piperidin-4-yloxy)phenyl)thiazole-5-carboxamide (**98**)

The title compound was obtained by reaction of *tert*-butyl 4-(3-(5-(phenylcarbamoyl)thiazol-2-yl)phenoxy)piperidine-1-carboxylate (**91**) (0.14 mmol, 70 mg) and a solution of CH_2_Cl_2_ (3 mL) and TFA (0.75 mL) following general procedure E. Purification: CH_2_Cl_2_/MeOH (9:1). Yield: 6 mg (11%) as a yellow solid. Mp 235–237 °C. ^1^H NMR (500 MHz, DMSO-*d*_6_) δ 10.55 (s, 1H), 8.74 (s, 1H), 7.74 (d, *J* = 7.8 Hz, 2H), 7.63–7.58 (m, 2H), 7.47 (t, *J* = 8.0 Hz, 1H), 7.38 (t, *J* = 7.9 Hz, 2H), 7.22–7.18 (m, 1H), 7.16–7.12 (m, 1H), 4.82–4.72 (m, 1H), 3.24–3.17 (m, 2H), 3.08–2.99 (m, 2H), 2.15–2.06 (m, 2H), 1.86–1.77 (m, 2H). ^13^C NMR (125 MHz, DMSO-*d*_6_) δ 170.3, 158.4, 157.2, 144.9, 138.4, 135.9, 134.0, 130.9, 129.5, 128.9 (2C), 124.2, 120.4 (2C), 118.5, 113.8, 70.0, 40.2 (2C), 27.9 (2C). HRMS (ESI) *m*/*z*: [M + H]^+^ calc. for C_21_H_22_N_3_O_2_S 380.1427; found 380.1423. HPLC-MS (gradient II) (M + H)^+^ = 380, *R*_t_ = 2.64 (99%).

##### *N*-(4-Chlorophenyl)-5-(2-(piperidin-4-yloxy)phenyl)furan-2-carboxamide (**99**)

The title compound was obtained by reaction of *tert*-butyl 4-(2-(5-((4-chlorophenyl)carbamoyl)furan-2-yl)phenoxy)piperidine-1-carboxylate (**93**) (1.1 mmol, 537 mg) and a solution of CH_2_Cl_2_ (20 mL) and TFA (5 mL) following general procedure E. Purification: CH_2_Cl_2_/MeOH (9:1). Yield: 145 mg (34%) as a white solid. Mp 124.5–126.5 °C. ^1^H NMR (300 MHz, DMSO-*d*_6_) δ 10.31 (s, 1H), 8.16 (dd, *J* = 7.8, 1.7 Hz, 1H), 7.86–7.76 (m, 2H), 7.47–7.40 (m, 3H), 7.39–7.32 (m, 1H), 7.23 (d, *J* = 8.3 Hz, 1H), 7.13–7.04 (m, 2H), 4.76–4.62 (m, 1H), 3.11–2.93 (m, 2H), 2.83–2.64 (m, 2H), 2.14–1.97 (m, 2H), 1.75–1.57 (m, 2H). ^13^C NMR (75 MHz, DMSO-*d*_6_) δ 156.2, 153.6, 152.3, 145.3, 137.5, 129.8, 128.6 (2C), 127.5, 127.0, 122.2 (2C), 120.6, 118.5, 117.3, 113.9, 111.8, 73.1, 43.0 (2C), 31.1 (2C). HRMS (ESI): calc. for C_22_H_22_ClN_2_O_3_ [M + H]^+^ 397.1313; found 397.1312. HPLC-MS (gradient II) (M + H)^+^ = 397, *R*_t_ = 2.78 (99%).

##### *N*-Phenyl-5-(2-(piperidin-4-yloxy)phenyl)furan-2-carboxamide (**100**)

The title compound was obtained by reaction of *tert*-butyl 4-(2-(5-(phenylcarbamoyl)furan-2-yl)phenoxy)piperidine-1-carboxylate (**92**) (1.3 mmol, 600 mg) and a solution of CH_2_Cl_2_ (8 mL) and TFA (2 mL) following general procedure E for 24 h. Purification: CH_2_Cl_2_/MeOH (9:1). Yield: 70 mg (16%) as an orange solid. Mp 147.5–149.5 °C. ^1^H NMR (300 MHz, DMSO-*d*_6_) δ 10.23–10.16 (m, 1H), 8.20–8.14 (m, 1H), 7.81–7.74 (m, 2H), 7.45 (d, *J* = 3.6 Hz, 1H), 7.42–7.31 (m, 3H), 7.29–7.23 (m, 1H), 7.17–7.08 (m, 2H), 7.07 (d, *J* = 3.6 Hz, 1H), 4.90–4.75 (m, 1H), 3.22–3.16 (m, 2H), 3.05–2.92 (m, 2H), 2.25–2.12 (m, 2H), 1.94–1.78 (m, 2H). ^13^C NMR (75 MHz, DMSO-*d*_6_) δ 156.6, 153.7, 152.4, 146.2, 138.9, 130.3, 129.1 (2C), 127.7, 124.3, 121.4, 121.2 (2C), 119.1, 117.4, 114.4, 112.2, 71.4, 41.9 (2C), 28.9 (2C). HRMS (ESI): calc. for C_22_H_23_N_2_O_3_ [M + H]^+^ 363.1703; found 363.1703. HPLC-MS (gradient II) (M + H)^+^ = 363, *R*_t_ = 2.61 (99%).

##### *N*-(2-(1-Benzylpiperidin-4-yl)ethyl)-5-(2-(piperidin-4-yloxy)phenyl)furan-2-carboxamide (**101**)

The title compound was obtained by reaction of *tert*-butyl 4-(2-(5-((2-(1-benzylpiperidin-4-yl)ethyl)carbamoyl)furan-2-yl)phenoxy)piperidine-1-carboxylate (**94**) (0.33 mmol, 195 mg) and a solution of CH_2_Cl_2_ (4 mL) and TFA (1 mL) following general procedure E for 4 days. Purification: CH_2_Cl_2_/MeOH (9:1). Yield: 30 mg (18%) as a yellow solid. Mp 123.6–125.6 °C. ^1^H NMR (300 MHz, DMSO-*d*_6_) δ 8.48–8.41 (m, 1H), 8.11–8.06 (m, 1H), 7.36–7.26 (m, 5H), 7.25–7.18 (m, 2H), 7.14 (d, *J* = 3.4 Hz, 1H), 7.08–7.03 (m, 1H), 7.00 (d, *J* = 3.6 Hz, 1H), 4.73–4.66 (m, 1H), 3.42 (s, 2H), 3.30–3.24 (m, 2H), 3.10–3.01 (m, 2H), 2.83–2.73 (m, 4H), 2.11–2.02 (m, 2H), 1.92–1.85 (m, 2H), 1.74–1.60 (m, 4H), 1.52–1.43 (m, 2H), 1.25–1.08 (m, 3H). ^13^C NMR (75 MHz, DMSO-*d*_6_) δ 157.5, 153.3, 151.1, 146.2, 138.7, 129.5 (2C), 128.7, 128.1 (2C), 126.8, 120.6, 118.8, 115.2, 113.8 (2C), 111.42, 72.5, 62.5, 53.2 (2C), 42.7 (2C), 36.2 (2C), 32.9, 31.9 (2C), 30.5 (2C). HRMS (ESI): calc. for C_30_H_38_N_3_O_3_NH_4_^+^ [M + H]^+^ 505.3173; found 505.3215. HPLC-MS (gradient II) (M + H)^+^ = 488, *R*_t_ = 2.04 (98%).

##### *N*-Phenethyl-5-(2-(piperidin-4-yloxy)phenyl)furan-2-carboxamide (**102**)

The title compound was obtained by reaction of *tert*-butyl 4-(2-(5-(phenethylcarbamoyl)furan-2-yl)phenoxy)piperidine-1-carboxylate (**95**) (1.51 mmol, 743 mg) and a solution of CH_2_Cl_2_ (8 mL) and TFA (2 mL) following general procedure E. Compound **102** was obtained pure after workup. Yield: 379 mg (64%) as a yellow solid. Mp 201.0–203.0 °C. ^1^H NMR (300 MHz, DMSO-*d*_6_) δ 8.62 (t, *J* = 5.8 Hz, 1H), 8.09 (dd, *J* = 7.9, 1.7 Hz, 1H), 7.39–7.19 (m, 7H), 7.17 (d, *J* = 3.5 Hz, 1H), 7.08 (ddd, *J* = 8.1, 7.2, 1.1 Hz, 1H), 6.99 (d, *J* = 3.6 Hz, 1H), 4.89–4.70 (m, 1H), 3.57–3.42 (m, 2H), 3.19–3.09 (m, 2H), 3.00–2.81 (m, 4H), 2.22–2.05 (m, 2H), 1.91–1.74 (m, 2H). ^13^C NMR (75 MHz, DMSO-*d*_6_) δ 157.6, 153.1, 151.0, 146.2, 139.4, 129.5, 128.6 (2C), 128.4 (2C), 126.8, 126.1, 120.8, 118.8, 115.3, 113.8, 111.4, 71.2, 41.7 (2C), 35.3, 28.9 (2C). HRMS (ESI): calc. for C_24_H_27_N_2_O_3_ [M + H]^+^ 391.2016; found 391.2012. HPLC-MS (gradient II) (M + H)^+^ = 391, *R*_t_ = 2.64 (99%).

##### *N*-Phenyl-5-(3-(piperidin-4-yloxy)phenyl)furan-2-carboxamide (**103**)

The title compound was obtained by reaction of *tert*-butyl 4-(3-(5-(phenylcarbamoyl)furan-2-yl)phenoxy)piperidine-1-carboxylate (**96**) (0.39 mmol, 180 mg) and a solution of CH_2_Cl_2_ (4 mL) and TFA (1 mL) following general procedure E. Compound **103** was obtained pure after workup. Yield: 107 mg (76%) as a white solid. Mp 169–171 °C. ^1^H NMR (300 MHz, DMSO-*d*_6_) δ 10.20 (s, 1H), 7.82–7.70 (m, 2H), 7.58–7.49 (m, 2H), 7.44–7.33 (m, 4H), 7.20 (d, *J* = 3.7 Hz, 1H), 7.16–7.09 (m, 1H), 7.04–6.97 (m, 1H), 4.62–4.45 (m, 1H), 3.08–2.91 (m, 2H), 2.75–2.58 (m, 2H), 2.05–1.88 (m, 2H), 1.60–1.43 (m, 2H). ^13^C NMR (75 MHz, DMSO-*d*_6_) δ 157.5, 156.0, 155.0, 146.7, 138.4, 130.7, 130.1, 128.6 (2C), 123.9, 120.7 (2C), 117.0, 116.9, 115.6, 112.3, 108.3, 72.9, 43.3 (2C), 31.6 (2C). HRMS (ESI): calc. for C_22_H_23_N_2_O_3_ [M + H]^+^ 363.1703; found 363.1705. HPLC-MS (gradient II) (M + H)^+^ = 363, *R*_t_ = 2.64 (99%).

### Antiviral Activity in EBOV-GP-Pseudotyped Viruses

#### Cell Lines

Human embryonic kidney cells (293T/17; ATCC-CRL-11268), baby hamster kidney cells (BHK-21/WI-2, Kerafast #EH1011), and African Green Monkey Cell Line (VeroE6) were cultured in Dulbecco’s modified Eagle medium (DMEM) supplemented with 10% heat-inactivated fetal bovine serum (FBS), 25 μg/mL gentamycin, and 2 mM l-glutamine.

#### Construction of Ebola-GP-Y517S or F630H/W Mutants

For generation of plasmid with single-point mutation Y517S or F630H/W in Ebola virus glycoprotein, plasmid encoding the Ebola-Makona virus glycoprotein mutation Y517S or F630H/W was carried out by following the Q5 Site-Directed Mutagenesis standard protocol (New England BioLabs).

Primer pairs containing the mutation of interest were designed using New England BioLabs web-based design program (listed as follows): EBO GP Y517S_F, CAATTTACATTCCTGGACTACTCAGG; EBO GP Y517S_R, GGGTTGCATTTGGGTTGA; Mak F630H_Fw, TATTCATGATcatGTTGATAAAACCCTTC; Mak F630H_Rev, ATCTGATCAATTTTGTCTGTTATG; Mak F630W_Fw, TATTCATGATtggGTTGATAAAACCCTTC; Mak F630W_R, ATCTGATCAATTTTGTCTGTTATG.

Mutant construction was confirmed by sequencing using an ABI PRISM 3100 Genetic Analyzer (Applied Biosystems) and posterior sequence analysis by Geneious R6 bioinformatics software. All the plasmids were prepared with HiPure Plasmid Filter Maxiprep (Invitrogen) and quantified by spectrophotometry (NanoDrop).

#### Production of Recombinant Viruses with Mayinga, Sudan, or VSV-G GP and VSV Backbone

VSV-G-pseudotyped replication-deficient rVSV-luc recombinant viruses were produced to test the inhibitory activity of selected compounds.

The viral construction was pseudotyped with Zaire Ebola virus envelope glycoprotein (GP) strain Mayinga (GenBank: U23187.1), Sudan (GenBank: NC_006432), or vesicular stomatitis virus envelope GP (VSV-G) and expressed luciferase as a reporter of the infection.

For other experiments, Ebola Makona (GeneBank KM 233069.1) or Makona mutants Y517S or F630H/W were generated.

BHK-21 were transfected to express the Ebola-GP protein using Lipofectamine 3000 (Thermo Fisher Scientific, Madrid, Spain), and after 24 h, cells were inoculated with a replication-deficient rVSV-luc pseudotype (MOI: 3–5) that contains firefly luciferase instead of the VSV-G open reading frame, rVSVΔG-luciferase (G*ΔG-luciferase; Kerafast). After 1 h incubation at 37 °C, the inoculum was removed, cells were washed intensively with PBS, and then the medium was added. Pseudotyped particles were harvested 20 h postinoculation, clarified from cellular debris by centrifugation, and stored at −80 °C. Infectious titers were estimated as tissue culture infectious dose per mL by limiting dilution of the Ebola-GP rVSV-luc-containing supernatants on VeroE6 cells. Luciferase activity was determined by luciferase assay (Steady-Glo Luciferase Assay System, Promega) in a GloMax Navigator Microplate Luminometer (Promega).

#### Screening of Selected Compounds

All the compounds tested in this work were initially resuspended in DMSO at 1 mM.

Screening of selected compounds as EBOV-GP-pseudotyped virus entry inhibitors was performed using VeroE6 cells (2 × 10^4^ cells/well) in 96-well plates.

VeroE6 cells were incubated at 37 °C for 1 h with the compounds and then challenged with 5000 TCID (Tissue Culture Infective Dose) of recombinant viruses. After 24 h of incubation, cells were washed with PBS, lysed by addition of Steady-Glo Lysis Buffer (Promega), and light-measured in a GloMax Navigator Microplate Luminometer (Promega).

Compounds that inhibited virus infection by more than 75% at a final concentration of 10 μM were further analyzed for potency, selectivity, and cytotoxicity. For these compounds, the range of concentrations tested was 10 nM to 10 μM. As a control for selectivity, infection with VSV-G pseudoviruses was performed in the same conditions (Table S1 of the Supporting Information).

#### Toxicity Analysis of Compounds

VeroE6 (2 × 10^4^) cells were seeded in a 96-well plate and incubated with DMEM containing each compound at concentrations ranging from 0 to 200 μM. After 24 h, cell viability was measured by CellTiter-Glo Luminescent Cell Viability Assay (Promega).

Cell viability was reported as the percentage of luminescence in treated cells relative to nontreated cells.

CC_50_ was calculated and nontoxic working concentrations (over 80% cell viability) were used to test the activities of these compounds on EBOV-GP-pseudotyped infection.

#### Statistical Analysis

The values of EC_50_ inhibition of the infection presented on the table correspond to the mean of 3 independent experiments. The EC_50_ values were estimated using GraphPad Prism v6.0 with a 95% confidence interval and settings for normalized dose–response curves.

#### Effect of the Ebola GP Y517S or F630H/W Mutations on the Inhibitory Capacity of Selected Compounds

293T cells were infected with Ebola wt pseudotypes or with the mutants Y517S–F630H/W in the presence of selected compounds (10 μM) previously incubated at 37 °C for 1 h with these cells. Forty-eight hours later, cells were lysed and light-measured.

As control compounds, imipramine and toremifene at 1 μM were used for Y517S mutant and fluoxetine (12 μM) for F630H/W mutant.

### Antiviral Activity in Wild-Type Zaire EBOV Mayinga Virus

VeroE6 cells (1 × 10^5^ cells per well in a 24-well plate) were infected with EBOV (Mayinga variant) at a multiplicity of infection (MOI) of 0.1. One hour later, the supernatant containing any unbound virus was discarded and the cells were washed once with 500 μL of PBS. During the incubation period, the compounds (initially resuspended in DMSO at 10 mM) were prepared and added to DMEM supplemented with 5% FBS and methyl cellulose. The concentration of the compounds ranged from 0.1 to 50 μM. After addition of the compounds to the cells, they were incubated at 5% CO_2_ and 37 °C for 3 days. The concentration in the cell culture supernatant of infectious virus particles was then measured using an immunofocus assay as follows. The supernatant was discarded and the cells were fixed in 4% paraformaldehyde for 1 h. The plates were washed thoroughly after fixation and in between each step thereafter. The cells were permeabilized with 0.5% Triton X-100 in PBS for 30 min followed by blocking with a solution of 5% FBS in PBS for 1 h. The primary antibody, polyclonal mouse anti-EBOV antibody (1:5000 in 2.5% blocking solution), was added followed by overnight incubation. The secondary antibody, peroxidase-conjugated sheep antimouse IgG (H+L) (1:5000 in 5% blocking solution), was added followed by 1 h of incubation. To detect foci, tetramethylbenzidine (1:3 in distilled water) was added for 30 min or until spots developed after which it was discarded and the foci counted.

The concentrations that reduced virus titer by 50% (EC_50_) were calculated from dose–response curves using GraphPad Prism 9 with a 95% confidence interval.

### Computational Protocol

Extended molecular dynamics (MD) simulations were employed to examine the structural and dynamic features of the simulated system. To achieve this, the GP system was constructed using the protein bound to toremifene (PDB: 5JQ7).^22^ The binding mode of thiophene derivatives **1**, **53**, and **57** was determined through glide docking of each compound onto the minimized X-ray structure. The grid box was positioned at the toremifene binding site, utilizing default parameters for receptor grid generation.^43^ Acetyl (ACE) and *N*-methyl (NME) capping was introduced to neutralize both the N- and C-termini of the protein. A sum of 15 disulfide connections (C53_A-C_-C609_A-C_, C108_A-C_-C135_A-C_, C121_A-C_- C147_A-C_, C511_A-C_-C556_A-C_, and C601_A-C_-C609_A-C_) were specified to form the functional homotrimer.

The simulated systems were immersed within a pre-equilibrated octahedral box of TIP3P water molecules.^51^ Ionizable residues were assigned their standard protonation state at physiological pH. The resultant systems comprised a model protein, encompassing approximately 21,000 water molecules, and 3 Na cations, totaling around 80,000 atoms. Simulations employed the NPT ensemble for equilibration and the NVT ensemble for production runs, incorporating periodic boundary conditions and Ewald sums with a grid spacing of 1 Å to handle long-range electrostatic interactions. All simulations adhered to the Amber ff14SB force field^52^ for protein and were executed using Amber20.^53^ The ligands were parametrized using the GAFF force field^54^ in conjunction with restrained electrostatic potential-fitted (RESP)^55^ partial atomic charges derived from B3LYP/6-31G(d)^56^ calculations.

The initial system underwent minimization through a multistep protocol. Initially, the positions of all hydrogen atoms in the protein underwent refinement via energy minimization (2000 cycles of steepest descent + 8000 cycles of conjugate gradient). Subsequently, this approach was extended to minimize the positions of water molecules and counterions. Finally, all atoms within the system underwent energy minimization (4000 cycles for steepest descent + 1000 cycles of conjugate gradient). The equilibration process encompassed six steps. The system was initially heated from 0 to 100 K in 20 ps (NVT ensemble), followed by four thermalization steps to elevate the temperature from 100 to 300 K (50 ps/step, NPT conditions). A concluding 5 ns step was conducted to equilibrate the system’s density at a constant temperature (300 K) and pressure (1 atm). The resulting structure from the equilibration process served as the initial conformation for the MD simulations. Throughout the equilibration steps, various distance constraints were employed to stabilize the ligand’s position, preventing any artifactual movements in the initial stages. Subsequently, these constraints were gradually reduced and ultimately eliminated in the early steps of MD simulations.^57,58^ Three replicas of the protein–ligand complexes were simulated for every thiophene derivative, each simulation lasting 500 ns.

For the analysis, the hbond, rmsd, and rmsf commands of the CPPTRAJ20 module were used to evaluate the stability of the protein and relevant interactions established between the ligand and the protein.^59^

#### Essential Dynamics

This approach was employed to identify the principal motions derived from the structural variations sampled in MD simulations. In essential dynamics (ED) analysis, the dynamics along individual modes are examined and visualized separately, enabling the extraction of the predominant collective motions during the simulations. To accomplish this, a positional covariance matrix is generated and diagonalized to obtain the collective deformation modes, known as eigenvectors, while the associated eigenvalues represent the contribution of each motion to the overall structural variance of the protein. For our study, ED analysis was conducted using 25,000 snapshots extracted from 500 ns of each simulation, focusing only on the backbone atoms. The computations were performed utilizing the PCAsuite program, which is accessible at http://mmb.irbbarcelona.org/software/pcasuite/pcasuite.html and integrated into the pyPCcazip program, a suite of tools.^46^

#### MM-GBSA Calculations

The main objective of the MM-GBSA^44^ technique is to compute the difference in free energy between two conditions, typically representing the bound and unbound states of two solvated molecules. Alternatively, it can be used to compare the free energy of two distinct solvated conformations of a single molecule. In our investigation, we employed MM/PB(GB)SA scripts integrated with Amber and AmberTools to automate all required procedures for estimating the binding free energy of our protein–ligand complex using the MM-GBSA method.

### Inhibitory Effect of Compounds on EBOV-GPcl/NPC1-domain C Interaction

#### Cleaved EBOV-GP (EBOV-GPcl)

Cleaved EBOV-GP (EBOV-GPcl) was generated *in vitro* using the bacterial protease thermolysin (250 μg/mL) (Sigma-Aldrich, St. Louis, MO) for 1 h at 37 °C and the reaction was stopped by adding the metalloprotease inhibitor phosphoramidon (1 mM) (Sigma-Aldrich) for 20 min on ice.

#### NPC1-domain C Construct (Plasmid)

A cassette vector based on *Homo sapiens* NPC1-mRNA NM-000271 encoding the following sequence elements was synthesized on a pcDNA3 plasmid: signal peptide (residues 1–24), domain C (residues 373–620), the first transmembrane domain (residues 267–295), Gly-Gly-Gly-Ser linker, and a triple Flag tag GeneArt (Thermo Fisher).

#### Expression, Purification, and Detection of NPC1-domain C-Flag Fusion Protein

HEK293T cells (ATCC–CRL-11268) were transfected using Lipofectamine 3000 (Thermo Fisher) with the plasmid encoding NPC1-domain C-Flag. Thirty-six hours post transfection, cells were washed, lysed, and collected (Cell Lytic M-C2978, Sigma-Aldrich).

Proteins from the cell lysate were purified by affinity chromatography using an anti-Flag-M2 agarose column according to the manufacturer’s instructions (Sigma-Aldrich).

Detection of NPC1-domain C-Flag protein was performed by western blot using an Anti-Flag M2- Peroxydase (1:1000) monoclonal antibody (Sigma-Aldrich).

#### EbolaGP-NPC1 Domain C Binding ELISAs

NPC1-domain C concentrations used in the ELISAs were normalized using Micro BCA protein assay kit (Thermo).

Thermolysin-cleaved HIV-EBOV GP particles were captured onto high-binding 96-well ELISA plates (Corning, Corning, NY) using a conformation-specific anti-EBOV GP monoclonal antibody KZ52 (6.23 μg/mL).

Unbound viral particles were washed off, and purified Flag-tagged soluble NPC1-domain C (10 μg/mL) was added in the presence or not (control) of each compound (50 μM).

After that, bound flag-tagged proteins were detected with an anti-Flag antibody covalently conjugated to horseradish peroxidase (HRP) (1:5000) (Sigma-Aldrich). Finally, absorbance at 450 nm was measured after addition of the TMB substrate.

### Liver Microsome Stability Assay

Mouse or human liver microsomes and reduced nicotinamide adenine dinucleotide phosphate (NADPH) were obtained from Fisher Scientific SL. This assay gives information on the metabolic stability of early drug discovery compounds based on liver microsomes. Microsome stability was tested by incubating 10 μM of test compound (**57**) and verapamil (as positive metabolized control) with 1.0 mg/mL hepatic microsomes (pooled human liver microsomes and pooled mouse (CD-1) liver microsomes) in 0.1 M potassium phosphate buffer (pH 7.4) with 5 mM MgCl_2_. The reaction was initiated by adding NADPH (1 mM final concentration). Aliquots (150 μL) were collected at defined time points (0, 5, 15, 30, 45, and 60 min) and added to cold acetonitrile (150 μL) containing an internal standard (5 μg/mL warfarin) to stop the reaction and precipitate the protein. After stopping the reaction, the samples were centrifuged at room temperature for 15 min and the loss of parent compounds was analyzed by HPLC-MS using single ion mode (SIM) detection. Data were log-transformed and represented as half-life. All experiments were conducted by duplicate.

### Assessment of hERG Activity

hERG potassium channel inhibition assay was carried out in hERG-expressed HEK293 cells using the FluxOR potassium assay and performed on a FLIPR TETRA (Molecular Devices) as outlined in the product information sheet from Invitrogen. As directed by the kit, the Powerload concentrate and water-soluble probenecid were added in the first step to enhance the dye solubility and retention, respectively. Then, FluxOR dye was added and mixed. The FluxOR loading buffer (165 mM NaCl, 4.5 mM KCl, 2 mM CaCl_2_, 1 mM MgCl, 10 mM HEPES, and 10 mM glucose) was adjusted to a pH of 7.2.

Media were removed from cell plates and 50 μL of loading buffer containing the FluxOR dye mix was applied to each. The dye was removed after 60 min incubation at room temperature and the plates subsequently washed once with assay buffer before adding the samples in assay buffer (final volume of 50 μL). Plates were incubated for 30 min at room temperature (25 °C) to allow equilibration of the test compounds. The thallium stimulation buffer (Tl_2_SO_4_ + K_2_SO_4_) was prepared according to the manufacturer’s instruction and injected into the plates on the FLIPR TETRA to allow kinetic analysis from time zero (*t*_0_) to time 120 s (*t*_120_). Data obtained were analyzed using Genedata Screener.

The compounds were tested in triplicate using 10 points/1:2 dilution dose–response curves with maximum concentrations at 50 μM. Astemizole was used as positive control and 0.5% DMSO as negative control.

### Assessment of Nav1.5 Activity

The measurement is performed with FLIPR Membrane Potential Assay dye, detecting changes in membrane potential brought about by compounds that modulate or block voltage-gated Na+ channels, through FLIPR Tetra High-Throughput Cellular Screening System (Molecular Devices). The cell model used for the assay is Nav1.5-HEK293 cell line that stably expresses at passage 20.

Compounds were prepared in DMSO at 10 mM and were tested at the highest concentration of 50 μM (10 points, 1:2 dilution) per triplicate and IC_50_ were calculated. Positive control (50 μM tetrodotoxin) and negative control (0.5% DMSO) were introduced in the plate. The dose–response curve with tetrodotoxin is assayed as standard. Veratridine is used to hold the sodium channel in its open state, preventing inactivation through binding to site two of the six topologically separated toxin binding sites that have been described.^60^ Rapid influx of Na+ into the cell subsequently depolarizes the membrane, leading to an increase in fluorescence. Tetrodotoxin (positive control) is a potent sodium channel blocker isolated from Japanese puffer fish. A 10× dose–response series of positive control, negative control (vehicle), and sample are added to the cells 15 min prior to addition of veratridine.

### *In Vivo* Pharmacokinetic Studies

The study was conducted at the AAALAC-accredited facility of Sai Life Sciences Ltd. in Hyderabad, India, following the Study Protocols SAIDMPK/PK-21-06-583 and SAIDMPK/PK-21-12-1219 for compounds **1** and **57**, respectively. All procedures adhered to the guidelines provided by the Committee for the Purpose of Control and Supervision of Experiments on Animals (CPCSEA) as published in The Gazette of India, December 15, 1998. Prior approval from the Institutional Animal Ethics Committee (IAEC) was obtained before the initiation of the studies.

Healthy male BALB/c mice (8–12 weeks old) weighing between 20 and 35 g were used in the study. A total of 48 male mice were divided into two groups: group 1 (*n* = 24) and group 2 (*n* = 24), employing a three-mice-per-time-point design. Animals in group 1 received intraperitoneal (i.p.) administration of the test compound solution at a dose of 10 mg/kg, while animals in group 2 were administered via the oral route (p.o.) with the test compound solution at a dose of 50 mg/kg. The dosing volume for both i.p. and p.o. administrations was 10 mL/kg. For the investigation of the pharmacokinetics of thiophene **1**, the formulation comprised 5% *N*-methyl-2-pyrrolidone (NMP), 5% solutol HS-15, 30% PEG-400, and 60% normal saline. For thiophene **57**, the formulation consisted of 5% *N*-methyl-2-pyrrolidone (NMP), 5% solutol HS-15, 30% PEG-400, and 60% captisol (20% w/v).

Blood samples (approximately 60 μL) were collected from a set of three mice at each time point (0.08 [for i.p. only], 0.25, 0.5, 1, 2, 4, 6 [for p.o. only], 8, and 24 h). Additionally, along with terminal blood samples, brain samples were collected at 0.08 (for i.p only), 0.25, 0.5, 1, 2, 4, 6 (for p.o. only), 8, and 24 h postdosing from three mice per time point. Immediately after blood collecting, brain samples were collected from the set of three animals for bioanalysis. The concentrations of the compound in mouse plasma and brain samples were determined by a fit-for-purpose LC–MS/MS method. The noncompartmental analysis tool of Phoenix WinNonlin (ver. 8.0) was employed to assess the pharmacokinetic parameters.

### Single-Dose Acute Tolerability Study

The study was conducted at the Laboratory Animal House facility of Sai Life Sciences Ltd. in Telagana, India, following the Study Protocol Nr TOX-359. All procedures were in accordance with the guidelines provided by the Committee for Control and Supervision of Experiments on Animals (CCSEA) as published in The Gazette of India, December 15, 1998. Prior approval from the Institutional Animal Ethics Committee (IAEC) was obtained before the initiation of the study.

The study design comprised of four groups of C57BL/6 mice including one control (G1) and three groups each treated with formulated thiophene **57** [G2 (50 mg/kg), G3 (100 mg/kg), and G4 (250 mg/kg)] having three mice/sex/group. The formulation consisted of 7.5% v/v NMP, 7.5% v/v Solutol HS-15, 25% v/v PEG-400, and 60% v/v RO Water. Animals from toxicity groups were administered test item formulations as single dose by oral (gavage) route, with a 4 day postdose observation period to determine maximum tolerable dose. Animals from the control group (G1) received vehicle. The dosing volume was kept constant at 10 mL/kg for each mouse.

Parameters evaluated during the study included in-life observations such as clinical signs observation, body weights, percent body weight gains, and feed consumption. After completion of the observation period of 4 days, the surviving animals were euthanized by CO_2_ asphyxiation on day 5. All the animals were subjected to detailed gross pathological examination.

## Supporting Information Available

Table S1: antiviral activity of selected derivatives against VSV-G-pseudotyped virus (pVSV-G); Table S2: free energy of ligand binding to the protein as determined using the MM/GBSA method; Figure S1: RMSD values (in Å) over the 500 ns simulations for three independent replicas of GP-**57**, GP-**53**, and GP-**1** complexes; Figures S2–S4: representation of per-replica mean fraction of hydrogen-bond interactions and π–π stacking distance distribution between the Y517_GP2_ and thiophene ring of compounds **57**, **53**, and **1**, respectively; Figures S5 and S6: HPLC chromatograms of compounds **1** and **57**; Figures S7 and S8: ^1^H NMR and ^13^C NMR spectra of compounds **1** and **57**; Chemical procedures to obtain 5-phenylthiophene-2-carboxamide derivatives **2–8** and bromo-heterocycle derivatives **9–15** and **65–72** following general procedure A; Chemical procedures to obtain hidroxyphenyl-heterocycle derivatives **16–31** and **83–89** following general procedure B; Chemical procedures to obtain protected-heterocycle derivatives **32–46**, **61**, **64**, and **90–96** following general procedure C; chemical procedures to obtain protected-heterocycle derivatives **62** and **73–77** following general procedure D (PDF)
Molecular formula string (CSV)
Representative binding mode of compound **1** (PDB)
Representative binding mode of compound **53** (PDB)
Representative binding mode of compound **57** (PDB)

## Supplementary Material

jm4c01267_si_002.csv

jm4c01267_si_003.pdb

jm4c01267_si_004.pdb

jm4c01267_si_005.pdb

## Abbreviations

ACN: acetonitrile
AUC_last_: area under the plasma concentration-time curve from time zero to the time of the last quantifiable concentration
BOMV: *Orthoebolavirus bombaliense*
bs: binding site
BSL-2: biosafety level 2
BSL-4: biosafety level 4
BUDV: *Orthoebolavirus bundibugyense*
B3LYP: Becke, 3-parameter, Lee–Yang–Parr
CI: confidence intervals
CL_int_: intrinsic clearance
CC_50_: 50% cytotoxic concentration
*C*_max_: peak serum concentration
DEAD: diethyl azodicarboxylate
DIAD: diisopropyl azodicarboxylate
DMSO: dimethyl sulfoxide
EBOV: *Orthoebolavirus zairense*
EBOV-GP: Ebola Virus Glycoprotein
EBOV May: Zaire EBOV Mayinga 1976 strain
EC_50_: 50% efective concentration
ED: essential dynamics
EDCl: 1-ethyl-3-(3-dimethylaminopropyl)carbodiimide hydrochloride
ELISA: enzyme-linked immunosorbent assay
EVD: Ebola virus disease
GAFF: general Amber force field
GP: glycoprotein
GPcl: cleaved glycoprotein
hERG: human ether-a-go-go-related gene
HOBt: 1-hydroxybenzotriazole
HR: heptad repeat region 2
IC_50_: 50% inhibitory concentration
i.p.: intraperitoneal
*K*_p_: brain/plasma ratio
*L*: RNA-dependent RNA polymerase
MD: molecular dynamics
MW: microwave irradiation
NMP: *N*-methyl-2-pyrrolidone
NP: nucleoprotein
NPC1: Niemann–Pick C1 receptor
NVT: canonical ensemble with constant number of particles, volume, and temperature
pEBOV: EBOV-GP-pseudotyped virus
p.o.: oral
RBS: receptor binding site
RESP: restrained electrostatic potential
RESTV: *Orthoebolavirus restoniense*
RMSD: root-mean-square deviation
RMSF: root-mean-square fluctuation
rVSV: recombinant vesicular stomatitis virus
SAR: structure–activity relationships
SI: selectivity index
SUDV: *Orthoebolavirus sudanense*
TAFV: *Orthoebolavirus taieense*
TFA: trifluoroacetic acid
THF: tetrahydrofuran
TIP3P: transferable intermolecular potential with 3 point
*T*_max_: time to reach *C*_max_
*t*_1/2_: half-life time
VP24, VP30, VP35, and VP40: viral proteins 24, 30, 35, and 40
VSV: vesicular stomatitis virus
VSV-G: vesicular stomatitis virus envelope GP
wt: wild-type
