# Supplementary material for: Discovery of Thiophene Derivatives as Potent, Orally Bioavailable, and Blood–Brain Barrier-Permeable Ebola Virus Entry Inhibitors
Source: J Med Chem. 2024 Sep 9;67(18):16381–402. doi: 10.1021/acs.jmedchem.4c01267 (PMC11440591; doi:10.1021/acs.jmedchem.4c01267)
Supplement: Supplementary file 1 — jm4c01267_si_001.pdf [file jm4c01267_si_001.pdf]

SUPPORTING INFORMATION

DISCOVERY OF THIOPHENE DERIVATIVES AS POTENT EBOLA VIRUS ENTRY INHIBITORS ORALLY BIOAVAILABLE AND BLOOD-BRAIN BARRIER PERMEABLE

Marcos Morales-Tenorio,[1,†] Fátima Lasala,[2, †] Alfonso Garcia-Rubia,[1] Elnaz Aledavood,[1] Michelle Heung,[3] Catherine Olal,[3] Beatriz Escudero-Pérez,[3] Covadonga Alonso,[4] Ana Martínez,[1,5] César Muñoz-Fontela,[3] Rafael Delgado,[2,6,7,*] Carmen Gil[1,5,*]

[1]Centro de Investigaciones Biológicas Margarita Salas (CIB-CSIC), 28040 Madrid, Spain

[2]Instituto de Investigación Hospital 12 de Octubre, 28041 Madrid, Spain

[3]Bernhard Nocht Institute for Tropical Medicine, 20359 Hamburg, Germany

[4]Dpt. Biotechnology, Instituto Nacional de Investigación y Tecnología Agraria y Alimentaria (INIA-CSIC), 28040 Madrid, Spain

[5]CIBERNED, Instituto Salud Carlos III, 28029 Madrid, Spain

[6]CIBERINFEC, Instituto Salud Carlos III, 28029 Madrid, Spain

[7]School of Medicine, Universidad Complutense de Madrid, 28040, Madrid, Spain

Table of content

- Page S2: Table S1. Antiviral activity of selected derivatives against VSV-G-pseudotype virus (pVSV-G).
- Page S3: Table S2. The free energy of ligand binding to the protein as determined using the MM/GBSA method.
- Page S4: Figure S1. RMSD values (in Å) over the 500 ns of simulations for three independent replicas of GP-57, GP-53 and GP-1 complexes.
- Page S5-S7: Figures S2, S3 and S4. Representation of per-replica mean fraction of hydrogen-bond interactions and π-π stacking distance distribution between the $Y517_{GP2}$ and thiophene ring of compounds **57**, **53** and **1** respectively.
- Page S8: HPLC chromatograms of compounds **1** and **57**.
- Page S9: ^{1}H NMR and ^{13}C NMR spectra of compound **1**.
- Page S10: ^{1}H NMR and ^{13}C NMR spectra of compound **57**.
- Page S11: Chemical procedures for the synthesis of 5-phenylthiophene-2-carboxamide derivatives **2-8** and bromo-heterocycle derivatives **9-15** and **65-72**.
- Page S16: Chemical procedures for the synthesis of hidroxyphenyl-heterocycle derivatives **16-31** and **83-89**.
- Page S23: Chemical procedures for the synthesis of protected-heterocycle derivatives **32-46**, **61**, **64** and **90-96**.
- Page S30: Chemical procedures for the synthesis of protected-heterocycle derivatives **62** and **73-77**.

Table S1. Antiviral activity of selected derivatives against VSV-G-pseudotype virus (pVSV-G).

	%inh@10μM (pVSV-G)
**1**	0.66%@10μM
**47**	-117%@10μM
**48**	-227%@10μM
**49**	-160%@10μM
**50**	-31%@10μM
**51**	-148%@10μM
**52**	-108%@10μM
**53**	7%@10μM
**54**	30%@10μM
**55**	-4%@10μM
**56**	-182%@10μM
**57**	24%@10μM
**58**	-12%@10μM
**59**	5%@10μM
**60**	-12%@10μM
**63**	12%@10μM
**78**	-32%@10μM
**79**	39%@10μM
**80**	30%@10μM
**81**	30%@10μM
**82**	-62%@10μM
**97**	18%@10μM
**98**	-18%@10μM
**99**	10%@10μM
**100**	32%@10μM
**101**	39%@10μM
**102**	-23%@10μM
**103**	-18%@10μM

Table S2. The free energy of ligand binding to the protein as determined using the MM/GBSA method

Comp.	MM/GBSA (kcal/mol)
**Toremifene**	-43.4 ± 3.4
**57**	-39.1 ± 4.0
**53**	-35.8 ± 4.1
**1**	-29.3 ± 4.5

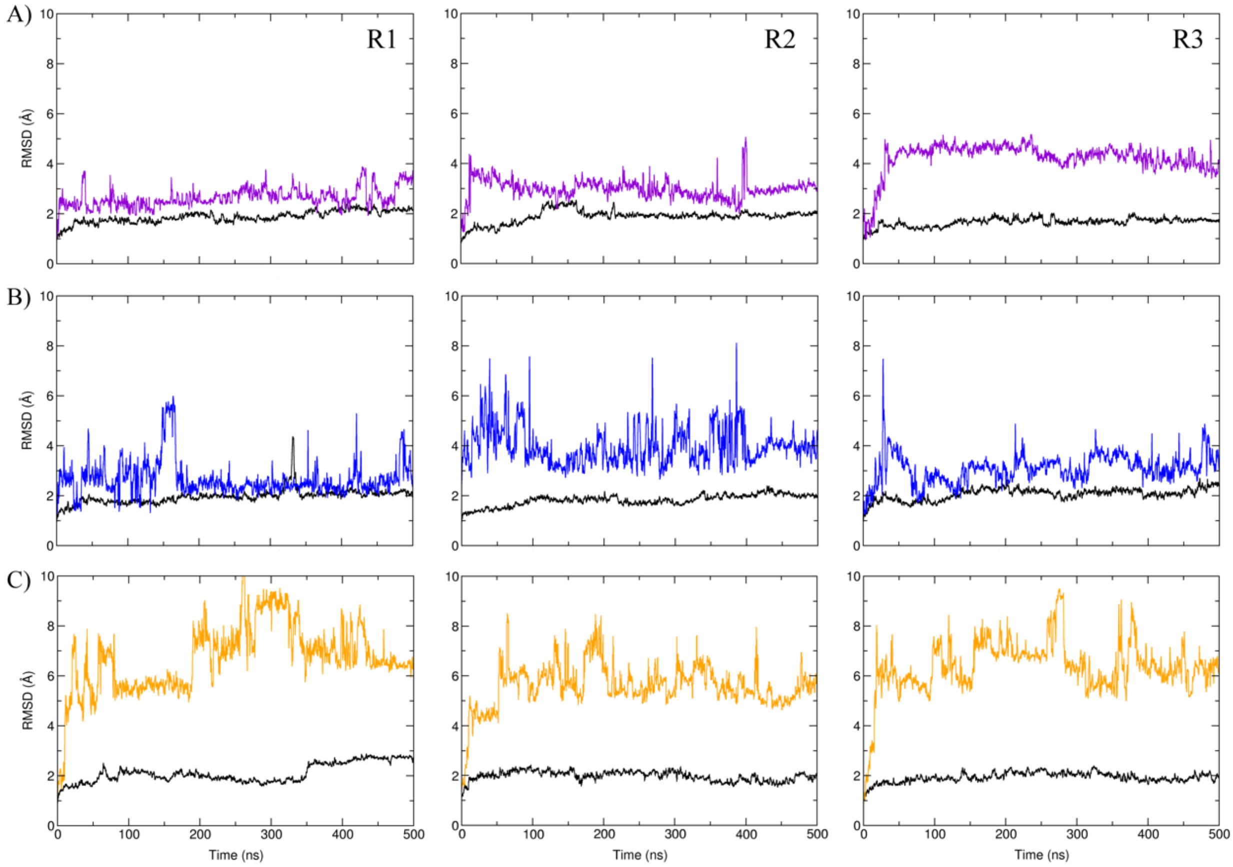

Figure S1. RMSD values (in Å) over the 500 ns of simulations for three independent replicas of A) GP-**57**, B) GP-**53** and C) GP-**1** complexes with protein backbone atoms indicated in black and the ligand highlighted in violet, blue and orange, respectively.

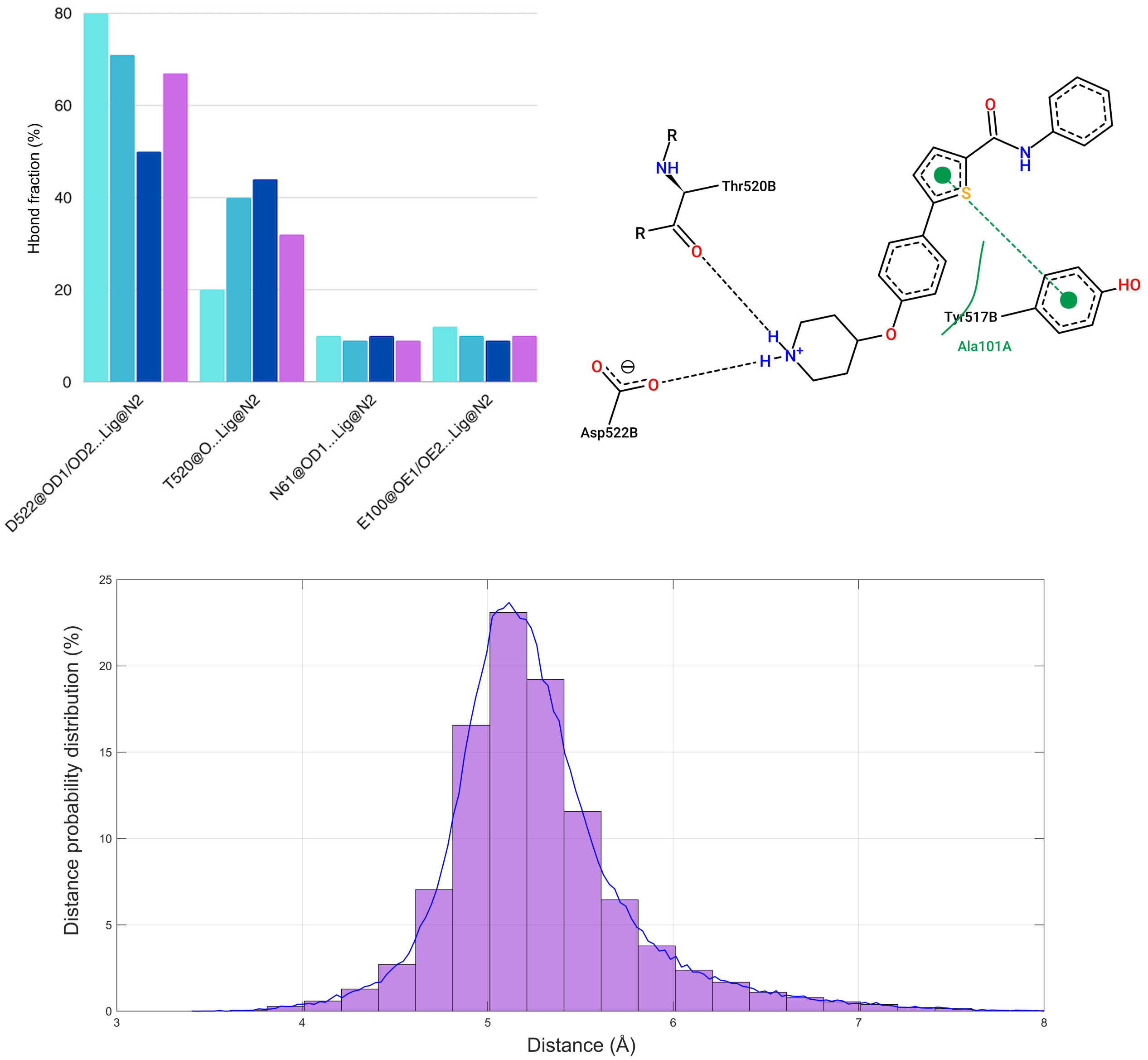

Figure S2. Representation of per-replica mean fraction of hydrogen-bond interactions (depicted in shades of blue, alongside the global mean fraction highlighted in pink) and π-π stacking distance distribution between the $Y517_{GP2}$ and thiophene ring of GP-**57** complexes.

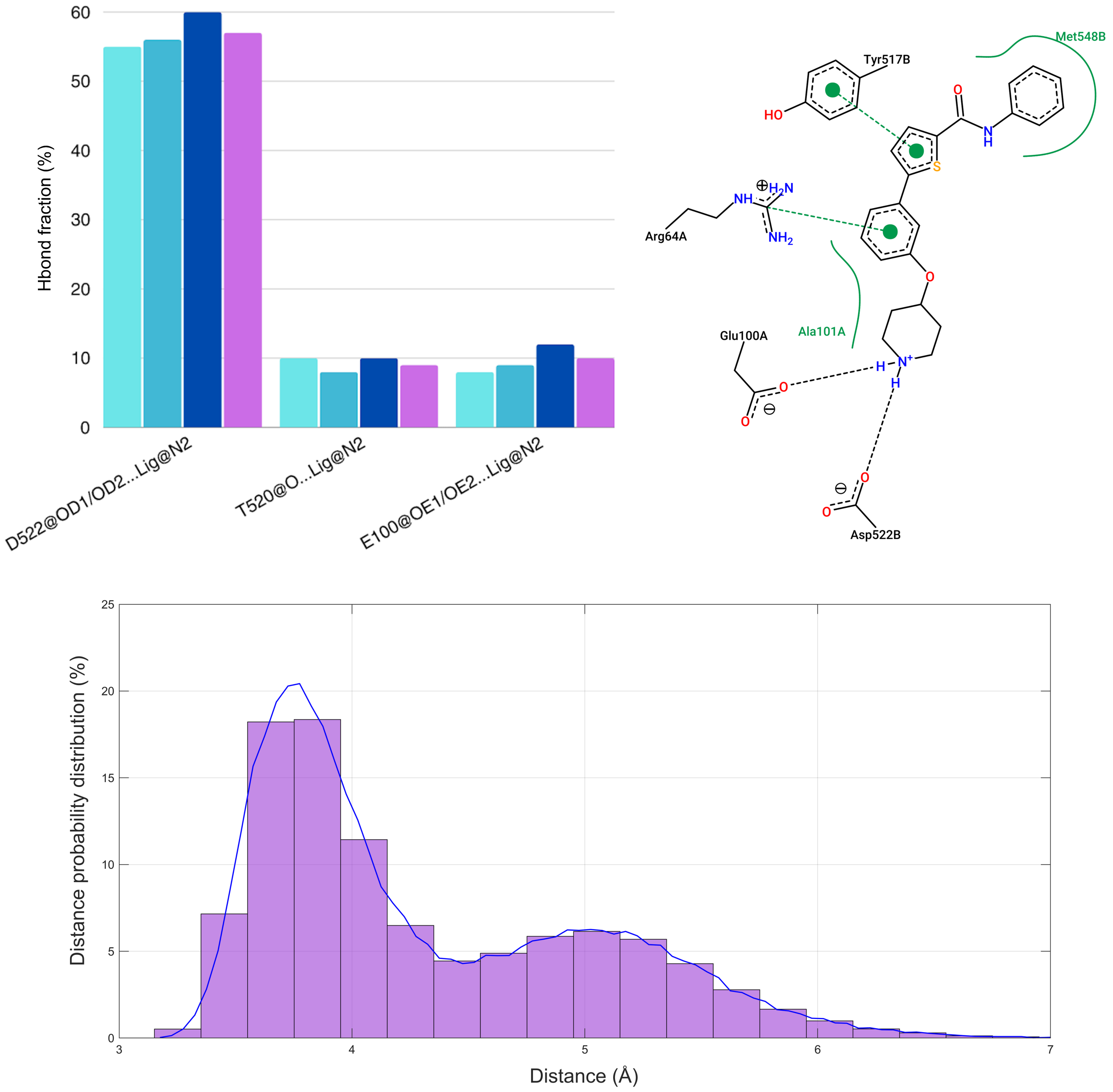

Figure S3. Representation of per-replica mean fraction of hydrogen-bond interactions (depicted in shades of blue, alongside the global mean fraction highlighted in pink) and π-π stacking distance distribution between the Y517$_{GP2}$ and thiophene ring of GP-**53** complexes.

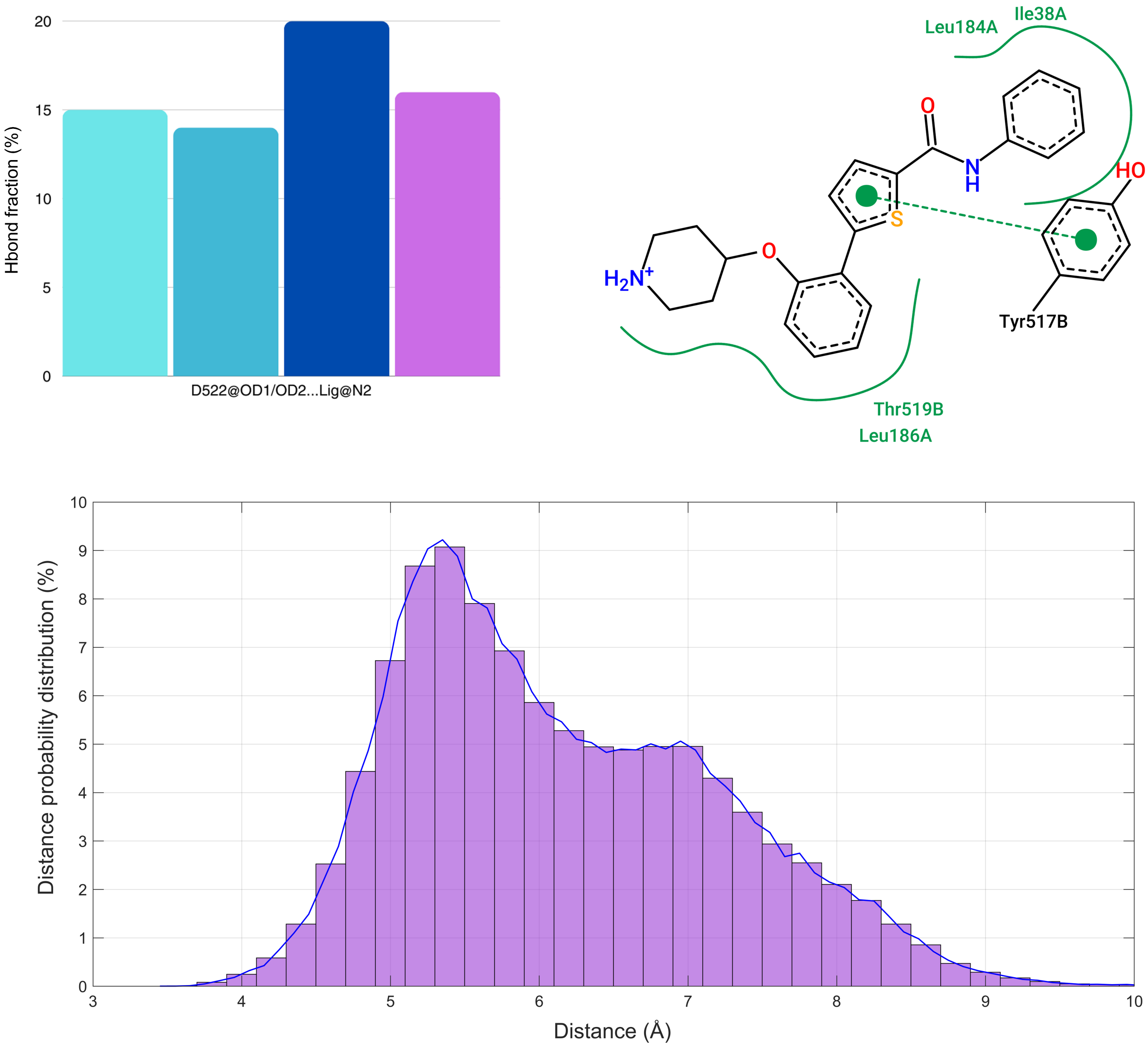

Figure S4. Representation of per-replica mean fraction of hydrogen-bond interactions (depicted in shades of blue, alongside the global mean fraction highlighted in pink) and π-π stacking distance distribution between the $Y517_{GP2}$ and thiophene ring of GP-**1** complexes.

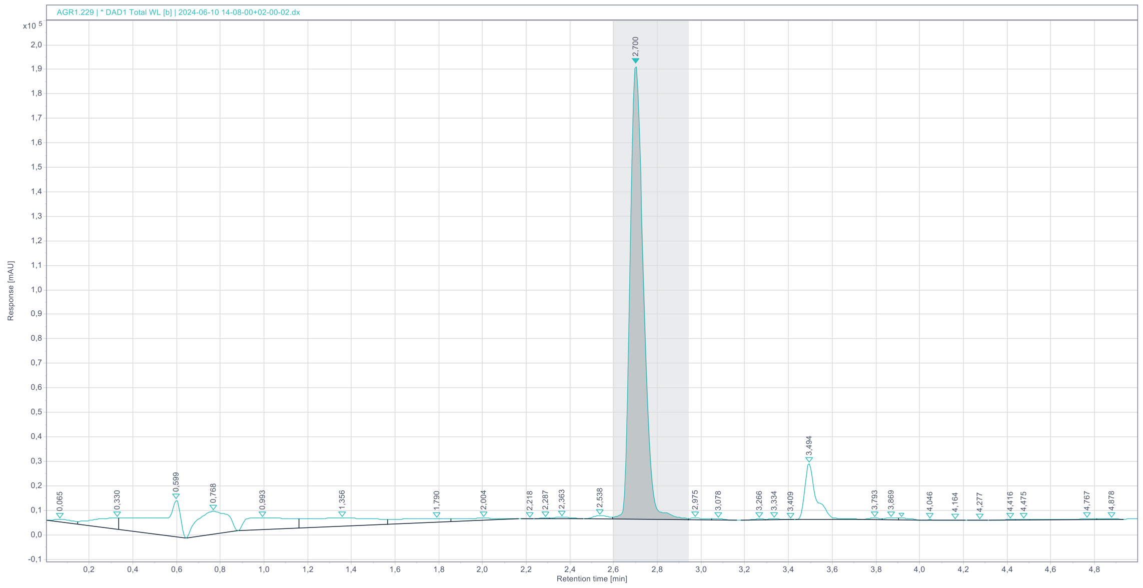

Figure S5. HPLC chromatogram of compound **1**.

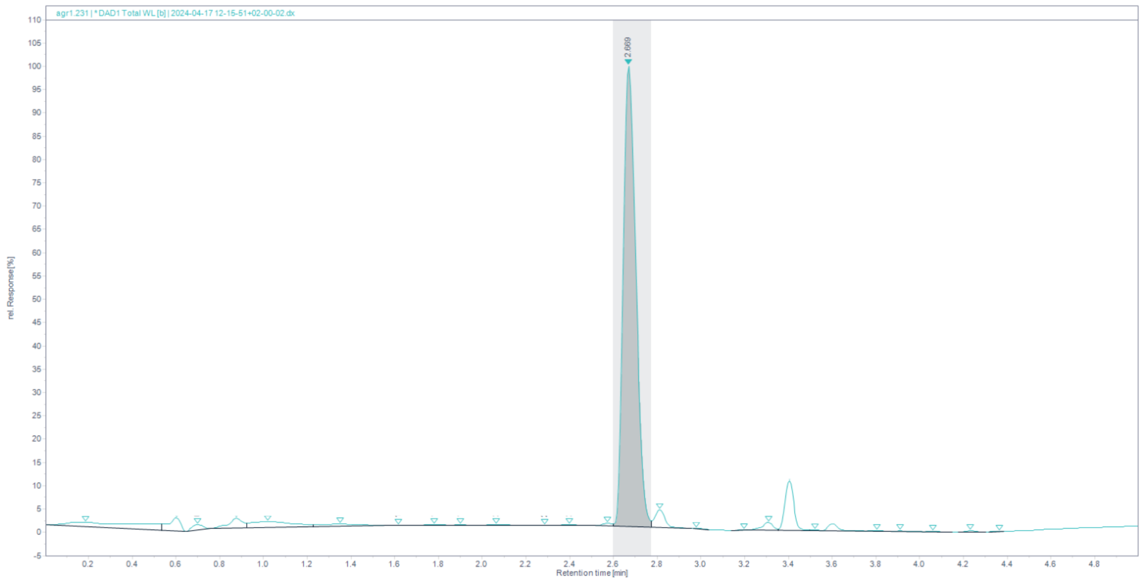

Figure S6. HPLC chromatogram of compound **57**.

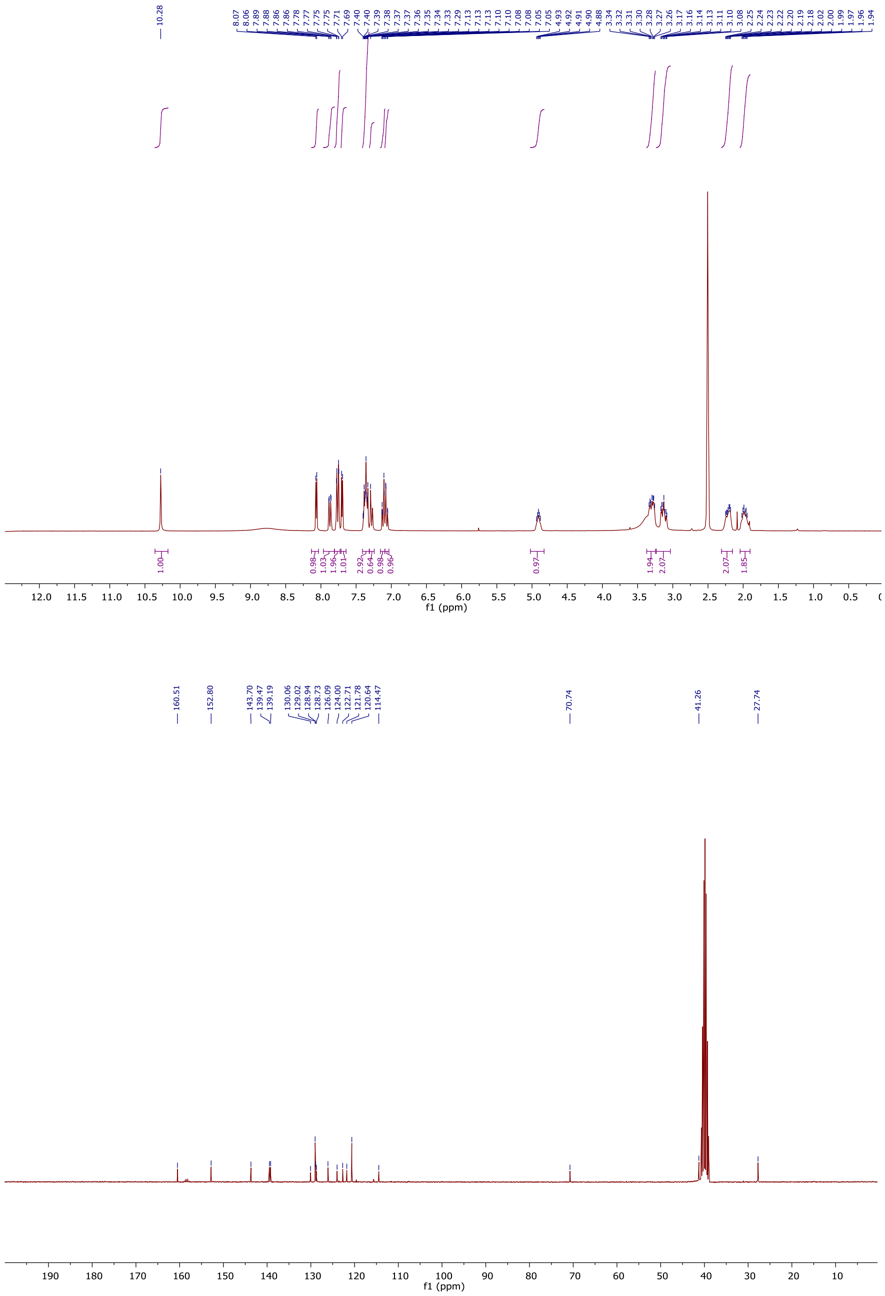

Figure S7. ^{1}H NMR and ^{13}C NMR spectra of compound **1**.

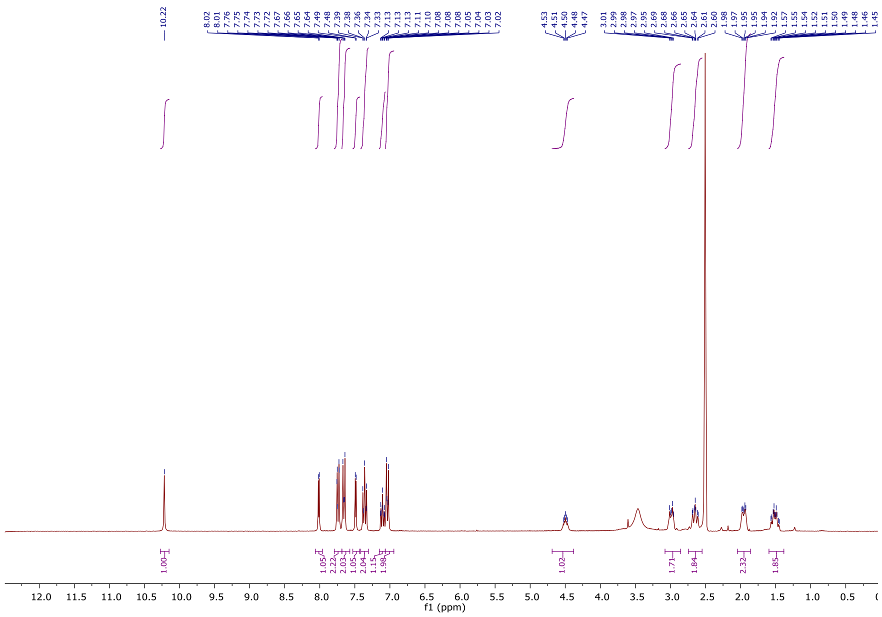

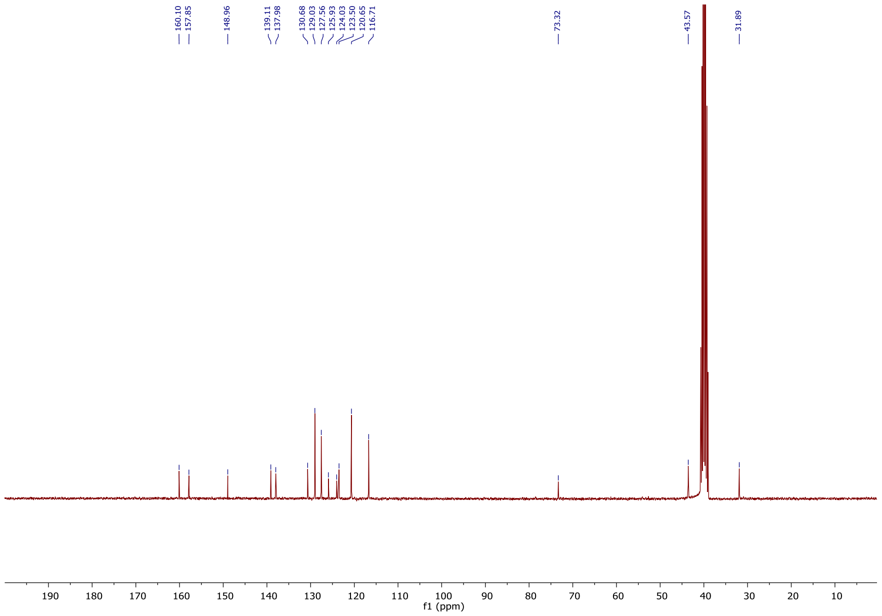

Figure S8. ^{1}H NMR and ^{13}C NMR spectra of compound **57**.

Chemical procedures for the synthesis of 5-phenylthiophene-2-carboxamide derivatives 2-8 and bromo-heterocycle derivatives 9-15 and 65-72 following general procedure A

N,5-Diphenylthiophene-2-carboxamide (**2**). The title compound was prepared by reaction of 5-phenylthiophene-2-carboxylic acid (0.71 mmol, 144 mg), aniline (0.72 mmol, 65 µL), EDCl (1.41 mmol, 269 mg), HOBt (1.41 mmol, 190 mg), Et_3N (2.12 mmol, 294 µL) and CH_2Cl_2 as solvent (6 mL) according to procedure A. Purification: Hex/EtOAc (9:1). Yield: 43 mg (25%) as a white solid. Mp 206 – 208 ºC. ^{1}H NMR (300 MHz, DMSO-d_6) δ 10.23 (s, 1H), 8.03 (d, J = 4.0 Hz, 1H), 7.80 – 7.70 (m, 4H), 7.63 (d, J = 4.0 Hz, 1H), 7.51 – 7.43 (m, 2H), 7.43 – 7.32 (m, 3H), 7.15 – 7.07 (m, 1H). ^{13}C RMN (75 MHz, DMSO-d_6) δ 159.6, 148.3, 138.8, 138.6, 132.9, 130.2, 129.2 (2C), 128.7 (3C), 125.7 (2C), 124.4, 123.7, 120.3 (2C). HRMS (ESI) calc. for $C_{17}H_{14}NOS$ $[M + H]^+$ 280.0791; found 280.0779. HPLC-MS (gradient I) $(M + H)^+$ = 280, R_t = 6.20 min (99%).

N-(4-Chlorophenyl)-5-phenylthiophene-2-carboxamide (**3**). The title compound was prepared by reaction of 5-phenylthiophene-2-carboxylic acid (1.5 mmol, 306 mg), 4-chloroaniline (1.53 mmol, 195 mg), EDCl (3 mmol, 573 mg), HOBt (3 mmol, 405 mg), Et_3N (4.5 mmol, 626 µL) and CH_2Cl_2 as solvent (6 mL) according to procedure A. Purification: CH_2Cl_2/MeOH (9:1). Yield: 67 mg (14%) as a white solid. Mp 227 – 229 ºC. ^{1}H RMN (300 MHz, DMSO-d_6) δ 10.37 (s, 1H), 8.03 (d, J = 3.9 Hz, 1H), 7.82 – 7.73 (m, 4H), 7.65 (d, J = 4.0 Hz, 1H), 7.52 – 7.48 (m, 1H), 7.48 – 7.44 (m, 2H), 7.44 – 7.37 (m, 2H). ^{13}C RMN (75 MHz, DMSO-d_6) δ 159.7, 148.6, 138.4, 137.7, 132.9, 130.5, 129.2 (2C), 128.7, 128.6 (2C), 127.3, 125.7 (2C), 124.4, 121.8 (2C). HRMS (ESI) calc. for $C_{17}H_{13}ClNOS$ $[M + H]^+$ 314.0401; found 314.0398. HPLC-MS (gradient I) $(M + H)^+$ = 314, R_t = 6.68 min (99%).

N-(3-Chlorophenyl)-5-phenylthiophene-2-carboxamide (**4**). The title compound was prepared by reaction of 5-phenylthiophene-2-carboxylic acid (1.5 mmol, 306 mg), 3-chloroaniline (1.53 mmol, 195 mg), EDCl (3 mmol, 573 mg), Et_3N (4.5 mmol, 626 µL) and CH_2Cl_2 as solvent (6 mL) according to procedure A. Purification: CH_2Cl_2/MeOH (9:1). Yield: 69 mg (15%) as a beige solid. Mp 155 – 157 ºC. ^{1}H RMN (300 MHz, DMSO-d_6) δ 10.39 (s, 1H), 8.04 (d, J = 4.0 Hz, 1H), 7.93 (t, J = 2.0 Hz, 1H), 7.73-7.81 (m, 2H), 7.69 (ddd, J = 8.3, 2.0, 1.0, 1H), 7.65 (d, J = 4.0 Hz, 1H), 7.51 – 7.37 (m, 4H), 7.18 (ddd, J = 8.0, 2.1, 0.9 Hz, 1H). ^{13}C RMN (75 MHz, DMSO-d_6) δ 159.9, 148.8, 140.1, 138.2, 132.9, 132.9, 130.6, 130.4, 129.3 (2C), 128.8, 125.8 (2C), 124.5, 123.4, 119.6, 118.5. HRMS (ESI) calc. for $C_{17}H_{13}ClNOS$ $[M + H]^+$ 314.0401; found 314.0400. HPLC-MS (gradient I) $(M + H)^+$ = 314, R_t = 6.76 min (98%).

N-Phenethyl-5-phenylthiophene-2-carboxamide (**5**). The title compound was prepared by reaction of 5-phenylthiophene-2-carboxylic acid (1.5 mmol, 306 mg), 2-phenethylamine (1.53 mmol, 185 mg), EDCl (3 mmol, 573 mg), Et_3N (4.5 mmol, 626 µL) and CH_2Cl_2 as solvent (6 mL) according to procedure A. Purification: CH_2Cl_2/MeOH (9:1). Yield: 115 mg (25%) as a white solid. Mp 170.5 – 172.5 ºC. ^{1}H RMN (300 MHz, DMSO-d_6) δ 8.64 (t, J = 5.6 Hz, 1H), 7.74 – 7.68 (m, 3H), 7.53 (d, J = 3.9 Hz, 1H), 7.49 – 7.41 (m, 2H), 7.41 – 7.34 (m, 1H), 7.34 – 7.17 (m, 5H), 3.53 – 3.43 (m, 2H), 2.85 (t, J = 7.5 Hz, 2H) ^{13}C RMN (75 MHz, DMSO-d_6) δ 160.9, 147.1, 139.3, 139.0, 133.1, 129.2 (2C), 128.9, 128.6 (2C), 128.4, 128.3 (2C),

126.1, 125.6 (2C), 124.2, 40.8, 35.1. HRMS (ESI) calc. for $C_{19}H_{18}NOS$ [M + H]$^+$ 308.1104; found 308.1104. HPLC-MS (gradient I) (M + H)$^+$ = 308, R_t = 6.36 min (99%).

N-(4-Morpholinophenyl)-5-phenylthiophene-2-carboxamide (**6**). The title compound was prepared by reaction of 5-phenylthiophene-2-carboxylic acid (1.5 mmol, 306 mg), 4-morpholinoaniline (1.53 mmol, 281 mg), EDCl (3 mmol, 573 mg), HOBt (3 mmol, 405 mg), Et_3N (4.5 mmol, 626 µL) and CH_2Cl_2 as solvent (6 mL) according to procedure A. Compound **6** was obtained pure after work-up. Yield: 329 mg (60%) as a white solid. Mp 289 – 291 ºC. ^{1}H RMN (300 MHz, DMSO-d_6) δ 10.09 (s, 1H), 7.98 (d, *J* = 4.0 Hz, 1H), 7.78 – 7.72 (m, 2H), 7.63 – 7.57 (m, 3H), 7.52 – 7.44 (m, 2H), 7.43 – 7.36 (m, 1H), 6.99 – 6.92 (m, 2H), 3.78 – 3.71 (m, 4H), 3.12 – 3.05 (m, 4H). ^{13}C RMN (75 MHz, DMSO-d_6) δ 159.2, 147.9, 147.6, 139.2, 133.0, 130.7, 129.7, 129.2 (2C), 128.5, 125.7 (2C), 124.3, 121.5 (2C), 115.2 (2C), 66.1 (2C), 48.8. HRMS (ESI) calc. for $C_{21}H_{21}N_2O_2S$ [M + H]$^+$ 365.1318; found 365.1304. HPLC-MS (gradient III) (M + H)$^+$ = 365, R_t = 6.36 min (99%).

N-(2-(1-Benzylpiperidin-4-yl)ethyl)-5-phenylthiophene-2-carboxamide (**7**). The title compound was prepared by reaction of 5-phenylthiophene-2-carboxylic acid (1.5 mmol, 306 mg), 2-(1-benzylpiperidin-4-yl)ethyl-1-amine (1.53 mmol, 334 mg), EDCl (3 mmol, 573 mg), Et_3N (4.5 mmol, 626 µL) and CH_2Cl_2 as solvent (6 mL) according to procedure A. Purification: CH_2Cl_2/MeOH (9:1). Yield: 108 mg (18%) as a white solid. Mp 149 – 151 ºC. ^{1}H RMN (300 MHz, DMSO-d_6) δ 8.48 (t, *J* = 5.6 Hz, 1H), 7.74 (d, *J* = 3.9 Hz, 1H), 7.72 – 7.68 (m, 2H), 7.52 (d, *J* = 3.9 Hz, 1H), 7.49 – 7.41 (m, 2H), 7.40 –7.21 (m, 6H), 3.47 (s, 2H), 3.27 (q, *J* = 7.05 Hz, 2H), 2.81 (d, *J* = 11.0 Hz, 2H), 1.95 (m, 2H), 1.69 (d, *J* = 11.4 Hz, 2H), 1.46 (q, *J* = 7.05 Hz, 2H), 1.39 – 1.26 (m, 1H), 1.18 (tt, *J* = 5.4, 11.2 Hz, 2H). ^{13}C RMN (75 MHz, DMSO-d_6) δ 160.7, 147.0, 139.2, 133.1, 129.2 (2C), 128.9, 128.8, 128.4 (2C), 128.1 (3C), 126.9, 125.6 (2C), 124.2, 62.3, 53.1 (2C), 36.8, 35.9, 32.8, 31.7 (2C). HRMS (ESI) calc. for $C_{25}H_{29}N_2OS$ [M + H]$^+$ 405.1995; found 405.1996. HPLC-MS (gradient I) (M + H)$^+$ = 405, R_t = 3.79 min (99%).

(5-Phenylthiophen-2-yl)(pyrrolidin-1-yl)methanone (**8**). The title compound was prepared by reaction of 5-phenylthiophene-2-carboxylic acid (1.5 mmol, 306 mg), pyrrolidine (1.53 mmol, 127 µL), EDCl (3 mmol, 573 mg), Et_3N (4.5 mmol, 626 µL) and CH_2Cl_2 as solvent (6 mL) according to procedure A. Purification: CH_2Cl_2/MeOH (9:1). Yield: 194 mg (50%) as a white solid. Mp 127 – 129 ºC. ^{1}H RMN (300 MHz, DMSO-d_6) δ 7.76 – 7.70 (m, 2H), 7.60 (d, *J* = 4.0 Hz, 1H), 7.54 (d, *J* = 4.0 Hz, 1H), 7.49 – 7.42 (m, 2H), 7.41 – 7.34 (m, 1H), 3.84 – 3.44 (m, 4H), 2.04 – 1.80 (m, 4H).^{13}C RMN (75 MHz, DMSO-d_6) δ 160.2, 146.8, 138.9, 132.9, 130.6, 129.2 (2C), 128.4, 125.6 (2C), 124.1, 48.2, 47.2, 26.2, 23.5. HRMS (ESI) calc. for $C_{15}H_{16}NOS$ [M + H]$^+$ 258.0947; found 258.0949. HPLC-MS (gradient I) (M + H)$^+$ = 258, R_t = 5.95 min (99%).

5-Bromo-N-phenylthiophene-2-carboxamide (**9**). The title compound was prepared by reaction of 5-bromothiophene-2-carboxylic acid (1 mmol, 207 mg), aniline (1.02 mmol, 93 µL), EDCl (1 mmol, 155 mg), Et_3N (2 mmol, 278 µL), HOBt (1 mmol, 135 mg) and CH_2Cl_2 as solvent (5 mL) according to procedure A. Compound **9** was used in the next step without any further purification. Yield: 170 mg (60%) as a white solid. Mp 127.5 – 129.5 ºC. ^{1}H NMR (300 MHz, DMSO-d_6) δ 10.28 (s, 1H), 7.87 (d, *J* = 4.1 Hz, 1H), 7.75 –

7.64 (m, 2H), 7.43 – 7.27 (m, 3H), 7.19 – 7.03 (m, 1H). ^{13}C NMR (75 MHz, DMSO) δ 159.2, 142.3, 138.8, 132.1, 130.3, 129.1, 124.4, 120.8, 118.2.

5-Bromo-N-(4-chlorophenyl)thiophene-2-carboxamide (**10**). The title compound was prepared by reaction of 5-bromothiophene-2-carboxylic acid (3 mmol, 621 mg), 4-chloroaniline (3.06 mmol, 398 mg), EDCl (3 mmol, 465 mg), Et_3N (6 mmol, 835 µL), HOBt (3 mmol, 405 mg) and CH_2Cl_2 as solvent (10 mL) according to procedure A. Compound **10** was used in the next step without any further purification. Yield: 368 mg (39%) as a white solid. ^{1}H NMR (300 MHz, DMSO-*d$_6$*) δ 10.39 (s, 1H), 7.85 (d, *J* = 4.1 Hz, 1H), 7.77 – 7.70 (m, 2H), 7.46 – 7.39 (m, 2H), 7.38 (d, *J* = 4.1 Hz, 1H). ^{13}C NMR (75 MHz, $CDCl_3$) δ 158.8, 141.4, 137.4, 131.8, 130.1, 128.7 (2C), 127.6, 121.9 (2C), 118.1.

5-Bromo-N-(3-chlorophenyl)thiophene-2-carboxamide (**11**). The title compound was prepared by reaction of 5-bromothiophene-2-carboxylic acid (1.5 mmol, 310 mg), 3-chloroaniline (1.53 mmol, 194 mg), EDCl (1.5 mmol, 233 mg), Et_3N (3 mmol, 417 µL), HOBt (1.5 mmol, 203 mg) and CH_2Cl_2 as solvent (6 mL) according to procedure A. Purification: Hex/EtOAc (9:1). Yield: 190 mg (40%) as a white solid. ^{1}H NMR (300 MHz, DMSO-*d$_6$*) δ 10.41 (s, 1H), 7.91 – 7.82 (m, 2H), 7.63 (ddd, *J* = 8.2, 2.1, 1.0 Hz, 1H), 7.43 – 7.35 (m, 2H), 7.17 (ddd, *J* = 8.0, 2.1, 1.0 Hz, 1H). ^{13}C NMR (75 MHz, DMSO-*d$_6$*) δ 158.9, 141.3, 139.9, 133.0, 131.8, 130.4, 130.3, 123.6, 119.7, 118.6, 118.3.

5-Bromo-N-phenethylthiophene-2-carboxamide (**12**). The title compound was prepared by reaction of 5-bromothiophene-2-carboxylic acid (3 mmol, 621 mg), 2-phenethylamine (3.06 mmol, 383 µL), EDCl (3 mmol, 465 mg), Et_3N (6 mmol, 835 µL), HOBt (3 mmol, 405 mg) and CH_2Cl_2 as solvent (10 mL) according to procedure A. Compound **12** was used in the next step without any further purification. Yield: 253 mg (56%) as a white solid. ^{1}H NMR (300 MHz, DMSO-*d$_6$*) δ 8.68 (t, *J* = 5.7 Hz, 1H), 7.55 (d, *J* = 4.0 Hz, 1H), 7.34 – 7.16 (m, 6H), 3.43 (dt, *J* = 8.0, 6.2 Hz, 2H), 2.81 (dd, *J* = 8.2, 6.6 Hz, 2H). ^{13}C NMR (75 MHz, DMSO-*d$_6$*) δ 159.9, 141.9, 139.3, 131.5, 128.7 (2C), 128.5, 128.4 (2C), 126.2, 116.5, 40.8, 35.1.

5-Bromo-N-(4-morpholinophenyl)thiophene-2-carboxamide (**13**). The title compound was prepared by reaction of 5-bromothiophene-2-carboxylic acid (3 mmol, 621 mg), 4-morpholinoaniline (3.06 mmol, 383 µL), EDCl (3 mmol, 561 mg), Et_3N (6 mmol, 835 µL), HOBt (3 mmol, 405 mg) and CH_2Cl_2 as solvent (10 mL) according to procedure A. Compound **13** was used in the next step without any further purification. Yield: 808 mg (73 %) as a white solid. ^{1}H NMR (300 MHz, DMSO-*d$_6$*) δ 10.22 (s, 1H), 7.89 (d, *J* = 4.1 Hz, 1H), 7.57 (d, *J* = 9.1 Hz, 2H), 7.33 (d, *J* = 4.0 Hz, 1H), 6.92 (d, *J* = 9.2 Hz, 2H), 3.80 – 3.68 (m, 4H), 3.13 – 3.01 (m, 4H). ^{13}C NMR (75 MHz, DMSO-*d$_6$*) δ 158.3, 147.7, 142.3, 131.6, 130.4, 129.5, 121.6 (2C), 117.3, 115.2 (2C), 66.1 (2C), 48.7 (2C).

N-(2-(1-Benzylpiperidin-4-yl)ethyl)-5-bromothiophene-2-carboxamide (**14**). The title compound was prepared by reaction of 5-bromothiophene-2-carboxylic acid (2 mmol, 414 mg), 2-(1-benzylpiperidin-4-yl)ethan-1-amine (2.04 mmol, 445 mg), EDCl (2 mmol, 310 mg), Et_3N (4 mmol, 557 µL), HOBt (2 mmol, 270 mg) and CH_2Cl_2 as solvent (6 mL) according to procedure A. Compound **14** was used in the next step without any further purification. Yield: 643 mg (79%) as a white solid. ^{1}H NMR (300 MHz, DMSO-*d$_6$*) δ 8.51 (t, *J* = 5.6 Hz, 1H), 7.56 (d, *J* = 4.0 Hz, 1H), 7.34 – 7.21 (m, 7H), 3.42 (s, 2H), 3.24 (q, *J* = 6.7 Hz, 2H),

2.83 – 2.71 (m, 2H), 1.94 – 1.82 (m, 2H), 1.69 – 1.60 (m, 2H), 1.47 – 1.39 (m, 2H), 1.33 – 1.22 (m, 1H), 1.22 – 1.08 (m, 2H). ^{13}C NMR (75 MHz, DMSO-d_6) δ 159.8, 142.1, 138.7, 131.4, 128.7 (2C), 128.4, 128.1 (2C), 126.7, 116.4, 62.5, 53.2 (2C), 36.8, 35.8, 32.9, 31.8 (2C).

(5-Bromothiophen-2-yl)(pyrrolidin-1-yl)methanone (**15**). The title compound was prepared by reaction of 5-bromothiophene-2-carboxylic acid (2 mmol, 414 mg), pyrrolidine (2.04 mmol, 119 μL), EDCl (2 mmol, 310 mg), Et_3N (4 mmol, 557 μL), HOBt (2 mmol, 270 mg) and CH_2Cl_2 as solvent (6 mL) according to procedure A. Compound **15** was used in the next step without any further purification. Yield: 298 mg (58%) as a yellow solid. ^{1}H NMR (300 MHz, DMSO-d_6) δ 7.10 (d, J = 3.6 Hz, 1H), 6.77 (d, J = 3.6 Hz, 1H), 3.69 (t, J = 6.7 Hz, 2H), 3.45 (t, J = 6.8 Hz, 2H), 1.98 – 1.88 (m, 2H), 1.86 – 1.77 (m, 2H). ^{13}C NMR (75 MHz, DMSO-d_6) δ 156.3, 150.3, 124.9, 118.1, 114.1, 47.8, 47.2, 26.5, 23.7.

2-Bromo-N-phenylthiazole-5-carboxamide (**65**). The title compound was prepared by reaction of 2-bromothiazole-5-carboxylic acid (4.81 mmol, 1 g), aniline (4.90 mmol, 457 mg), EDCl (4.81 mmol, 919 mg), Et_3N (9.62 mmol, 1.34 mL), HOBt (4.81 mmol, 649 mg) and CH_2Cl_2 as solvent (15 mL) according to procedure A. Purification: Hex/EtOAc (7:3). Yield: 313 mg (23%) as a white solid. ^{1}H NMR (300 MHz, DMSO-d_6) δ 10.48 (s, 1H), 8.46 (s, 1H), 7.72 – 7.65 (m, 2H), 7.41 – 7.32 (m, 2H), 7.18 – 7.09 (m, 1H). ^{13}C NMR (75 MHz, DMSO-d_6) δ 157.3, 143.8, 140.4, 139.9, 138.0, 128.8 (2C), 124.4, 120.5 (2C).

2-Bromo-N-phenethylthiazole-5-carboxamide (**66**). The title compound was prepared by reaction of 2-bromothiazole-5-carboxylic acid (4.81 mmol, 1 g), 2-phenethylamine (4.90 mmol, 614 μL), EDCl (4.81 mmol, 919 mg), Et_3N (9.62 mmol, 1.34 mL), HOBt (4.81 mmol, 649 mg) and CH_2Cl_2 as solvent (15 mL) according to procedure A. Purification: Hex/EtOAc (7:3). Yield: 619 mg (41%) as a white solid. ^{1}H NMR (300 MHz, DMSO-d_6) δ 8.87 (t, J = 5.7 Hz, 1H), 8.18 (s, 1H), 7.34 – 7.16 (m, 5H), 3.45 (ddd, J = 8.3, 7.1, 5.7 Hz, 2H), 2.82 (dd, J = 8.2, 6.6 Hz, 2H). ^{13}C NMR (75 MHz, DMSO-d_6) δ 158.5, 142.7, 139.7, 139.5, 139.1, 128.6 (2C), 128.4 (2C), 126.2, 40.8, 34.9.

N-(2-(1-Benzylpiperidin-4-yl)ethyl)-2-bromothiazole-5-carboxamide (**67**). The title compound was prepared by reaction of 2-bromothiazole-5-carboxylic acid (4.81 mmol, 1 g), 2-(1-benzylpiperidin-4-yl)ethyl-1-amine (4.90 mmol, 1.1 g), EDCl (4.81 mmol, 919 mg), Et_3N (9.62 mmol, 1.34 mL), HOBt (4.81 mmol, 649 mg) and CH_2Cl_2 as solvent (15 mL) according to procedure A. Compound **67** was used in the next step without any further purification. Yield: 668 mg (34%) as a white solid. ^{1}H NMR (300 MHz, DMSO-d_6) δ 8.72 (s, 1H), 8.19 (s, 1H), 7.34 – 7.22 (m, 5H), 3.46 (s, 2H), 3.24 (q, J = 6.6 Hz, 3H), 2.88 – 2.72 (m, 2H), 2.03 – 1.84 (m, 2H), 1.74 – 1.57 (m, 2H), 1.44 (q, J = 6.7 Hz, 2H), 1.36 – 1.24 (m, 1H), 1.24 – 1.06 (m, 3H). ^{13}C NMR (75 MHz, DMSO-d_6) δ 158.4, 142.7, 139.8, 139.4, 128.9 (2C), 128.1 (2C), 126.9, 62.3, 53.1 (2C), 36.8, 35.6, 32.7, 31.6 (2C).

5-Bromo-N-phenylfuran-2-carboxamide (**68**). The title compound was prepared by reaction of 5-bromofuran-2-carboxylic acid (5.23 mmol, 1.0 g), aniline (5.33 mmol, 480 μL), EDCl (10.46 mmol, 2.0 g), Et_3N (10.46 mmol, 1.5 mL), HOBt (5.23 mmol, 705 mg) and CH_2Cl_2 as solvent (17 mL) according to procedure A. Purification: Hex/EtOAc (9:1). Yield: 1.1 g (68%) as a white solid. ^{1}H NMR (300 MHz,

DMSO-d_6) δ 10.34 (s, 1H), 7.76 (d, *J* = 9.0 Hz, 2H), 7.46 – 7.31 (m, 2H), 6.84 (d, *J* = 3.6 Hz, 1H). ^{13}C NMR (75 MHz, DMSO-d_6) δ 155.1, 149.0, 137.3, 128.6 (2C), 127.6, 125.6, 121.9 (2C), 117.4, 114.4.

5-Bromo-N-(4-chlorophenyl)furan-2-carboxamide (**69**). The title compound was prepared by reaction of 5-bromofuran-2-carboxylic acid (5.23 mmol, 1.0 g), 4-chloroaniline (5.33 mmol, 677 mg), EDCl (10.46 mmol, 2.0 g), Et_3N (10.46 mmol, 1.5 mL), HOBt (10.46 mmol, 1.41 g) and CH_2Cl_2 as solvent (17 mL) according to procedure A. Purification: CH_2Cl_2/MeOH (95:5). Yield: 1.1 g (80%) as a white solid. ^{1}H NMR (300 MHz, DMSO-d_6) δ 10.20 (s, 1H), 7.74 – 7.69 (m, 2H), 7.39 – 7.30 (m, 3H), 7.11 (m, 1H), 6.83 (d, *J* = 3.6 Hz, 1H). ^{13}C NMR (75 MHz, DMSO-d_6) δ 155.1, 149.3, 138.3, 128.7 (2C), 125.3, 123.9, 120.5 (2C), 117.1, 114.3.

5-Bromo-N-(3-chlorophenyl)furan-2-carboxamide (**70**). The title compound was prepared by reaction of 5-bromofuran-2-carboxylic acid (7.85 mmol, 1.5 g), 3-chloroaniline (8.01 mmol, 1.02 g), EDCl (7.85 mmol, 1.22 g), Et_3N (15.7 mmol, 2.2 mL), HOBt (7.85 mmol, 1.06 mg) and CH_2Cl_2 as solvent (16 mL) according to procedure A for 5 days. Purification: Hex/EtOAc (9:1). Yield: 760 mg (32%) as an orange solid. ^{1}H NMR (300 MHz, DMSO-d_6) δ 10.36 (s, 1H), 7.90 (t, *J* = 2.0 Hz, 1H), 7.67 (ddd, *J* = 8.2, 2.0, 1.0 Hz, 1H), 7.44 – 7.32 (m, 2H), 7.16 (ddd, *J* = 8.0, 2.1, 1.0 Hz, 1H), 6.85 (d, *J* = 3.6 Hz, 1H). ^{13}C NMR (75 MHz, DMSO-d_6) δ 155.2, 148.9, 139.8, 132.9, 130.4, 125.7, 123.6, 119.8, 118.7, 117.6, 114.4.

N-(2-(1-Benzylpiperidin-4-yl)ethyl)-5-bromofuran-2-carboxamide (**71**). The title compound was prepared by reaction of 5-bromofuran-2-carboxylic acid (7.85 mmol, 1.5 g), 2-(1-benzylpiperidin-4-yl)ethyl-1-amine (8.01 mmol, 1.75 g), EDCl (7.85 mmol, 1.22 g), Et_3N (15.7 mmol, 2.2 mL), HOBt (7.85 mmol, 1.06 g) and CH_2Cl_2 as solvent (22 mL) according to procedure A for 4 days. Compound **71** was obtained pure and used in the next step without any further purification. Yield: 2.2 g (73%) as an orange solid. ^{1}H NMR (300 MHz, DMSO-d_6) δ 8.39 (t, *J* = 5.7 Hz, 1H), 7.34 – 7.20 (m, 5H), 7.09 (d, *J* = 3.5 Hz, 1H), 6.73 (d, *J* = 3.5 Hz, 1H), 3.42 (s, 2H), 3.27 – 3.17 (m, 2H), 2.82 – 2.72 (m, 2H), 1.94 – 1.81 (m, 2H), 1.70 – 1.60 (m, 2H), 1.47 – 1.37 (m, 2H), 1.30 – 1.19 (m, 1H), 1.18 – 1.07 (m, 2H). ^{13}C NMR (75 MHz, DMSO-d_6) δ 156.5, 149.9, 138.5, 128.8 (2C), 128.1 (2C), 126.8, 124.1, 115.5, 113.9, 62.5, 53.2 (2C), 36.2, 35.9, 32.8, 31.8 (2C).

5-Bromo-N-phenethylfuran-2-carboxamide (**72**). The title compound was prepared by reaction of 5-bromofuran-2-carboxylic acid (5.23 mmol, 1.0 g), 2-phenethylamine (5.33 mmol, 646 mg), EDCl (5.24 mmol, 1.0 g), Et_3N (10.46 mmol, 1.5 mL), HOBt (5.24 mmol, 707 mg) and CH_2Cl_2 as solvent (15 mL) according to procedure A. Purification: Hex/EtOAc (9:1). Yield: 964 mg (63%) as a yellow oil. ^{1}H NMR (300 MHz, DMSO-d_6) δ 8.51 (t, *J* = 5.7 Hz, 1H), 7.35 – 7.14 (m, 5H), 7.10 (d, *J* = 3.5 Hz, 1H), 6.73 (d, *J* = 3.5 Hz, 1H), 3.48 – 3.37 (m, 2H), 2.86 – 2.76 (m, 2H). ^{13}C NMR (75 MHz, DMSO-d_6) δ 156.6, 149.8, 139.3, 128.6 (2C), 128.3 (2C), 126.1, 124.2, 115.6, 113.9, 35.0.

Chemical procedures for the synthesis of hidroxyphenyl-heterocycle derivatives 16-31 and 83-89 following general procedure B

5-(2-Hydroxyphenyl)-N-phenylthiophene-2-carboxamide (**16**). The title compound was prepared by reaction of 5-bromo-*N*-phenylthiophene-2-carboxamide (**9**) (1.48 mmol, 417 mg), Na_2CO_3 (3.26 mmol, 345 mg), $Pd(PPh_3)_4$ (0. 074 mmol, 87 mg), 2-(4,4,5,5-tetramethyl-1,3,2-dioxaborolan-2-yl)phenol (1.77 mmol, 372 μL) and a solution of toluene (3.1 mL), H_2O (2.33 mL) and EtOH (1.57 mL) as solvent according to procedure B. Purification: Hex/EtOAc (6:4). Yield: 118 mg (27%) as a yellow solid. Mp 251 – 253 ºC. ^1H RMN (300 MHz, DMSO-d_6) δ 10.53 (s, 1H), 10.16 (s, 1H), 7.99 (d, J = 4.1 Hz, 1H), 7.79 – 7.72 (m, 3H), 7.70 (d, J = 4.1 Hz, 1H), 7.40 – 7.33 (m, 2H), 7.21 (ddd, J = 8.7, 7.2, 1.6 Hz, 1H), 7.14 – 7.06 (m, 1H), 7.00 (dd, J = 8.2, 1.2 Hz, 1H), 6.91 (ddd, J = 8.3, 7.3, 1.2 Hz, 1H). ^{13}C RMN (75 MHz, DMSO-d_6) δ 160.3, 153.8, 144.6, 138.9, 138.2, 129.4, 128.8, 128.7 (2C), 127.6, 124.8, 123.6, 120.3 (2C), 119.9, 119.6, 116.4. HRMS (ESI) calc. for $C_{17}H_{14}NO_2S$ $[M + H]^+$ 296.0740; found 296.0733. HPLC-MS (gradient I) $(M + H)^+$ = 296, R_t = 3.38 min (99%).

N-(4-Chlorophenyl)-5-(2-hydroxyphenyl)thiophene-2-carboxamide (**17**). The title compound was prepared by reaction of 5-bromo-*N*-(4-chlorophenyl)thiophene-2-carboxamide (**10**) (1.16 mmol, 368 mg), Na_2CO_3 (2.55 mmol, 270 mg), $Pd(PPh_3)_4$ (0. 058 mmol, 68 mg), 2-(4,4,5,5-tetramethyl-1,3,2-dioxaborolan-2-yl)phenol (1.4 mmol, 293 μL) and a solution of toluene (2 mL), H_2O (1.5 mL) and EtOH (1 mL) as solvent according to procedure B. Purification: Hex/EtOAc (9:1). Yield: 100 mg (26%) as a yellow solid. Mp 272 – 274 ºC. ^1H RMN (300 MHz, DMSO-d_6) δ 10.56 (s, 1H), 10.29 (s, 1H), 7.98 (d, J = 4.1 Hz, 1H), 7.82 – 7.73 (m, 3H), 7.70 (d, J = 4.1 Hz, 1H), 7.46 – 7.37 (m, 2H), 7.20 (ddd, J = 8.3, 7.3, 1.6 Hz, 1H), 6.99 (dd, J = 8.2, 1.2 Hz, 1H), 6.90 (ddd, J = 8.3, 7.2, 1.2 Hz, 1H). ^{13}C RMN (75 MHz, DMSO-d_6) δ 160.3, 153.8, 144.8, 137.9, 137.7, 129.4, 129.0, 128.5 (2C), 127.5, 127.1, 124.8, 121.7 (2C), 119.8, 119.6, 116.4. HRMS (ESI) calc. for $C_{17}H_{13}ClNO_2S$ $[M + H]^+$ 330.0350; found 330.0335. HPLC-MS (gradient III) $(M + H)^+$ = 330, R_t = 5.63 min (98%).

N-(3-Chlorophenyl)-5-(2-hydroxyphenyl)thiophene-2-carboxamide (**18**). The title compound was prepared by reaction of 5-bromo-*N*-(3-chlorophenyl)thiophene-2-carboxamide (**11**) (0.84 mmol, 265 mg), Na_2CO_3 (1.84 mmol, 195 mg), $Pd(PPh_3)_4$ (0. 042 mmol, 49 mg), 2-(4,4,5,5-tetramethyl-1,3,2-dioxaborolan-2-yl)phenol (1.00 mmol, 211 μL) and a solution of toluene (2 mL), H_2O (2.5 mL) and EtOH (1 mL) as solvent according to procedure B. Purification: Hex/EtOAc (9:1). Yield: 120 mg (44%) as a white solid. Mp 246 – 248 ºC. ^1H RMN (300 MHz, DMSO-d_6) δ 10.56 (s, 1H), 10.31 (s, 1H), 8.00 (d, J = 4.1 Hz, 1H), 7.94 (t, J = 2.0 Hz, 1H,), 7.77 (dd, J = 7.9, 1.7 Hz, 1H), 7.73 – 7.67 (m, 2H), 7.39 (t, J = 8.1 Hz, 1H), 7.24 – 7.13 (m, 2H), 7.01 (dd, J = 8.2, 1.2 Hz, 1H), 6.91 (ddd, J = 7.8, 7.2, 1.2 Hz, 1H). ^{13}C RMN (75 MHz, DMSO-d_6) δ 160.5, 153.8, 145.0, 140.4, 137.5, 132.9, 130.4 (2C), 129.5, 129.2, 127.6, 124.8, 123.2, 119.8, 119.6, 118.5, 116.4. HRMS (ESI) calc. for $C_{17}H_{13}ClNO_2S$ $[M + H]^+$ 330.0350; found 330.0348. HPLC-MS (gradient III) $(M + H)^+$ = 330, R_t = 5.65 min (96%).

5-(2-Hydroxyphenyl)-N-phenethylthiophene-2-carboxamide (**19**). The title compound was prepared by reaction of 5-bromo-*N*-phenethylthiophene-2-carboxamide (**12**) (2.23 mmol, 692 mg), Na_2CO_3 (4.91 mmol,

520 mg), $Pd(PPh_3)_4$ (0. 114 mmol, 131 mg), 2-(4,4,5,5-tetramethyl-1,3,2-dioxaborolan-2-yl)phenol (2.68 mmol, 561 µL) and a solution of toluene (4 mL), H_2O (3 mL) and EtOH (2 mL) as solvent according to procedure B. Compound **19** was obtained pure after work-up. Yield: 263 mg (45%) as a white solid. Mp 200 – 202 ºC. ^{1}H RMN (300 MHz, DMSO-*d₆*) δ 10.42 (s, 1H), 8.53 (t, *J* = 5.7 Hz, 1H), 7.72 – 7.64 (m, 2H), 7.58 (d, *J* = 4.0 Hz, 1H), 7.34 – 7.13 (m, 6H), 6.97 (dd, *J* = 8.2, 1.2 Hz, 1H), 6.87 (td, *J* = 7.5, 1.3 Hz, 1H), 3.46 (dt, *J* = 8.0, 6.1 Hz, 2H), 2.84 (dd, *J* = 8.3, 6.6 Hz, 2H). ^{13}C RMN (75 MHz, DMSO-d_6) δ 161.4, 153.7, 143.4, 139.5, 138.3, 129.1, 128.6 (2C), 128.3 (2C), 127.6, 126.1, 124.7, 120.0, 119.5, 116.3, 40.7, 35.2. HRMS (ESI): calc. for $C_{19}H_{18}NO_2S$ $[M + H]^+$ 324.1053; found 324.1043. HPLC-MS (gradient III) $(M + H)^+$ = 324, R_t = 5.08 min (99%).

5-(2-Hydroxyphenyl)-N-(4-morpholinophenyl)thiophene-2-carboxamide (**20**). The title compound was prepared by reaction of 5-bromo-*N*-(4-morpholinophenyl)thiophene-2-carboxamide (**13**) (2.2 mmol, 809 mg), Na_2CO_3 (4.84 mmol, 513 mg), $Pd(PPh_3)_4$ (0. 11 mmol, 130 mg), 2-(4,4,5,5-tetramethyl-1,3,2-dioxaborolan-2-yl)phenol (2.64 mmol, 553 µL) and a solution of toluene (3.1 mL), H_2O (2.3 mL) and EtOH (1.55 mL) as solvent according to procedure B. Compound **20** was obtained pure after washing with ethyl acetate. Yield: 774 mg (92%) as a yellow solid. Mp 264 – 266 ºC. ^{1}H RMN (300 MHz, DMSO-d_6) δ 9.99 (s, 1H), 7.92 (d, *J* = 4.1 Hz, 1H), 7.72 (dd, *J* = 8.0, 1.6 Hz, 1H), 7.65 (d, *J* = 4.1 Hz, 1H), 7.63 – 7.56 (m, 2H), 7.13 (td, *J* = 7.6, 7.1, 1.6 Hz, 1H), 7.01 (dd, *J* = 8.3, 1.2 Hz, 1H), 6.97 – 6.89 (m, 2H), 6.85 – 6.75 (m, 1H), 3.80 – 3.67 (m, 4H), 3.13 – 3.00 (m, 4H). ^{13}C RMN (75 MHz, DMSO-d_6) δ 160.0, 155.7, 147.3, 144.8, 137.9, 131.1, 129.1, 128.1, 127.1, 123.9, 121.4 (2C), 119.9, 118.1, 116.8, 115.3 (2C), 66.1 (2C), 48.9 (2C). HRMS (ESI) calc. for $C_{21}H_{21}N_2O_3S$ $[M + H]^+$ 381.1267; found 381.1258. HPLC-MS (gradient I) $(M + H)^+$ = 381, R_t = 5.22 min (98%).

N-(2-(1-Benzylpiperidin-4-yl)ethyl)-5-(2-hydroxyphenyl)thiophene-2-carboxamide (**21**). The title compound was prepared by reaction of *N*-(2-(1-benzylpiperidin-4-yl)ethyl)-5-bromothiophene-2-carboxamide (**14**) (1.47 mmol, 601 mg), Na_2CO_3 (3.23 mmol, 343 mg), $Pd(PPh_3)_4$ (0. 074 mmol, 87 mg), 2-(4,4,5,5-tetramethyl-1,3,2-dioxaborolan-2-yl)phenol (1.77 mmol, 371 µL) and a solution of toluene (4 mL), H_2O (3 mL) and EtOH (2 mL) as solvent according to procedure B. Purification: CH_2Cl_2/MeOH (95:5). Yield: 116 mg (20%) as a white solid. Mp 185 – 187 ºC. ^{1}H RMN (300 MHz, DMSO-d_6) δ 10.42 (s, 1H), 8.37 (t, *J* = 5.6 Hz, 1H), 7.73 – 7.64 (m, 2H), 7.58 (d, *J* = 4.0 Hz, 1H), 7.35 – 7.20 (m, 5H), 7.16 (td, *J* = 7.7, 7.2, 1.6 Hz, 1H), 6.97 (dd, *J* = 8.2, 1.2 Hz, 1H), 6.87 (td, *J* = 7.5, 1.2 Hz, 1H), 3.45 (s, 2H), 3.29 – 3.21 (m, 2H), 2.89 – 2.69 (m, 2H,) 2.03 – 1.81 (m, 2H), 1.73 – 1.61 (m, 2H), 1.52 – 1.38 (m, 2H), 1.37 – 1.24 (m, 1H), 1.23 – 1.08 (m, 2H). ^{13}C RMN (75 MHz, DMSO-d_6) δ 161.3, 153.7, 143.2, 138.5, 129.1, 128.8, 128.1 (2C), 127.6, 127.4, 126.9, 124.7, 120.0, 119.5, 116.3, 62.4, 53.2 (2C), 36.7, 36.0, 32.9, 31.8 (2C). HRMS (ESI) calc. for $C_{25}H_{29}N_2O_2S$ $[M + H]^+$ 421.1944; found 421.1946. HPLC-MS (gradient III) $(M + H)^+$ = 421, R_t = 2.49 min (99%).

(5-(2-Hydroxyphenyl)thiophen-2-yl))(pyrrolidin-1-yl)methanone (**22**). The title compound was prepared by reaction of (5-bromothiophen-2-yl)(pyrrolidin-1-yl)methanone (**15**) (1.74 mmol, 452 mg), Na_2CO_3 (3.83 mmol, 406 mg), $Pd(PPh_3)_4$ (0. 088 mmol, 103 mg), 2-(4,4,5,5-tetramethyl-1,3,2-dioxaborolan-2-yl)phenol

(2.1 mmol, 437 μL) and a solution of toluene (2 mL), H_2O (1.5 mL) and EtOH (1 mL) as solvent according to procedure B. Compound **22** was obtained pure after washing with hexane. Yield: 358 mg (76%) as a white solid. Mp 235 – 237 ºC. ^{1}H RMN (300 MHz, DMSO-*d₆*) δ 10.42 (s, 1H), 7.71 (dd, *J* = 7.8, 1.6 Hz, 1H), 7.60 (d, *J* = 4.1 Hz, 1H), 7.55 (d, *J* = 4.1 Hz, 1H), 7.18 (ddd, *J* = 8.7, 7.2, 1.6 Hz, 1H), 6.98 (dd, *J* = 8.2, 1.2 Hz, 1H), 6.88 (td, *J* = 7.6, 1.2 Hz, 1H), 3.88 – 3.41 (m, 4H), 2.02 – 1.74 (m, 4H). ^{13}C RMN (75 MHz, DMSO-d_6) δ 160.9, 153.8, 143.1, 138.2, 129.1, 127.6, 124.7, 119.9, 119.5, 116.3, 48.3, 47.1, 26.3, 23.5. HRMS (ESI) calc. for $C_{15}H_{16}NO_2S$ $[M + H]^+$ 274.0896; found 274.0894. HPLC-MS (gradient III) $(M + H)^+$ = 274, R_t = 4.30 min (99%).

5-(3-Hydroxyphenyl)-N-phenylthiophene-2-carboxamide (**23**). The title compound was prepared by reaction of 5-bromo-*N*-phenylthiophene-2-carboxamide (**9**) (1.42 mmol, 404 mg), Na_2CO_3 (2.84 mmol, 301 mg), $Pd(PPh_3)_4$ (0. 070 mmol, 80 mg), 3-(4,4,5,5-tetramethyl-1,3,2-dioxaborolan-2-yl)phenol (1.56 mmol, 344 mg) and a solution of toluene (3.1 mL), H_2O (2.33 mL) and EtOH (1.57 mL) as solvent according to procedure B. Purification: Hex/EtOAc (9:1). Yield: 180 mg (43%) as a yellow solid. ^{1}H NMR (300 MHz, DMSO-d_6) δ 10.24 (s, 1H), 9.71 (s, 1H), 8.02 (d, *J* = 4.0 Hz, 1H), 7.82 – 7.67 (m, 2H), 7.55 (d, *J* = 3.9 Hz, 1H), 7.37 (dd, *J* = 8.5, 7.3 Hz, 2H), 7.27 (t, *J* = 7.8 Hz, 1H), 7.18 (ddd, *J* = 7.7, 1.8, 1.1 Hz, 1H), 7.15 – 7.08 (m, 2H), 6.81 (ddd, *J* = 8.0, 2.4, 1.1 Hz, 1H). ^{13}C NMR (75 MHz, DMSO-d_6) δ 160.1, 158.4, 149.0, 139.2, 139.1, 134.6, 130.8, 130.7, 129.1, 124.7, 124.2, 120.8, 117.0, 116.3, 112.8.

N-(4-Chlorophenyl)-5-(3-hydroxyphenyl)thiophene-2-carboxamide (**24**). The title compound was prepared by reaction of 5-bromo-*N*-(4-chlorophenyl)thiophene-2-carboxamide (**10**) (1.28 mmol, 404 mg), Na_2CO_3 (2.82 mmol, 298 mg), $Pd(PPh_3)_4$ (0. 065 mmol, 75 mg), 3-(4,4,5,5-tetramethyl-1,3,2-dioxaborolan-2-yl)phenol (1.54 mmol, 338 mg) and a solution of toluene (3.1 mL), H_2O (2.33 mL) and EtOH (1.57 mL) as solvent according to procedure B. Purification: Hex/EtOAc (9:1). Yield: 180 mg (43%) as a yellow solid. Mp decomposition. ^{1}H RMN (300 MHz, DMSO-d_6) δ 10.35 (s, 1H), 9.71 (s, 1H), 8.00 (d, *J* = 4.0 Hz, 1H), 7.83 – 7.73 (m, 2H), 7.55 (d, *J* = 3.9 Hz, 1H), 7.48 – 7.38 (m, 2H), 7.26 (t, *J* = 7.8 Hz), 7.17 (dt, *J* = 7.7, 1.3 Hz, 1H), 7.10 (t, *J* = 2.1 Hz, 1H), 6.80 (ddd, *J* = 8.0, 2.4, 1.1 Hz, 1H). ^{13}C RMN (75 MHz, DMSO-d_6) δ 159.7, 157.9, 148.8, 138.2, 137.7, 134.1, 130.4, 128.6 (2C), 127.4, 124.3, 121.8 (2C), 116.6, 115.9, 112.4. HRMS (ESI) calc. for $C_{17}H_{13}NO_2SCl$ $[M + H]^+$ 330.0350; found 330.0355. HPLC-MS (gradient II) $(M + H)^+$ = 330, R_t = 3.56 min (99%).

N-(3-Chlorophenyl)-5-(3-hydroxyphenyl)thiophene-2-carboxamide (**25**). The title compound was prepared by reaction of 5-bromo-*N*-(3-chlorophenyl)thiophene-2-carboxamide (**11**) (0.32 mmol, 101 mg), Na_2CO_3 (0.71 mmol, 75 mg), $Pd(PPh_3)_4$ (0. 016 mmol, 19 mg), 3-(4,4,5,5-tetramethyl-1,3,2-dioxaborolan-2-yl)phenol (0.98 mmol, 85 mg) and a solution of toluene (2 mL), H_2O (1.5 mL) and EtOH (1 mL) as solvent according to procedure B. Purification: Hex/EtOAc (1:1). Yield: 68 mg (64%) as a white solid. Mp 190 – 192 ºC. ^{1}H RMN (300 MHz, DMSO-d_6) δ 10.37 (s, 1H), 9.70 (s, 1H), 8.01 (d, *J* = 3.9 Hz, 1H), 7.92 (t, *J* = 2.0 Hz, 1H), 7.72 – 7.63 (m, 1H), 7.55 (d, *J* = 4.0 Hz, 1H), 7.39 (t, *J* = 8.1 Hz, 1H), 7.26 (t, 1H), 7.21 – 7.14 (m, 2H), 7.13 – 7.08 (m, 1H), 6.83 – 6.77 (m, 1H). ^{13}C RMN (75 MHz, DMSO-d_6) δ 159.9, 157.9, 149.0, 140.2, 137.9, 134.0, 132.9, 130.6, 130.4, 130.4, 124.3, 123.4, 119.6, 118.6, 116.6, 115.9, 112.4. HRMS (ESI) calc. for

$C_{17}H_{13}NO_2SCl$ [M + H]$^+$ 330.0350; found 330.0347. HPLC-MS (gradient II) (M + H)$^+$ = 330, R_t = 3.61 min (99%).

5-(3-Hydroxyphenyl)-N-phenethylthiophene-2-carboxamide (**26**). The title compound was prepared by reaction of 5-bromo-*N*-phenethylthiophene-2-carboxamide (**12**) (1.13 mmol, 350 mg), Na_2CO_3 (2.49 mmol, 264 mg), $Pd(PPh_3)_4$ (0. 058 mmol, 67 mg), 3-(4,4,5,5-tetramethyl-1,3,2-dioxaborolan-2-yl)phenol (1.36 mmol, 298 mg) and a solution of toluene (2 mL), H_2O (1.5 mL) and EtOH (1 mL) as solvent according to procedure B. Purification: Hex/EtOAc (1:1). Yield: 70 mg (19%) as a white solid. Mp 183 – 185 ºC. ^{1}H RMN (300 MHz, DMSO-d_6) δ 9.67 (s, 1H), 8.62 (t, *J* = 5.6 Hz, 1H), 7.69 (d, *J* = 3.9 Hz, 1H), 7.44 (d, *J* = 3.9 Hz, 1H), 7.34 – 7.17 (m, 6H), 7.12 (dt, *J* = 7.7, 1.3 Hz, 1H), 7.05 (t, *J* = 2.0 Hz, 1H), 6.77 (ddd, *J* = 8.0, 2.4, 1.0 Hz, 1H), 3.46 (dt, *J* = 8.1, 6.2 Hz, 2H), 2.84 (dd, *J* = 8.4, 6.5 Hz, 2H). ^{13}C RMN (75 MHz, DMSO-d_6) δ 160.9, 157.9, 147.4, 139.4, 138.8, 134.3, 130.3, 128.9, 128.7 (2C), 128.4 (2C), 126.1, 124.1, 116.5, 115.6, 112.3, 40.8, 35.2. HRMS (ESI) calc. for $C_{19}H_{18}NO_2S$ [M + H]$^+$ 324.1053; found 324.1049. HPLC-MS (gradient II) (M + H)$^+$ = 324, R_t = 3.24 min (99%).

5-(4-Hydroxyphenyl)-N-phenylthiophene-2-carboxamide (**27**). The title compound was prepared by reaction of 5-bromo-*N*-phenylthiophene-2-carboxamide (**9**) (2.0 mmol, 574 mg), Na_2CO_3 (4.0 mmol, 424 mg), $Pd(PPh_3)_4$ (0.05 mmol, 58 mg), 4-(4,4,5,5-tetramethyl-1,3,2-dioxaborolan-2-yl)phenol (2.2 mmol, 484 mg) and a solution of toluene (4 mL), H_2O (3 mL) and EtOH (2 mL) as solvent according to procedure B. Purification: Hex/EtOAc (9:1). Yield: 336 mg (57%) as a white solid. ^{1}H NMR (300 MHz, DMSO-d_6) δ 10.17 (s, 1H), 9.84 (s, 1H), 7.98 (d, *J* = 4.0 Hz, 1H), 7.82 – 7.67 (m, 2H), 7.64 – 7.50 (m, 2H), 7.42 (d, *J* = 3.9 Hz, 1H), 7.40 – 7.32 (m, 2H), 7.17 – 7.02 (m, 1H), 6.96 – 6.75 (m, 2H). ^{13}C NMR (75 MHz, DMSO-d_6) δ 160.3, 158.6, 149.7, 139.3, 137.5, 130.7, 129.1, 127.7, 124.6, 124.1, 122.9, 120.7, 116.4.

N-(4-Chlorophenyl)-5-(4-hydroxyphenyl)thiophene-2-carboxamide (**28**). The title compound was prepared by reaction of 5-bromo-*N*-(4-chlorophenyl)thiophene-2-carboxamide (**10**) (1.58 mmol, 500 mg), Na_2CO_3 (3.48 mmol, 368 mg), $Pd(PPh_3)_4$ (0. 081 mmol, 93 mg), 4-(4,4,5,5-tetramethyl-1,3,2-dioxaborolan-2-yl)phenol (1.89 mmol, 417 mg) and a solution of toluene (4 mL), H_2O (3 mL) and EtOH (2 mL) as solvent according to procedure B. Purification: Hex/EtOAc (9:1). Yield: 208 mg (40%) as a yellow solid. Mp 291 – 293 ºC. ^{1}H RMN (300 MHz, DMSO-d_6) δ 10.28 (s, 1H), 9.83 (s, 1H), 7.96 (d, *J* = 4.0 Hz, 1H), 7.81 – 7.72 (m, 2H), 7.62 – 7.52 (m, 2H), 7.46 – 7.37 (m, 3H), 6.88 – 6.78 (m, 2H). ^{13}C RMN (75 MHz, DMSO-d_6) δ 159.9, 158.2, 149.5, 137.8, 136.6, 130.5, 128.6 (2C), 127.2 (2C), 127.2, 124.1, 122.5, 121.7 (2C), 115.9 (2C). HRMS (ESI) calc. for $C_{17}H_{13}NO_2SCl$ [M + H]$^+$ 330.0350; found 330.0351. HPLC-MS (gradient II) (M + H)$^+$ = 330, R_t = 3.55 min (99%).

N-(3-Chlorophenyl)-5-(4-hydroxyphenyl)thiophene-2-carboxamide (**29**). The title compound was prepared by reaction of 5-bromo-*N*-(3-chlorophenyl)thiophene-2-carboxamide (**11**) (1.27 mmol, 400 mg), Na_2CO_3 (2.78 mmol, 295 mg), $Pd(PPh_3)_4$ (0. 065 mmol, 75 mg), 4-(4,4,5,5-tetramethyl-1,3,2-dioxaborolan-2-yl)phenol (1.52 mmol, 334 mg) and a solution of toluene (3.1 mL), H_2O (2.33 mL) and EtOH (1.57 mL) as solvent according to procedure B. Purification: Hex/EtOAc (9:1). Yield: 73 mg (18%) as a yellow solid. Mp decomposition. ^{1}H RMN (300 MHz, DMSO-d_6) δ 10.31 (s, 1H), 9.84 (s, 1H), 7.97 (d, *J* = 4.0 Hz), 7.91 (t, *J*

= 2.0 Hz), 7.67 (ddd, *J* = 8.3, 2.1, 1.0 Hz, 1H), 7.61 – 7.54 (m, 2H), 7.43 (d, *J* = 4.0 Hz, 1H), 7.38 (t, *J* = 8.1 Hz, 1H), 7.15 (ddd, *J* = 8.0, 2.1, 0.9 Hz, 1H), 6.88 – 6.81 (m, 2H). ^{13}C RMN (75 MHz, DMSO-d_6) δ 159.9, 158.2, 149.8, 140.3, 136.4, 132.9, 130.7, 130.4, 127.3 (2C), 126.9, 124.0, 123.3, 122.6, 119.6, 118.5 (2C). HRMS (ESI) calc. for $C_{17}H_{13}NO_2SCl$ $[M + H]^+$ 330.0350; found 330.0346. HPLC-MS (gradient II) $(M + H)^+$ = 330, R_t = 3.50 min (99%).

5-(4-Hydroxyphenyl)-N-phenethylthiophene-2-carboxamide (**30**). The title compound was prepared by reaction of 5-bromo-*N*-phenethylthiophene-2-carboxamide (**12**) (0.68 mmol, 211 mg), Na_2CO_3 (1.59 mmol, 158 mg), $Pd(PPh_3)_4$ (0. 035 mmol, 40 mg), 4-(4,4,5,5-tetramethyl-1,3,2-dioxaborolan-2-yl)phenol (0.82 mmol, 179 mg) and a solution of toluene (2 mL), H_2O (1.5 mL) and EtOH (1 mL) as solvent according to procedure B. Purification: Hex/EtOAc (7:3). Yield: 75 mg (34%) as a yellow solid. Mp 164 – 166 ºC. ^{1}H RMN (300 MHz, DMSO-d_6) δ 9.77 (s, 1H), 8.54 (t, *J* = 5.6 Hz, 1H), 7.65 (d, *J* = 3.9 Hz, 1H), 7.56 – 7.47 (m, 2H), 7.35 – 7.17 (m, 6H), 6.88 – 6.78 (m, 2H), 3.45 (dt, *J* = 8.0, 6.0 Hz), 2.83 (dd, *J* = 8.4, 6.5 Hz, 2H). ^{13}C RMN (75 MHz, DMSO-d_6) δ 161.0, 157.9, 148.0, 139.4, 137.3, 128.9, 128.7 (2C), 128.4 (2C), 127.1 (2C), 126.1, 124.3, 122.3, 115.9 (2C), 40.8, 35.2. HRMS (ESI) calc. for $C_{19}H_{18}NO_2S$ $[M + H]^+$ 324.1053; found 324.1042. HPLC-MS (gradient II) $(M + H)^+$ = 324, R_t = 3.26 min (99%).

N-(2-(1-Benzylpiperidin-4-yl)ethyl)-5-(4-hydroxyphenyl)thiophene-2-carboxamide (**31**). The title compound was prepared by reaction of *N*-(2-(1-benzylpiperidin-4-yl)ethyl)-5-bromothiophene-2-carboxamide (**14**) (2.21 mmol, 900 mg), Na_2CO_3 (4.86 mmol, 515 mg), $Pd(PPh_3)_4$ (0. 11 mmol, 127 mg), 4-(4,4,5,5-tetramethyl-1,3,2-dioxaborolan-2-yl)phenol (2.65 mmol, 583 mg) and a solution of toluene (4 mL), H_2O (3 mL) and EtOH (2 mL) as solvent according to procedure B. Purification: CH_2Cl_2/MeOH (95:5). Yield: 550 mg (60%) as a white solid. Mp 164 – 166 ºC. ^{1}H RMN (300 MHz, DMSO-d_6) δ 9.77 (s, 1H), 8.37 (t, *J* = 5.4 Hz, 1H), 7.66 (d, *J* = 3.9 Hz, 1H), 7.50 (d, *J* = 8.6 Hz, 2H), 7.35 – 7.20 (m, 6H), 6.82 (d, *J* = 8.6 Hz, 2H), 3.44 (s, 2H), 3.25 (m, 2H), 2.78 (m, 2H), 2.03 – 1.82 (m, 2H), 1.72 – 1.60 (m, 2H), 1.51 – 1.38 (m, 2H), 1.36 – 1.24 (m, 1H), 1.23 – 1.08 (m, 2H). ^{13}C RMN (75 MHz, DMSO-d_6) δ 160.9, 157.9, 147.9, 137.4, 128.8, 128.1 (5C), 127.1 (2C), 126.9, 124.3, 122.3, 115.9 (2C), 62.4, 53.2 (2C), 36.8, 35.9, 32.9, 31.8 (2C). HRMS (ESI) calc. for $C_{25}H_{29}N_2O_2S$ $[M + H]^+$ 421.1944; found 421.1948. HPLC-MS (gradient II) $(M + H)^+$ = 421, R_t = 2.28 min (98%).

2-(2-Hydroxyphenyl)-N-phenethylthiazole-5-carboxamide (**83**). The title compound was prepared by reaction of 2-bromo-*N*-phenethylthiazole-5-carboxamide (**66**) (1.90 mmol, 593 mg), Na_2CO_3 (4.19 mmol, 444 mg), $Pd(PPh_3)_4$ (0.097 mmol, 112 mg), 2-(4,4,5,5-tetramethyl-1,3,2-dioxaborolan-2-yl)phenol (2.29 mmol, 479 µL) and a solution of toluene (2.66 mL), H_2O (2 mL) and EtOH (1.33 mL) as solvent according to procedure B. Purification: Hex/EtOAc (8:2). Yield: 266 mg (43%) as a yellow solid. Mp 175.0 – 177.0 ºC. ^{1}H NMR (300 MHz, DMSO-d_6) δ 11.32 (s, 1H), 8.76 (t, *J* = 5.6 Hz, 1H), 8.40 (s, 1H), 8.16 (dd, *J* = 7.9, 1.7 Hz, 1H), 7.39 – 7.17 (m, 6H), 7.05 (d, *J* = 7.4 Hz, 1H), 7.01 – 6.93 (m, 1H), 3.55 – 3.44 (m, 2H), 2.85 (t, *J* = 7.4 Hz). ^{13}C NMR (75 MHz, DMSO- d_6) δ 165.6, 160.3, 155.3, 142.1, 139.4, 134.5, 131.7, 128.7 (2C), 128.4 (2C), 127.5, 126.2, 119.6, 119.1, 116.5, 40.8, 35.1. HRMS (ESI) m/z: $[M + H]^+$ calc. for $C_{18}H_{17}N_2O_2S$ 325.1005; found 325.1005. HPLC-MS (gradient II) $(M + H)^+$ = 325, R_t = 3.72 min (99%).

2-(3-Hydroxyphenyl)-N-phenylthiazole-5-carboxamide (**84**). The title compound was prepared by reaction of 2-bromo-*N*-phenylthiazole-5-carboxamide (**65**) (2.21 mmol, 622 mg), Na_2CO_3 (2.66 mmol, 282 mg), $Pd(PPh_3)_4$ (0.111 mmol, 128 mg), 3-(4,4,5,5-tetramethyl-1,3,2-dioxaborolan-2-yl)phenol (2.66 mmol, 584 mg) and a solution of toluene (4 mL), H_2O (3 mL) and EtOH (2 mL) as solvent according to procedure B. Purification: Hex/EtOAc (7:3). Yield: 309 mg (47%) as a brown solid. ^{1}H NMR (300 MHz, DMSO-d_6) δ 10.45 (s, 1H), 9.87 (s, 1H), 8.66 (s, 1H), 7.78 – 7.63 (m, 3H), 7.46 – 7.31 (m, 6H), 7.14 (t, J = 7.4 Hz, 1H), 6.99 – 6.91 (m, 1H). ^{13}C NMR (75 MHz, DMSO-d_6) δ 170.9, 158.5, 157.9, 144.8, 138.4, 135.4, 133.7, 130.6, 128.8 (2C), 124.1, 120.4 (2C), 118.3, 117.5, 112.8. HPLC-MS (gradient II) $(M + H)^+$ = 297, R_t = 3.31 min (95%).

5-(2-Hydroxyphenyl)-N-phenylfuran-2-carboxamide (**85**). The title compound was prepared by reaction of 5-bromo-*N*-phenylfuran-2-carboxamide (**68**) (3.38 mmol, 900 mg), Na_2CO_3 (7.44 mmol, 789 mg), $Pd(PPh_3)_4$ (0.17 mmol, 196 mg), 2-(4,4,5,5-tetramethyl-1,3,2-dioxaborolan-2-yl)phenol (4.06 mmol, 850 μL) and a solution of toluene (4 mL), H_2O (3 mL) and EtOH (2 mL) as solvent according to procedure B. Purification: Hex/EtOAc (7:3). Yield: 780 mg (83%) as a white solid. Mp 168 – 170 ºC. ^{1}H NMR (300 MHz, DMSO-d_6) δ 10.38 (s, 1H), 10.14 (s, 1H), 8.09 (dd, J = 7.9, 1.7 Hz, 1H), 7.80 – 7.77 (m, 2H), 7.41 – 7.33 (m, 3H), 7.22 (m, 1H), 7.12 (m, 2H), 7.02 – 6.92 (m, 2H). ^{13}C NMR (75 MHz, DMSO-d_6) δ 156.2, 154.4, 152.7, 145.3, 138.5, 129.5, 128.6 (2C), 126.4, 123.8, 120.7 (2C), 119.2, 117.1, 116.3, 116.1, 111.3. HPLC-MS (gradient II) $(M + H)^+$ = 280, R_t = 3.25 min (95%).

N-(4-Chlorophenyl)-5-(2-hydroxyphenyl)furan-2-carboxamide (**86**). The title compound was prepared by reaction of 5-bromo-*N*-(4-chlorophenyl)furan-2-carboxamide (**69**) (3.58 mmol, 1.07 g), Na_2CO_3 (7.88 mmol, 835 mg), $Pd(PPh_3)_4$ (0.18 mmol, 208 mg), 2-(4,4,5,5-tetramethyl-1,3,2-dioxaborolan-2-yl)phenol (4.30 mmol, 900 μL) and a solution of toluene (4 mL), H_2O (3 mL) and EtOH (2 mL) as solvent according to procedure B. Purification: Hex/EtOAc (8:2). Yield: 750 mg (64%) as a white solid. ^{1}H NMR (300 MHz, DMSO-d_6) δ 10.39 (s, 1H), 10.25 (s, 1H), 8.07 (dd, J = 7.9, 1.7 Hz, 1H), 7.81 (d, J = 9.0 Hz, 2H), 7.46 – 7.38 (m, 3H), 7.26 – 7.18 (m, 1H), 7.12 (d, J = 3.6 Hz, 1H), 7.02 – 6.91 (m, 2H). ^{13}C NMR (75 MHz, DMSO-d_6) δ 156.2, 154.4, 152.9, 145.0, 137.5, 129.6, 128.5 (2C), 127.4, 126.4, 122.1 (2C), 119.2, 117.4, 116.2, 116.1, 111.3. HPLC-MS (gradient II) $(M + H)^+$ = 314, R_t = 3.68 min (99%).

N-(2-(1-Benzylpiperidin-4-yl)ethyl)-5-(2-hydroxyphenyl)furan-2-carboxamide (**87**). The title compound was prepared by reaction of *N*-(2-(1-benzylpiperidin-4-yl)ethyl)-5-bromofuran-2-carboxamide (**71**) (3.52 mmol, 1.38 g), Na_2CO_3 (7.75 mmol, 822 mg), $Pd(PPh_3)_4$ (0.176 mmol, 203 mg), 2-(4,4,5,5-tetramethyl-1,3,2-dioxaborolan-2-yl)phenol (4.23 mmol, 860 μL) and a solution of toluene (6 mL), H_2O (4.5 mL) and EtOH (3 mL) as solvent according to procedure B. Purification: CH_2Cl_2/MeOH (95:5). Yield: 1.1 g (80%) as a yellow solid. ^{1}H NMR (300 MHz, DMSO-d_6) δ 10.35 (s, 1H), 8.41 (t, J = 5.8 Hz, 1H), 8.00 (dd, J = 7.9, 1.7 Hz, 1H), 7.34 – 7.19 (m, 6H), 7.11 (d, J = 3.5 Hz, 1H), 7.02 (d, J = 3.5 Hz, 1H), 6.98 – 6.92 (m, 1H), 6.92 – 6.87 (m, 1H), 3.43 (s, 2H), 3.34 – 3.23 (m, 2H), 2.83 – 2.74 (m, 2H), 1.96 – 1.85 (m, 2H), 1.73 – 1.62 (m, 2H), 1.53 – 1.42 (m, 2H), 1.36 – 1.26 (m, 1H), 1.27 – 1.16 (m, 2H). ^{13}C NMR (75 MHz, DMSO- d_6) 157.4, 153.9,

151.4, 145.6, 138.2, 128.9, 128.5 (2C), 127.8 (2C), 126.5, 125.8, 118.8, 116.2, 115.8, 114.9, 110.65, 62.1, 52.9 (2C), 40.1, 35.9, 32.6 (2C), 31.5. HPLC-MS (gradient I) $(M + H)^+ = 405$, $R_t = 3.74$ min (95%).

5-(2-Hydroxyphenyl)-N-phenethylfuran-2-carboxamide (**88**). The title compound was prepared by reaction of 5-bromo-*N*-phenethylfuran-2-carboxamide (**72**) (3.22 mmol, 964 mg), Na_2CO_3 (7.08 mmol, 751 mg), $Pd(PPh_3)_4$ (0.16 mmol, 189 mg), 2-(4,4,5,5-tetramethyl-1,3,2-dioxaborolan-2-yl)phenol (3.86 mmol, 849 mg) and a solution of toluene (4 mL), H_2O (3 mL) and EtOH (2 mL) as solvent according to procedure B. Purification: Hex/EtOAc (7:3). Yield: 850 mg (86%) as a white solid. ^1H NMR (300 MHz, DMSO-d_6) δ 10.30 (s, 1H), 8.55 (t, J = 5.8 Hz, 1H), 8.04 – 7.93 (m, 1H), 7.35 – 7.15 (m, 6H), 7.12 (d, J = 3.5 Hz, 1H), 7.03 (d, J = 3.5 Hz, 1H), 6.99 – 6.88 (m, 2H), 3.56 – 3.42 (m, 2H), 2.95 – 2.79 (m, 2H). ^{13}C NMR (75 MHz, DMSO-d_6) δ 157.7, 154.2, 151.7, 145.8, 139.4, 129.3, 128.6 (2C), 128.4 (2C), 126.1 (2C), 119.1, 116.4, 116.1, 115.4, 110.9, 40.2, 35.4.

5-(3-Hydroxyphenyl)-N-phenylfuran-2-carboxamide (**89**). The title compound was prepared by reaction of 5-bromo-*N*-phenylfuran-2-carboxamide (**68**) (3.76 mmol, 1 g), Na_2CO_3 (4.51 mmol, 478 mg), $Pd(PPh_3)_4$ (0.18 mmol, 217 mg), 3-(4,4,5,5-tetramethyl-1,3,2-dioxaborolan-2-yl)phenol (4.51 mmol, 478 mg) and a solution of toluene (4 mL), H_2O (3 mL) and EtOH (2 mL) as solvent according to procedure B. Purification: Hex/EtOAc (9:1). Yield: 528 mg (50%) as a yellow solid. ^1H NMR (300 MHz, DMSO-d_6) δ 10.17 (s, 1H), 9.67 (s, 1H), 7.81 – 7.72 (m, 2H), 7.42 – 7.35 (m, 5H), 7.32 – 7.23 (m, 1H), 7.18 – 7.07 (m, 2H), 6.85 – 6.77 (m, 1H). ^{13}C NMR (75 MHz, DMSO-d_6) δ 157.8, 156.1, 155.4, 146.5, 138.5, 130.5, 130.0, 128.6 (2C), 123.8, 120.6 (2C), 116.9, 115.9, 115.5, 111.2, 107.8.

Chemical procedures for the synthesis of protected-heterocycle derivatives 32-46, 61, 64 and 90-96 following general procedure C

tert-Butyl 4-(2-(5-(phenylcarbamoyl)thiophen-2-yl)phenoxy)piperidine-1-carboxylate (**32**). The title compound was obtained by reaction of 5-(2-hydroxyphenyl)-*N*-phenyl-thiophene-2-carboxamide (**16**) (3.0 mmol, 886 mg), *tert*-butyl 4-hydroxypiperidine-1-carboxylate (3.6 mmol, 724 mg), DIAD (3.9 mmol, 768 µL), PPh_3 (3.9 mmol, 1.02 g) and 20 mL of THF following general procedure C for 24 h. Purification: Hex/EtOAc (1:1). Yield: 1.0 g (70%) as a white solid. ^{1}H NMR (300 MHz, DMSO-d_6) δ 10.19 (s, 1H), 8.01 (d, J = 4.1 Hz, 1H), 7.86 (dd, J = 7.9, 1.6 Hz, 1H), 7.79 – 7.72 (m, 2H), 7.70 (d, J = 4.1 Hz, 1H), 7.41 – 7.30 (m, 3H), 7.29 – 7.22 (m, 1H), 7.16 – 7.00 (m, 2H), 4.83 (dt, J = 7.8, 4.0 Hz, 1H), 3.71 (ddd, J = 13.5, 6.7, 4.0 Hz, 2H), 3.33 – 3.19 (m 2H), 2.11 – 1.90 (m, 2H), 1.69 (dt, J = 8.7, 5.9 Hz, 2H), 1.42 (s, 9H). ^{13}C NMR (75 MHz, DMSO-d_6) δ 160.2, 153.8, 152.8, 143.6, 138.9, 138.8, 129.6, 128.6, 128.5, 128.2, 125.6, 123.6, 122.3, 121.1, 120.3, 114.2, 78.8, 72.9, 39.5, 30.3, 28.1.

tert-Butyl 4-(2-(5-((4-chlorophenyl)carbamoyl)thiophen-2-yl)phenoxy)piperidine-1-carboxylate (**33**). The title compound was obtained by reaction of *N*-(4-chlorophenyl)-5-(2-hydroxyphenyl)thiophene-2-carboxamide (**17**) (3.61 mmol, 1.19 g), *tert*-butyl 4-hydroxypiperidine-1-carboxylate (3.96 mmol, 798 mg), DIAD (4.69 mmol, 925 µL), PPh_3 (4.69 mmol, 1.23 g) and 20 mL of THF following general procedure C for 24 h. Purification: Hex/EtOAc (1:1). Yield: 296 mg (76%) as a white solid. ^{1}H NMR (300 MHz, DMSO-d_6) δ 10.31 (s, 1H), 7.99 (d, J = 4.1 Hz, 1H), 7.89 – 7.82 (m, 1H), 7.81 – 7.73 (m, 2H), 7.70 (d, J = 4.1 Hz, 1H), 7.46 – 7.36 (m, 2H), 7.38 – 7.31 (m, 1H), 7.31 – 7.22 (m, 1H), 7.08 – 7.01 (m, 1H), 4.87 – 4.79 (m, 2H), 3.77 – 3.64 (m, 2H), 3.32 – 3.19 (m, 2H), 2.04 – 1.92 (m, 2H), 1.77 – 1.59 (m, 2H), 1.41 (s, 9H). ^{13}C NMR (75 MHz, DMSO-d_6) δ 160.3, 153.8, 152.9, 143.9, 138.5, 137.8, 129.7, 128.8, 128.6 (2C), 128.2, 127.3, 125.6, 121.8 (2C), 121.1, 114.2, 78.8, 28.1 (3C).

tert-Butyl 4-(2-(5-((3-chlorophenyl)carbamoyl)thiophen-2-yl)phenoxy)piperidine-1-carboxylate (**34**). The title compound was obtained by reaction of *N*-(3-chlorophenyl)-5-(2-hydroxyphenyl)thiophene-2-carboxamide (**18**) (0.34 mmol, 112 mg), *tert*-butyl 4-hydroxypiperidine-1-carboxylate (0.44 mmol, 89 mg), DIAD (0.44 mmol, 87 µL), PPh_3 (0.44 mmol, 115 mg) and 3 mL of THF following general procedure C for 3 days. Purification: Hex/EtOAc (8:2). Yield: 169 mg (97%) as a white solid. ^{1}H NMR (300 MHz, DMSO-d_6) δ 10.34 (s, 1H), 8.00 (d, J = 4.1 Hz, 1H), 7.95 – 7.91 (m, 1H), 7.89 – 7.83 (m, 1H), 7.71 (d, J = 4.1 Hz, 1H), 7.69 – 7.64 (m, 1H), 7.42 – 7.32 (m, 2H), 7.29 – 7.23 (m, 1H), 7.19 – 7.13 (m, 1H), 7.09 – 7.01 (m, 1H), 4.89 – 4.77 (m, 1H), 3.78 – 3.63 (m, 2H), 3.31 – 3.19 (m, 1H), 2.06 – 1.92 (m, 2H), 1.74 – 1.60 (m, 2H), 1.41 (s, 9H). ^{13}C NMR (75 MHz, DMSO- d_6) δ 160.4, 153.8, 152.9, 144.0, 140.3, 138.3, 132.9, 130.4, 129.8, 128.9, 128.2, 125.6, 123.3, 122.2, 121.1, 119.6, 118.5, 114.2, 78.8, 73.1, 38.7 (2C) 30.4 (2C), 28.1 (3C).

tert-Butyl 4-(2-(5-(phenethylcarbamoyl)thiophen-2-yl)phenoxy)piperidine-1-carboxylate (**35**). The title compound was obtained by reaction of 5-(2-hydroxyphenyl)-*N*-phenethylthiophene-2-carboxamide (**19**) (0.82 mmol, 265 mg), *tert*-butyl 4-hydroxypiperidine-1-carboxylate (1.05 mmol, 211 mg), DIAD (1.05 mmol, 210 µL), PPh_3 (1.05 mmol, 275 mg) and 7 mL of THF following general procedure C for 24 h.

Compound **35** was used in the next step without any further purification. Yield: 237 mg (57%) as a white solid. ^{1}H NMR (300 MHz, DMSO-d_6) δ 8.56 (t, *J* = 5.6 Hz, 1H), 7.79 (dd, *J* = 7.9, 1.6 Hz, 1H), 7.68 (d, *J* = 4.1 Hz, 1H), 7.58 (d, *J* = 4.1 Hz, 1H), 7.37 – 7.17 (m, 7H), 7.02 (ddd, *J* = 8.0, 7.2, 1.2 Hz, 1H), 4.86 – 4.72 (m, 1H), 3.76 – 3.63 (m, 1H), 3.54 – 3.40 (m, 2H), 3.30 – 3.14 (m, 2H), 2.94 – 2.79 (m, 2H), 2.04 – 1.90 (m, 1H), 1.77 – 1.57 (m, 2H), 1.41 (s, 9H). ^{13}C NMR (75 MHz, DMSO-d_6) δ 161.4, 153.8, 152.7, 142.4, 139.4, 139.1, 129.4, 128.7 (2C), 128.4 (2C), 128.2, 127.3, 126.1, 125.4, 122.5, 121.1, 114.2, 78.8, 72.9, 40.8, 35.3, 30.4 (2C), 28.1 (3C).

tert-Butyl 4-(2-(5-((4-morpholinophenyl)carbamoyl)thiophen-2-yl)phenoxy)piperidine-1-carboxylate (**36**). The title compound was obtained by reaction of 5-(2-hydroxyphenyl)-*N*-(4-morpholinophenyl)thiophene-2-carboxamide (**20**) (2.80 mmol, 786 mg), *tert*-butyl 4-hydroxypiperidine-1-carboxylate (3.63 mmol, 729 mg), DIAD (3.63 mmol, 714 µL), PPh_3 (3.63 mmol, 951 mg) and 15 mL of THF following general procedure C for 4 days. Compound **36** was used in the next step without any further purification. Yield: 1.4 g (88%) as a yellow solid. ^{1}H NMR (300 MHz, DMSO-d_6) δ 8.40 (t, *J* = 5.6 Hz, 1H), 7.78 (dd, *J* = 7.9, 1.7 Hz, 1H), 7.68 (d, *J* = 4.0 Hz, 1H), 7.57 (d, *J* = 4.0 Hz, 1H), 7.36 – 7.18 (m, 7H), 7.02 (td, *J* = 7.6, 1.2 Hz, 1H), 4.80 (td, *J* = 7.7, 3.9 Hz, 1H), 3.75 – 3.60 (m, 2H), 3.42 (s, 2H), 3.30 – 3.17 (m, 4H), 2.83 – 2.70 (m, 2H), 2.01 – 1.83 (m, 4H), 1.72 – 1.58 (m, 4H), 1.50 – 1.43 (m, 2H), 1.41 (s, 9H), 1.34 – 1.24 (m, 1H), 1.23 – 1.10 (m, 2H). ^{13}C NMR (75 MHz, DMSO-d_6) δ 161.2, 153.8, 152.7, 142.3, 139.2, 138.7, 129.3, 128.7 (2C), 128.2, 128.1 (2C), 127.1, 126.7, 125.4, 122.5, 121.0, 114.1, 78.8, 72.9, 62.5, 53.2 (2C), 36.7, 36.0, 32.9 (2C), 31.9 (2C), 30.3, 28.1 (3C).

tert-Butyl 4-(2-(5-((2-(1-benzylpiperidin-4-yl)ethyl)carbamoyl)thiophen-2-yl)phenoxy)piperidine-1-carboxylate (**37**). The title compound was prepared by reaction of *N*-(2-(1-benzylpiperidin-4-yl)ethyl)-5-(2-hydroxyphenyl)thiophene-2-carboxamide (**21**) (1.66 mmol, 700 mg), *tert*-butyl 4-hydroxypiperidine-1-carboxylate (2.16 mmol, 434 mg), DIAD (2.16 mmol, 426 µL), PPh_3 (2.16 mmol, 434 mg) and 15 mL of THF following general procedure C for 24 h. Purification: CH_2Cl_2/MeOH (9:1). Yield: 160 mg (16%) as a white solid. ^{1}H NMR (300 MHz, DMSO-d_6) δ 10.02 (s, 1H), 7.94 (d, *J* = 4.1 Hz, 1H), 7.83 (dd, *J* = 7.9, 1.7 Hz, 1H), 7.66 (d, *J* = 4.1 Hz, 1H), 7.58 (d, *J* = 9.0 Hz, 2H), 7.34 (ddd, *J* = 8.8, 7.3, 1.7 Hz, 1H), 7.25 (d, *J* = 8.2 Hz, 1H), 7.06 – 7.02 (m, 1H), 6.96 – 6.92 (m, 2H), 4.89 – 4.75 (m, 1H), 3.78 – 3.72 (m, 4H), 3.72 – 3.66 (m, 2H), 3.30 – 3.21 (m, 2H), 3.11 – 3.04 (m, 4H), 2.03 – 1.94 (m, 2H), 1.71 – 1.62 (m, 2H), 1.41 (s, 9H). ^{13}C NMR (75 MHz, DMSO-d_6) δ 159.7, 153.8, 152.8, 147.5, 143.1, 139.3, 130.9, 129.5, 128.2, 127.9, 125.6, 122.4, 121.5 (2C), 121.1, 115.2 (2C), 114.2, 78.8, 72.9, 66.1 (2C), 48.8 (2C), 39.9 (2C), 30.4 (2C), 28.1 (3C).

tert-Butyl 4-(2-(5-(pyrrolidine-1-carbonyl)thiophen-2-yl)phenoxy)piperidine-1-carboxylate (**38**). The title compound was prepared by reaction of (5-(2-hydroxyphenyl)thiophen-2-yl)(pyrrolidin-1-yl)methanone (**22**) (1.13 mmol, 310 mg), *tert*-butyl 4-hydroxypiperidine-1-carboxylate (1.47 mmol, 297 mg), DIAD (1.47 mmol, 290 µL), PPh_3 (1.47 mmol, 385 mg) and 10 mL of THF following general procedure C for 24 h. Purification: CH_2Cl_2/MeOH (95:5). Yield: 386 mg (75%) as a white solid. ^{1}H NMR (300 MHz, DMSO-d_6) δ 7.81 (dd, *J* = 7.8, 1.6 Hz, 1H), 7.60 (d, *J* = 4.1 Hz, 1H), 7.56 (d, *J* = 4.1 Hz, 1H), 7.36 – 7.29 (m, 1H), 7.27 –

7.21 (m, 1H), 7.06 – 6.99 (m, 1H), 4.83 – 4.73 (m, 8H), 3.84 – 3.70 (m, 2H), 3.70 – 3.61 (m, 1H), 3.54 – 3.44 (m, 2H), 3.30 – 3.17 (m, 0H), 2.02 – 1.80 (m, 6H), 1.72 – 1.59 (m, 2H), 1.41 (s, 9H). ^{13}C NMR (75 MHz, DMSO-d_6) δ 160.7, 153.8, 152.8, 142.1, 138.9, 129.4, 129.0, 128.2, 125.3, 122.4, 121.1, 114.1, 78.8, 72.8, 48.3, 47.1, 40.2 (2C), 30.3 (2C), 28.1 (3C), 23.5.

tert-Butyl 4-(3-(5-(phenylcarbamoyl)thiophen-2-yl)phenoxy)piperidine-1-carboxylate (**39**). The title compound was obtained by reaction of 5-(3-hydroxyphenyl)-*N*-phenylthiophene-2-carboxamide (**23**) (1.70 mmol, 502 mg), *tert*-butyl 4-hydroxypiperidine-1-carboxylate (2.2 mmol, 447 mg), DIAD (2.2 mmol, 437 µL), PPh_3 (2.2 mmol, 582 mg) and 15 mL of THF following general procedure C for 24 h. Purification: Hex/EtOAc (9:1). Yield: 568 mg (70%) as a white solid. ^{1}H NMR (300 MHz, $CDCl_3$) δ 8.0 (s, 1H), 7.68 – 7.58 (m, 3H), 7.41 – 7.26 (m, 4H), 7.26 – 7.21 (m, 1H), 7.19 – 7.12 (m, 2H), 6.91 (d, *J* = 8.0, 1H), 4.54 (dt, *J* = 7.1, 3.6 Hz, 1H), 3.73 (m, 2H), 3.38 (m, 2H), 2.03 – 1.87 (m, 2H), 1.80 (m, 2H), 1.50 (s, 9H). ^{13}C NMR (75 MHz, $CDCl_3$) δ 160.3, 158.1, 155.3, 149.8, 138.4, 138.1, 135.2, 130.7, 129.7, 129.5 (2C), 124.9, 124.2, 120.7 (2C), 119.4, 116.4, 114.5, 80.1, 72.7, 41.0 (2C), 30.9 (2C), 28.9 (3C).

tert-Butyl 4-(3-(5-((4-chlorophenyl)carbamoyl)thiophen-2-yl)phenoxy)piperidine-1-carboxylate (**40**). The title compound was obtained by reaction of *N*-(4-chlorophenyl)-5-(3-hydroxyphenyl)thiophene-2-carboxamide (**24**) (1.71 mmol, 564 mg), *tert*-butyl 4-hydroxypiperidine-1-carboxylate (2.2 mmol, 447 mg), DIAD (2.2 mmol, 437 µL), PPh_3 (2.2 mmol, 582 mg) and 15 mL of THF following general procedure C for 2 days. Purification: Hex/EtOAc (9:1). Yield: 603 mg (69%) as a white solid. ^{1}H NMR (300 MHz, DMSO-d_6) δ 10.35 (s, 1H), 8.01 (d, *J* = 4.0 Hz, 1H), 7.81 – 7.74 (m, 2H), 7.65 (d, *J* = 4.0 Hz, 1H), 7.47 – 7.39 (m, 2H), 7.38 – 7.27 (m, 3H), 7.05 – 6.98 (m, 1H), 4.74 – 4.63 (m, 1H), 3.77 – 3.61 (m, 2H), 3.29 – 3.17 (m, 2H), 2.01 – 1.86 (m, 2H), 1.63 – 1.48 (m, 2H), 1.41 (s, 9H). ^{13}C NMR (75 MHz, DMSO-d_6) δ 159.7, 156.1, 153.9, 148.4, 138.5, 137.7, 134.4, 130.5, 130.4, 128.6 (2C), 127.4, 124.8, 121.8 (2C), 118.4, 116.2, 113.2, 78.7, 71.9, 39.4 (2C), 30.7 (2C), 28.1 (3C).

tert-Butyl 4-(3-(5-((3-chlorophenyl)carbamoyl)thiophen-2-yl)phenoxy)piperidine-1-carboxylate (**41**). The title compound was obtained by reaction of *N*-(3-chlorophenyl)-5-(3-hydroxyphenyl)thiophene-2-carboxamide (**25**) (0.62 mmol, 206 mg), *tert*-butyl 4-hydroxypiperidine-1-carboxylate (0.81 mmol, 163 mg), DIAD (0.81 mmol, 160 µL), PPh_3 (0.81 mmol, 213 mg) and 10 mL of THF following general procedure C for 24 h. Purification: Hex/EtOAc (9:1). Yield: 139 mg (44%) as a white solid. ^{1}H NMR (300 MHz, DMSO-d_6) δ 10.38 (s, 1H), 8.02 (d, *J* = 4.1 Hz, 1H), 7.92 (t, *J* = 2.0 Hz, 1H), 7.70 – 7.64 (m, 2H), 7.44 – 7.28 (m, 4H), 7.20 – 7.15 (m, 1H), 7.06 – 6.99 (m, 1H), 4.73 – 4.62 (m, 1H), 3.75 – 3.61 (m, 2H), 3.29 – 3.15 (m, 2H), 2.02 – 1.87 (m, 2H), 1.64 – 1.47 (m, 2H), 1.41 (s, 9H). ^{13}C NMR (75 MHz, DMSO-d_6) δ 159.8, 157.5, 153.9, 148.6, 140.2, 138.3, 134.3, 132.9, 130.6, 130.4, 124.9, 123.4, 119.6, 118.5, 118.5, 116.2, 113.2, 78.7, 71.9, 39.4 (2C), 30.4 (2C), 28.1 (3C).

tert-Butyl 4-(3-(5-(phenethylcarbamoyl)thiophen-2-yl)phenoxy)piperidine-1-carboxylate (**42**). The title compound was obtained by reaction of 5-(3-hydroxyphenyl)-*N*-phenethylthiophene-2-carboxamide (**26**) (1.39 mmol, 450 mg), *tert*-butyl 4-hydroxypiperidine-1-carboxylate (1.81 mmol, 365 mg), DIAD (1.81 mmol, 357 µL), PPh_3 (1.81 mmol, 475 mg) and 15 mL of THF following general procedure C for 3 days.

Purification: Hex/EtOAc (1:1). Yield: 257 mg (37%) as a white solid. ^{1}H NMR (300 MHz, DMSO-d_6) δ 8.62 (t, J = 5.6 Hz, 1H), 7.71 (d, J = 4.0 Hz, 1H), 7.54 (d, J = 3.9 Hz, 1H), 7.38 – 7.16 (m, 7H), 7.03 – 6.96 (m, 1H), 4.72 – 4.60 (m, 1H), 3.75 – 3.59 (m, 2H), 3.54 – 3.41 (m, 2H), 3.27 – 3.12 (m, 2H), 2.91 – 2.78 (m, 2H), 1.99 – 1.84 (m, 2H), 1.64 – 1.47 (m, 2H), 1.41 (s, 9H). ^{13}C NMR (75 MHz, DMSO-d_6) δ 160.8, 157.5, 153.9, 146.9, 139.4, 139.1, 134.6, 130.5, 128.8, 128.7 (2C), 128.4 (2C), 126.1, 124.6, 118.3, 115.9, 113.1, 78.7, 71.9, 40.8, 35.2, 30.4 (2C), 28.1 (3C).

tert-Butyl 4-(4-(5-(phenylcarbamoyl)thiophen-2-yl)phenoxy)piperidine-1-carboxylate (**43**). The title compound was obtained by reaction of 5-(4-hydroxyphenyl)-*N*-phenylthiophen-2-carboxamide (**27**) (3.61 mmol, 1.06 g), *tert*-butyl 4-hydroxypiperidine-1-carboxylate (3.96 mmol, 798 mg), DIAD (4.69 mmol, 925 µL), PPh_3 (4.69 mmol, 1.23 g) and 20 mL of THF following general procedure C for 24 h. Purification: Hex/EtOAc (7:3). Yield: 811 mg (47%) as a white solid. ^{1}H NMR (300 MHz, DMSO-d_6) δ 10.20 (s, 1H), 8.00 (d, J = 4.0 Hz, 1H), 7.77 – 7.71 (m, 2H), 7.67 (d, J = 8.8 Hz, 2H), 7.50 (d, J = 4.0 Hz, 1H), 7.36 (dd, J = 8.6, 7.3 Hz, 2H), 7.15 – 7.03 (m, 3H), 4.63 (dt, J = 8.1, 4.2 Hz, 1H), 3.72 – 3.63 (m, 2H), 3.24 – 3.17 (m, 2H), 2.02 – 1.81 (m, 2H), 1.54 (dt, J = 8.5, 4.1 Hz, 2H), 1.42 (s, 9H).^{13}C NMR (75 MHz, DMSO-d_6) δ 160.0, 157.7, 154.2, 148.8, 139.0, 137.9, 130.6, 128.9 (2C), 127.5 (2C), 126.1, 123.9, 123.5, 120.6 (2C), 116.7 (2C), 79.0, 72.3, 40.6 (2C), 30.6 (2C), 28.4 (3C).

tert-Butyl 4-(4-(5-((4-chlorophenyl)carbamoyl)thiophen-2-yl)phenoxy)piperidine-1-carboxylate (**44**). The title compound was obtained by reaction of *N*-(4-chlorophenyl)-5-(4-hydroxyphenyl)thiophene-2-carboxamide (**28**) (2.14 mmol, 705 mg), *tert*-butyl 4-hydroxypiperidine-1-carboxylate (2.78 mmol, 559 mg), DIAD (2.78 mmol, 548 µL), PPh_3 (2.78 mmol, 728 mg) and 15 mL of THF following general procedure C for 24 h. Purification: Hex/EtOAc (7:3). Yield: 83 mg (8%) as a white solid. ^{1}H NMR (300 MHz, DMSO-d_6) δ 10.23 (s, 1H), 7.96 – 7.86 (m, 2H), 7.86 – 7.76 (m, 2H), 7.46 – 7.41 (m, 2H), 7.40 (d, J = 3.6 Hz, 1H), 7.14 – 7.06 (m, 2H), 7.03 (d, J = 3.6 Hz, 1H), 4.74 – 4.60 (m, 1H), 3.76 – 3.61 (m, 2H), 3.29 – 3.13 (m, 2H), 1.98 – 1.87 (m, 2H), 1.64 – 1.49 (m, 2H), 1.41 (s, 10H). ^{13}C NMR (75 MHz, DMSO-d_6) δ 157.9, 156.6, 156.1, 154.4, 146.2, 137.9, 129.0 (2C), 127.9, 126.8 (2C), 122.6, 122.5 (2C), 117.9, 116.6 (2C), 106.8, 79.2, 72.4, 41.1 (2C), 30.8 (2C), 28.5 (3C).

tert-Butyl 4-(4-(5-((3-chlorophenyl)carbamoyl)thiophen-2-yl)phenoxy)piperidine-1-carboxylate (**45**). The title compound was obtained by reaction of *N*-(3-chlorophenyl)-5-(4-hydroxyphenyl)thiophene-2-carboxamide (**29**) (0.88 mmol, 290 mg), *tert*-butyl 4-hydroxypiperidine-1-carboxylate (1.14 mmol, 229 mg), DIAD (1.14 mmol, 226 µL), PPh_3 (1.14 mmol, 289 mg) and 5 mL of THF following general procedure C for 24 h. Purification: Hex/EtOAc (9:1). Yield: 252 mg (56%) as a white solid. ^{1}H NMR (300 MHz, DMSO-d_6) δ 10.33 (s, 1H), 7.99 (d, J = 4.0 Hz, 1H), 7.91 (t, J = 2.0 Hz, 1H), 7.72 – 7.62 (m, 3H), 7.51 (d, J = 3.9 Hz, 1H), 7.39 (t, J = 8.1 Hz, 1H), 7.20 – 7.13 (m, 1H), 7.11 – 7.02 (m, 2H), 4.68 – 4.55 (m, 1H), 3.74 – 3.60 (m, 2H), 3.27 – 3.12 (m, 2H), 1.99 – 1.83 (m, 2H), 1.61 – 1.47 (m, 2H), 1.41 (s, 9H). ^{13}C NMR (75 MHz, DMSO-d_6) δ 159.9, 157.5, 153.9, 149.0, 140.3, 137.1, 132.9, 130.7, 130.4, 127.3 (2C), 125.7, 123.3 (2C), 119.6, 118.5, 116.5 (2C), 78.8, 72.0, 30.3 (2C), 28.1 (3C).

tert-Butyl 4-(4-(5-(phenethylcarbamoyl)thiophen-2-yl)phenoxy)piperidine-1-carboxylate (**46**). The title compound was obtained by reaction of 5-(4-hydroxyphenyl)-*N*-phenethylthiophene-2-carboxamide (**30**) (1.69 mmol, 546 mg), *tert*-butyl 4-hydroxypiperidine-1-carboxylate (2.20 mmol, 442 mg), DIAD (2.20 mmol, 434 µL), PPh_3 (2.20 mmol, 576 mg) and 15 mL of THF following general procedure C for 24 h. Purification: Hex/EtOAc (7:3). Yield: 624 mg (73%) as a white solid. ^{1}H NMR (300 MHz, DMSO-d_6) δ 8.56 (t, J = 5.6 Hz, 1H), 7.67 (d, J = 3.9 Hz, 1H), 7.65 – 7.56 (m, 2H), 7.39 (d, J = 3.9 Hz, 1H), 7.35 – 7.17 (m, 5H), 7.07 – 7.00 (m, 2H), 4.61 (dt, J = 8.1, 4.1 Hz, 1H), 3.72 – 3.58 (m, 3H), 3.54 – 3.38 (m, 2H), 3.26 – 3.13 (m, 2H), 2.84 (t, J = 7.4 Hz, 2H), 2.00 – 1.84 (m, 2H), 1.60 – 1.46 (m, 2H), 1.41 (s, 9H). ^{13}C NMR (75 MHz, DMSO-d_6) δ 160.9, 157.2, 153.9, 147.3, 139.4, 137.9, 128.9, 128.6 (2C), 128.3 (2C), 127.1, 126.1, 125.9, 123.0, 116.4 (2C), 78.7, 71.9, 40.7, 35.2, 34.0 (2C), 30.3 (2C), 28.1 (3C).

4-(4-(4,4,5,5-Tetramethyl-1,3,2-dioxaborolan-2-yl)phenoxy)piperidine-1-carboxylate (**61**). The title compound was obtained by reaction of 4-(4,4,5,5-tetramethyl-1,3,2-dioxaborolan-2-yl)phenol (1.36 mmol, 300 mg), *tert*-butyl 4-hydroxypiperidine-1-carboxylate (1.77 mmol, 356 mg), DIAD (1.77 mmol, 349 µL), PPh_3 (1.77 mmol, 465 mg) and 5 mL of THF following general procedure C for 24 h. Purification: Hex/EtOAc (9:1). Yield: 228 mg (42%) as a white solid. ^{1}H NMR (300 MHz, DMSO-d_6) δ 7.59 (d, 2H), 6.96 (d, 2H), 4.69 – 4.54 (m, 1H), 3.75 – 3.54 (m, 2H), 3.26 – 3.10 (m, 2H), 1.99 – 1.81 (m, 2H), 1.60 – 1.45 (m, 2H), 1.40 (s, 9H), 1.27 (s, 12H). ^{13}C NMR (75 MHz, DMSO-d_6) δ 159.6, 153.9, 136.3 (2C), 115.1 (2C), 83.3 (2C), 78.7, 71.6, 30.3 (2C), 28.1 (3C), 24.7 (4C).

5-(2-((1-benzylpiperidin-4-yl)oxy)phenyl)-N-phenylthiophene-2-carboxamide (**64**). The title compound was obtained by reaction of 5-(2-hydroxyphenyl)-*N*-phenylthiophene-2-carboxamide (**16**) (0.203 mmol, 60.1 mg), 1-benzylpiperidin-4-ol (0.264 mmol, 50.5 mg), DIAD (0.264 mmol, 52.1 µL), PPh_3 (0.264 mmol, 69.2 mg) and 5 mL of THF following general procedure C for 3 days. Purification: Hex/EtOAc (6:4). Yield: 37.1 mg (37%) as a yellow solid. Mp 171 – 173 ºC. ^{1}H NMR (300 MHz, DMSO-d_6) δ 10.19 (s, 1H), 8.00 (d, J = 4.1 Hz, 1H), 7.84 (dd, J = 7.9, 1.6 Hz, 1H), 7.80 – 7.72 (m, 2H), 7.69 (d, J = 4.1 Hz, 1H), 7.40 – 7.33 (m, 2H), 7.33 – 7.27 (m, 5H), 7.26 – 7.20 (m, 2H), 7.15 – 7.07 (m, 1H), 7.06 – 6.99 (m, 1H), 4.70 – 4.57 (m, 1H), 3.50 (s, 2H), 2.82 – 2.68 (m, 2H), 2.32 – 2.18 (m, 2H), 2.11 – 1.95 (m, 2H), 1.85 – 1.71 (m, 2H). ^{13}C NMR (75 MHz, DMSO-d_6) δ 160.2, 153.0, 143.7, 138.9, 138.8, 138.5, 129.6 (2C), 128.7, 128.7 (2C), 128.5, 128.2 (2C), 128.1, 126.9, 125.5, 123.6, 122.4, 120.9, 120.3 (2C), 114.3, 73.7, 61.9, 50.2 (2C), 30.7 (2C). HRMS (ESI) m/z: $[M + H]^+$ calc. for $C_{29}H_{30}N_2O_2S$ 469.1944; found 469.1939. HPLC-MS (gradient II) $(M + H)^+$ = 469 , R_t = 3.45 (97%).

tert-Butyl 4-(2-(5-(phenethylcarbamoyl)thiazol-2-yl)phenoxy)piperidine-1-carboxylate (**90**). The title compound was obtained by reaction of 2-(2-hydroxyphenyl)-*N*-phenethylthiazole-5-carboxamide (**83**) (0.75 mmol, 242 mg), *tert*-butyl 4-hydroxypiperidine-1-carboxylate (0.97 mmol, 195 mg), di-(4-chlorobenzyl)azodicarboxylate (DCAD) (0.97 mmol, 356 mg), PPh_3 (0.97 mmol, 254 mg) and 8 mL of THF following general procedure C for 24 h. Purification: Hex/EtOAc (9:1). Yield: 78 mg (20%) as a white solid. ^{1}H NMR (300 MHz, DMSO-d_6) δ 8.76 (t, 1H), 8.41 (s, 1H), 8.32 (dd, J = 6.1 Hz, 1H), 7.53 – 7.45 (m, 1H), 7.39 – 7.20 (m, 6H), 7.14 – 7.08 (m, 1H), 4.98 – 4.88 (m, 1H), 3.86 – 3.74 (m, 2H), 3.52 – 3.42 (m, 2H),

3.29 – 3.17 (m, 2H), 2.89 – 2.79 (m, 2H), 2.14 – 2.01 (m, 2H), 1.76 – 1.67 (m, 2H), 1.42 (s, 9H). ^{13}C NMR (75 MHz, DMSO-*d₆*) δ 163.8, 160.3, 153.9, 142.2, 139.3, 134.9, 131.8, 128.7 (2C), 128.4 (2C), 128.1, 126.2, 121.7, 120.9, 113.9, 78.9, 73.8, 40.8, 35.1, 30.6 (2C), 28.1 (3C).

tert-Butyl 4-(3-(5-(phenylcarbamoyl)thiazol-2-yl)phenoxy)piperidine-1-carboxylate (**91**). The title compound was obtained by reaction of 2-(3-hydroxyphenyl)-*N*-phenylthiazole-5-carboxamide (**84**) (0.84 mmol, 240 mg), *tert*-butyl 4-hydroxypiperidine-1-carboxylate (1.1 mmol, 221 mg), DIAD (1.1 mmol, 217 μL), PPh_3 (1.1 mmol, 288 mg) and 4 mL of THF following general procedure C for 4 days. Purification: Hex/EtOAc (1:1). Yield: 76 mg (19%) as a yellow solid. ^{1}H NMR (300 MHz, DMSO-d_6) δ 10.47 (s, 1H), 8.68 (s, 1H), 7.77 – 7.68 (m, 2H), 7.62 – 7.53 (m, 2H), 7.49 – 7.42 (m, 1H), 7.42 – 7.34 (m, 2H), 7.21 – 7.10 (m, 2H), 4.78 – 4.67 (m, 2H), 3.74 – 3.60 (m, 2H), 3.29 – 3.14 (m, 2H), 1.99 – 1.85 (m, 2H), 1.67 – 1.51 (m, 2H), 1.41 (s, 9H). ^{13}C NMR (75 MHz, DMSO-d_6) δ 170.5, 158.4, 157.5, 153.9, 144.7, 138.3, 135.8, 133.9, 130.8, 128.8 (2C), 124.2, 120.5 (2C), 119.3, 118.8, 113.4, 78.7, 72.1, 30.3 (2C), 28.1 (3C).

tert-Butyl 4-(2-(5-(phenylcarbamoyl)furan-2-yl)phenoxy)piperidine-1-carboxylate (**92**). The title compound was obtained by reaction of 5-(2-hydroxyphenyl)-*N*-phenylfuran-2-carboxamide (**85**) (2.73 mmol, 760 mg), *tert*-butyl 4-hydroxypiperidine-1-carboxylate (3.54 mmol, 710 mg), DIAD (3.54 mmol, 696 μL), PPh_3 (3.54 mmol, 930 mg) and 10 mL of THF following general procedure C for 24 h. Purification: Hex/EtOAc (9:1). Yield: 740 mg (59%) as a white solid. ^{1}H NMR (300 MHz, DMSO-d_6) δ 10.15 (s, 1H), 8.16 (d, J = 6.1 Hz, 1H), 7.80 – 7.73 (m, 2H), 7.44 – 7.32 (m, 4H), 7.29 – 7.22 (m, 1H), 7.17 – 7.06 (m, 3H), 4.73 – 4.72 (m, 1H), 3.79 – 3.64 (m, 2H), 3.27 – 3.14 (m, 2H), 2.07 – 1.93 (m, 2H), 1.75 – 1.58 (m, 2H), 1.41 (s, 9H). ^{13}C NMR (75 MHz, DMSO-d_6) δ 153.8, 153.5, 151.9, 145.6, 138.4, 129.8, 128.6 (2C), 127.1, 123.8, 120.7 (3C), 118.6, 117.0, 113.8, 111.9, 78.8, 72.7, 30.5 (2C), 28.1 (3C).

tert-Butyl 4-(2-(5-((4-chlorophenyl)carbamoyl)furan-2-yl)phenoxy)piperidine-1-carboxylate (**93**). The title compound was obtained by reaction of *N*-(4-chlorophenyl)-5-(2-hydroxyphenyl)furan-2-carboxamide (**86**) (2.23 mmol, 700 mg), *tert*-butyl 4-hydroxypiperidine-1-carboxylate (2.90 mmol, 584 mg), DIAD (2.90 mmol, 570 μL), PPh_3 (2.90 mmol, 761 mg) and 16 mL of THF following general procedure C for 2 days. Purification: Hex/EtOAc (7:3). Yield: 40 mg (4%) as a yellow solid. ^{1}H NMR (300 MHz, DMSO-d_6) δ 10.27 (s, 1H), 8.15 (dd, J = 7.8, 1.7 Hz, 1H), 7.84 – 7.78 (m, 2H), 7.45 – 7.39 (m, 3H), 7.37 – 7.33 (m, 1H), 7.28 – 7.21 (m, 1H), 7.13 – 7.06 (m, 2H), 4.77 (m, 2H), 3.77 – 3.62 (m, 2H), 3.27 – 3.11 (m, 2H), 2.07 – 1.96 (m, 2H), 1.74 – 1.55 (m, 2H), 1.41 (s, 9H). ^{13}C NMR (75 MHz, DMSO-d_6) δ 156.1, 153.8, 153.5, 152.1, 145.3, 137.4, 129.8, 128.5 (2C), 127.5, 127.1, 122.1 (2C), 120.7, 118.6, 117.3, 113.8, 111.9, 78.8, 72.8, 39.5 (2C), 30.5 (2C), 28.1 (3C).

tert-Butyl 4-(2-(5-((2-(1-benzylpiperidin-4-yl)ethyl)carbamoyl)furan-2-yl)phenoxy)piperidine-1-carboxylate (**94**). The title compound was obtained by reaction of *N*-(2-(1-benzylpiperidin-4-yl)ethyl)-5-(2-hydroxyphenyl)furan-2-carboxamide (**87**) (1.73 mmol, 617 mg), *tert*-butyl 4-hydroxypiperidine-1-carboxylate (2.25 mmol, 400 mg), DIAD (2.25 mmol, 440 μL), PPh_3 (2.25 mmol, 520 mg) and 16 mL of THF following general procedure C for 2 days. Purification: CH_2Cl_2/MeOH (95:5). Yield: 250 mg (28%) as a yellow solid. ^{1}H NMR (300 MHz, DMSO-d_6) δ 8.47 – 8.40 (m, 1H), 8.11 – 8.06 (m, 1H), 7.35 – 7.19 (m,

7H), 7.12 (d, *J* = 3.6 Hz, 1H), 7.09 – 7.01 (m, 1H), 6.99 (d, *J* = 3.6 Hz, 1H), 4.83 – 4.71 (m, 1H), 3.76 – 3.64 (m, 2H), 3.44 (s, 2H), 3.29 – 3.17 (m, 4H), 2.85 – 2.71 (m, 2H), 2.05 – 1.87 (m, 4H), 1.72 – 1.57 (m, 4H), 1.52 – 1.31 (m, 12H), 1.24 – 1.15 (m, 2H). ^{13}C NMR (75 MHz, DMSO-d_6) 157.5, 153.8, 153.3, 150.9, 146.2, 129.4, 128.8, 128.1 (5C), 126.8, 120.6, 118.8, 115.3, 113.8, 111.5, 78.8, 72.7, 62.5, 53.2 (2C), 40.2 (2C), 36.2, 32.9, 31.8, 30.5 (2C), 28.1 (2C), 28.1 (3C).

tert-Butyl 4-(2-(5-(phenethylcarbamoyl)furan-2-yl)phenoxy)piperidine-1-carboxylate (**95**). The title compound was obtained by reaction of 5-(2-hydroxyphenyl)-*N*-phenethylfuran-2-carboxamide (**81**) (2.83 mmol, 850 mg), *tert*-butyl 4-hydroxypiperidine-1-carboxylate (3.68 mmol, 740 mg), DIAD (3.68 mmol, 726 µL), PPh_3 (3.68 mmol, 743 mg) and 10 mL of THF following general procedure C for 4 days. Purification: Hex/EtOAc (6:4). Yield: 791 mg (59%) as a yellow oil. ^{1}H NMR (300 MHz, DMSO-d_6) δ 8.57 (t, *J* = 5.8 Hz, 1H), 8.08 (dd, *J* = 7.8, 1.7 Hz, 1H), 7.38 – 7.16 (m, 7H), 7.14 (d, *J* = 3.5 Hz, 1H), 7.11 – 7.03 (m, 1H), 7.00 (d, *J* = 3.5 Hz, 1H), 4.83 – 4.68 (m, 1H), 3.77 – 3.63 (m, 2H), 3.56 – 3.42 (m, 2H), 3.27 – 3.13 (m, 2H), 2.92 – 2.77 (m, 2H), 2.09 – 1.92 (m, 2H), 1.69 – 1.55 (m, 2H), 1.41 (s, 9H). ^{13}C NMR (75 MHz, DMSO-d_6) δ 157.6, 153.8, 153.4, 151.0, 146.1, 139.4, 129.5, 128.6 (2C), 128.4 (2C), 126.7, 126.1, 120.6, 118.8, 115.4, 113.8, 111.6, 78.8, 72.7, 40.2, 35.3, 30.5 (2C), 28.1 (3C).

tert-Butyl 4-(3-(5-(phenylcarbamoyl)furan-2-yl)phenoxy)piperidine-1-carboxylate (**96**). The title compound was obtained by reaction of 5-(3-hydroxyphenyl)-*N*-phenylfuran-2-carboxamide (**89**) (1.79 mmol, 500 mg), *tert*-butyl 4-hydroxypiperidine-1-carboxylate (2.33 mmol, 469 mg), DIAD (2.33 mmol, 459 µL), PPh_3 (2.33 mmol, 611 mg) and 8 mL of THF following general procedure C for 5 days. Purification: Hex/EtOAc/CH_2Cl_2 (5:2:3). Yield: 192 mg (23%) as a yellow solid. ^{1}H NMR (300 MHz, DMSO-d_6) δ 10.20 (s, 1H), 7.81 – 7.71 (m, 2H), 7.60 – 7.50 (m, 2H), 7.45 – 7.33 (m, 4H), 7.21 (d, *J* = 3.6 Hz, 1H), 7.17 – 7.08 (m, 1H), 7.07 – 7.00 (m, 1H), 4.73 – 4.59 (m, 1H), 3.78 – 3.61 (m, 2H), 3.26 – 3.09 (m, 2H), 2.03 – 1.84 (m, 2H), 1.65 – 1.47 (m, 2H), 1.41 (s, 9H). ^{13}C NMR (75 MHz, DMSO-d_6) δ 157.4, 156.0, 154.9, 153.9, 146.7, 138.4, 130.7, 130.2, 128.6 (2C), 123.9, 120.7 (2C), 117.2, 117.0, 115.7, 112.4, 108.3, 78.7, 72.0, 30.4 (2C), 28.1 (3C).

Chemical procedures for the synthesis of protected-heterocycle derivatives 62 and 73-77 following general procedure D

tert-Butyl 4-(4-(5-((2-benzylpiperidin-4-yl)ethyl)carbamoyl)thiophene-2-yl)phenoxy)piperidine-1 carboxylate (**62**). The title compound was obtained by reaction of *N*-(2-(1-benzylpiperidin-4-yl)ethyl)-5-bromothiophene-2-carboxamide (**14**) (0.74 mmol, 300 mg), Na_2CO_3 (1.64 mmol, 163 mg), $Pd(PPh_3)_4$ (0.0.37 mmol, 43 mg), 4-(4-(4,4,5,5-tetramethyl-1,3,2-dioxaborolan-2-yl)phenoxy)piperidine-1-carboxylate (**61**) (0.74 mmol, 300 mg) and a solution of toluene (4 mL), H_2O (3 mL) and EtOH (2 mL) as solvent according to procedure D. Compound **62** was used in the next step without any further purification. Yield: 438 mg (98%) as a yellow solid. ^{1}H NMR (300 MHz, DMSO-d_6) δ 8.47 – 8.36 (m, 1H), 7.68 (d, J = 3.9 Hz, 1H), 7.64 – 7.60 (m, 2H), 7.38 (d, J = 3.9 Hz, 1H), 7.32 – 7.24 (m, 5H), 7.06 – 7.01 (m, 2H), 4.66 – 4.56 (m, 1H), 3.74 – 3.59 (m, 2H), 3.42 (s, 2H), 3.30 – 3.15 (m, 4H), 2.81 – 2.72 (m, 2H), 1.94 – 1.82 (m, 4H), 1.69 – 1.61 (m, 2H), 1.58 – 1.51 (m, 2H), 1.49 – 1.42 (m, 1H), 1.40 (s, 9H), 1.18 – 1.09 (m, 4H). ^{13}C NMR (75 MHz, DMSO-d_6) δ 160.8, 157.2, 153.9, 147.2, 138.7, 138.1, 128.7 (2C), 128.1 (3C), 127.1, 126.7 (2C), 126.0, 123.0, 116.4 (2C), 78.7, 71.9, 62.5, 53.3 (2C), 40.3 (2C), 36.8, 36.0, 31.9 (2C), 30.3, 28.1 (3C), 21.9 (2C).

tert-Butyl 4-(4-(5-(phenylcarbamoyl)thiazol-2-yl)phenoxy)piperidine-1-carboxylate (**73**). The title compound was obtained by reaction of 2-bromo-*N*-phenylthiazole-5-carboxamide (**65**) (0.28 mmol, 79 mg), Na_2CO_3 (0.57 mmol, 60 mg), $Pd(PPh_3)_4$ (0.014 mmol, 17 mg), 4-(4-(4,4,5,5-tetramethyl-1,3,2-dioxaborolan-2-yl)phenoxy)piperidine-1-carboxylate (**61**) (0.28 mmol, 114 mg) and a solution of toluene (2 mL), H_2O (1.5 mL) and EtOH (1 mL) as solvent according to procedure D. Compound **73** was used in the next step without any further purification. Yield: 57 mg (42%) as a brown solid. ^{1}H NMR (500 MHz, DMSO-d_6) δ 10.41 (s, 1H), 8.62 (s, 1H), 7.99 – 7.93 (m, 2H), 7.72 (d, J = 7.6 Hz, 2H), 7.41 – 7.35 (m, 2H), 7.18 – 7.10 (m, 3H), 4.75 – 4.65 (m, 1H), 3.74 – 3.63 (m, 2H), 3.26 – 3.15 (m, 2H), 2.01 – 1.89 (m, 2H), 1.60 – 1.51 (m, 2H), 1.41 (s, 9H). ^{13}C NMR (125 MHz, DMSO-d_6) δ 170.7, 159.4, 158.5, 153.9, 144.7, 138.4, 134.5, 128.7 (2C), 128.3 (2C), 125.4, 124.0, 120.4 (2C), 116.3 (2C), 78.7, 72.2, 40.1 (2C), 30.2 (2C), 28.0 (3C).

tert-Butyl 4-(4-(5-((2-(1-benzylpiperidin-4-yl)ethyl)carbamoyl)thiazol-2-yl)phenoxy)piperidine-1-carboxylate (**74**). The title compound was obtained by reaction of *N*-(2-(1-benzylpiperidin-4-yl)ethyl)-2-bromothiazole-5-carboxamide (**67**) (0.80 mmol, 327 mg), Na_2CO_3 (1.6 mmol, 170 mg), $Pd(PPh_3)_4$ (0.041 mmol, 47 mg), 4-(4-(4,4,5,5-tetramethyl-1,3,2-dioxaborolan-2-yl)phenoxy)piperidine-1-carboxylate (**61**) (0.80 mmol, 323 mg) and a solution of toluene (2.67 mL), H_2O (2 mL) and EtOH (1.33 mL) as solvent according to procedure D. Purification: CH_2Cl_2/MeOH (95:5). Yield: 205 mg (43%) as a white solid. ^{1}H NMR (300 MHz, DMSO-d_6) δ 8.63 (s, 1H), 8.33 (s, 1H), 7.92 – 7.86 (m, 2H), 7.34 – 7.26 (m, 5H), 7.12 – 7.07 (m, 2H), 4.73 – 4.61 (m, 1H), 3.74 – 3.63 (m, 2H), 3.53 (s, 2H), 3.23 (s, 4H), 2.90 – 2.79 (m, 2H), 2.10 – 1.88 (m, 4H), 1.76 – 1.65 (m, 2H), 1.58 – 1.51 (m, 2H), 1.49 – 1.44 (m, 2H), 1.40 (s, 9H), 1.36 – 1.28 (m, 1H), 1.26 – 1.12 (m, 2H). ^{13}C NMR (75 MHz, DMSO-d_6) δ 169.9, 159.7, 159.3, 154.0, 143.6, 134.6, 129.2, 128.3 (2C), 128.2 (4C), 125.5, 116.4 (2C), 78.8, 72.2, 52.9 (2C), 36.9, 35.7, 30.3 (2C), 28.1 (3C).

tert-Butyl 4-(4-(5-(phenylcarbamoyl)furan-2-yl)phenoxy)piperidine-1-carboxylate (**75**). The title compound was obtained by reaction of 5-bromo-*N*-phenylfuran-2-carboxamide (**68**) (1.65 mmol, 440 mg), Na_2CO_3

(3.30 mmol, 350 mg), $Pd(PPh_3)_4$ (0.083 mmol, 97 mg), 4-(4-(4,4,5,5-tetramethyl-1,3,2-dioxaborolan-2-yl)phenoxy)piperidine-1-carboxylate (**61**) (1.65 mmol, 665 mg) and a solution of toluene (4 mL), H_2O (3 mL) and EtOH (2 mL) as solvent according to procedure D. Purification: Hex/EtOAc (7:3). Yield: 580 mg (76%) as a yellow solid. ^{1}H NMR (300 MHz, DMSO-d_6) δ 10.11 (s, 1H), 7.93 – 7.84 (m, 2H), 7.79 – 7.72 (m, 2H), 7.42 – 7.31 (m, 3H), 7.15 – 7.06 (m, 3H), 7.02 (d, J = 3.6 Hz, 1H), 4.71 – 4.61 (m, 1H), 3.74 – 3.62 (m, 2H), 3.28 – 3.12 (m, 2H), 1.99 – 1.88 (m, 2H), 1.61 – 1.47 (m, 2H), 1.41 (s, 9H). ^{13}C NMR (75 MHz, DMSO-d_6) δ 157.4, 156.1, 155.5, 153.9, 145.9, 138.5, 128.6 (2C), 126.3, 123.8, 122.2, 120.6 (2C), 117.1, 116.1 (2C), 106.3, 78.7, 71.9, 40.2 (2C), 30.3 (2C), 28.1 (3C).

tert-Butyl 4-(4-(5-((3-chlorophenyl)carbamoyl)furan-2-yl)phenoxy)piperidine-1-carboxylate (**76**). The title compound was obtained by reaction of 5-bromo-*N*-(3-chlorophenyl)furan-2-carboxamide (**70**) (0.74 mmol, 223 mg), Na_2CO_3 (1.64 mmol, 173 mg), $Pd(PPh_3)_4$ (0.037 mmol, 43 mg), 4-(4-(4,4,5,5-tetramethyl-1,3,2-dioxaborolan-2-yl)phenoxy)piperidine-1-carboxylate (**61**) (0.74 mmol, 300 mg) and a solution of toluene (4 mL), H_2O (3 mL) and EtOH (2 mL) as solvent according to procedure D. Purification: Hex/EtOAc (9:1). Yield: 240 mg (65%) as a yellow solid. ^{1}H NMR (300 MHz, DMSO-d_6) δ 10.25 (s, 1H), 7.93 (t, J = 2.0 Hz, 1H), 7.91 – 7.86 (m, 2H), 7.74 – 7.69 (m, 1H), 7.44 – 7.37 (m, 2H), 7.20 – 7.14 (m, 1H), 7.13 – 7.07 (m, 2H), 7.04 (d, J = 3.7 Hz, 1H), 4.71 – 4.59 (m, 1H), 3.73 – 3.62 (m, 2H), 3.27 – 3.15 (m, 2H), 1.97 – 1.88 (m, 2H), 1.62 – 1.48 (m, 2H), 1.41 (s, 9H). ^{13}C NMR (75 MHz, DMSO-d_6) δ 158.0, 156.7, 156.2, 146.0, 140.5, 133.4, 130.8, 126.8 (2C), 123.9, 122.6, 120.4, 119.3, 118.2, 116.6 (2C), 106.9, 79.2, 72.4, 30.8 (2C), 28.5 (3C).

tert-Butyl 4-(4-(5-((2-(1-benzylpiperidin-4-yl)ethyl)carbamoyl)furan-2-yl)phenoxy)piperidine-1-carboxylate (**77**). The title compound was obtained by reaction of *N*-(2-(1-benzylpiperidin-4-yl)ethyl)-5-bromofuran-2-carboxamide (**71**) (0.74 mmol, 290 mg), Na_2CO_3 (1.64 mmol, 173 mg), $Pd(PPh_3)_4$ (0.037 mmol, 43 mg), 4-(4-(4,4,5,5-tetramethyl-1,3,2-dioxaborolan-2-yl)phenoxy)piperidine-1-carboxylate (**61**) (0.74 mmol, 300 mg) and a solution of toluene (4 mL), H_2O (3 mL) and EtOH (2 mL) as solvent according to procedure D. Purification: CH_2Cl_2/MeOH (9:1). Yield: 57 mg (13%) as a yellow solid. ^{1}H NMR (300 MHz, DMSO-d_6) δ 8.38 (t, J = 5.8 Hz, 1H), 7.85 – 7.78 (m, 2H), 7.31 – 7.28 (m, 5H), 7.11 – 7.08 (m, 1H), 7.08 – 7.03 (m, 2H), 6.91 (d, J = 3.5 Hz, 1H), 3.47 (s, 2H), 3.31 – 3.17 (m, 6H), 2.83 – 2.77 (m, 2H), 1.98 – 1.86 (m, 4H), 1.75 – 1.58 (m, 4H), 1.41 (s, 12H), 1.28 – 1.25 (m, 2H). ^{13}C NMR (75 MHz, DMSO-d_6) δ 157.6, 157.2, 154.5, 153.9, 146.6, 128.9, 128.2 (4C), 125.9 (3C), 122.4, 116.1 (2C), 115.3, 112.9, 111.7, 105.8, 78.8, 71.9, 61.9, 53.2 (2C), 48.6 (2C), 36.1 (2C), 28.1 (3C), 24.7.
